# The persimmon genome reveals clues to the evolution of a lineage-specific sex determination system in plants

**DOI:** 10.1371/journal.pgen.1008566

**Published:** 2020-02-18

**Authors:** Takashi Akagi, Kenta Shirasawa, Hideki Nagasaki, Hideki Hirakawa, Ryutaro Tao, Luca Comai, Isabelle M. Henry

**Affiliations:** 1 Graduate School of Environmental and Life Science, Okayama University, Okayama, Japan; 2 Japan Science and Technology Agency (JST), PRESTO, Kawaguchi-shi, Saitama, Japan; 3 Kazusa DNA Research Institute, Kazusa-Kamatari, Kisarazu, Chiba, Japan; 4 Graduate School of Agriculture, Kyoto University, Kyoto, Japan; 5 Genome Center and Department of Plant Biology, University of California Davis, Davis, California, United States of America; University of Minnesota, UNITED STATES

## Abstract

Most angiosperms bear hermaphroditic flowers, but a few species have evolved outcrossing strategies, such as dioecy, the presence of separate male and female individuals. We previously investigated the mechanisms underlying dioecy in diploid persimmon (*D*. *lotus*) and found that male flowers are specified by repression of the autosomal gene *MeGI* by its paralog, the Y-encoded pseudo-gene *OGI*. This mechanism is thought to be lineage-specific, but its evolutionary path remains unknown. Here, we developed a full draft of the diploid persimmon genome (*D*. *lotus*), which revealed a lineage-specific whole-genome duplication event and provided information on the architecture of the Y chromosome. We also identified three paralogs, *MeGI*, *OGI* and newly identified *Sister of MeGI* (*SiMeGI*). Evolutionary analysis suggested that *MeGI* underwent adaptive evolution after the whole-genome duplication event. Transformation of tobacco plants with *MeGI* and *SiMeGI* revealed that *MeGI* specifically acquired a new function as a repressor of male organ development, while *SiMeGI* presumably maintained the original function. Later, a segmental duplication event spawned *MeGI*’s regulator *OGI* on the Y-chromosome, completing the path leading to dioecy, and probably initiating the formation of the Y-chromosome. These findings exemplify how duplication events can provide flexible genetic material available to help respond to varying environments and provide interesting parallels for our understanding of the mechanisms underlying the transition into dieocy in plants.

## Author summary

Plant sexuality has fascinated scientists for decades. Most plants can self-reproduce but not all. For example, a small subset of species have evolved a system called dioecy, with separate male and female individuals. Dioecy has evolved multiple times independently and, while we do not understand the molecular mechanisms underlying dioecy in many of these species yet, a picture is starting to emerge with recent progress in several dioecious species. Here, we focused on the evolutionary events leading to dioecy in persimmon. Our previous work had identified a pair of genes regulating sex in this species, called *OGI* and *MeGI*. We drafted the whole genome sequence of diploid persimmon to investigate their evolutionary history. We discovered a lineage-specific whole-genome duplication event, and observed that *MeGI* underwent adaptive evolution after this event. Transgenic analyses validated that *MeGI* newly acquired a male-suppressor function, while the other copy of this gene, *SiMeGI*, did not. The regulator of *MeGI*, *OGI*, resulted from a second smaller-scale segmental duplication event, finalizing the system. This study sheds light on the role of duplication as a mechanism that promote flexible genes functions, and how it can affect important biological functions, such as the establishment of a new sexual system.

## Introduction

Most species of flowering plants are hermaphrodite, but a small proportion have genetically determined separate sexes [1]. The rarity of dioecy contrasts with its broad distribution across the flowering plant phylogenetic tree, suggesting multiple independent transitions into dioecy. Our study aimed to understand the molecular and evolutionary mechanisms underlying such changes. Advances in genomic analyses have allowed studies of plant sex chromosomes in a few dioecious plant species including papaya and *Silene* [2–4], and a few genetic sex determining genes have recently been identified, including in the persimmon, kiwifruit, and asparagus [5–7]. Consistent with theoretical models [8, 9], the results indicate that at least one gain-of-function mutation occurred in the evolution of dioecy, creating a dominant gynoecium or androecium suppressor. Data from these species is also consistent with gene duplication events as the first event leading to these gain-of-function mutations, because the redundancy provided by the presence of duplicate copies allows one copy to be neofunctionalized without loss of the original function [10]. Unlike many animal taxa, flowering plants have experienced numerous whole-genome duplication events (WGD) [11], which are thought to have provided opportunities for the appearance of new traits specific to each plant species. For example, functional differentiation between paralogs, which had been derived from whole-genome duplication (WGD), resulted in the establishment of ripening characteristics in tomato fruits [12], and potentially enabled the adaptation to life underwater in seagrass (*Zostera marina*) [13].

Within the large order Ericales, a heterogametic male (XY) sex determination system has evolved independently in at least two genera, *Diospyros* and *Actinidia* [5, 7, 14]. *Diospyros* had evolved a Y-encoded pseudogene called *OGI*, that produces small-RNA, which in turn repress the autosomal feminization gene, *MeGI* [5]. *MeGI* belongs to the HD-Zip1 gene family conserved across angiosperms, but the specific function of *MeGI* to act for repression of male function, or feminization, has not been observed in *MeGI* orthologs from other plants so far [15–17]. Indeed, although *Actinidia* and *Diospyros* are phylogenetically close to each other, the Y-encoded sex determination system in *Actinidia* does not involve the *MeGI* ortholog or another member of the HD-Zip1 family [7]. The existence of *MeGI*, *OGI*, and a third paralog called *Sister-of-MeGI* (*SiMeGI*), which was newly identified in this study, provide the opportunity to investigate both the scale and context of the duplication events that triggered the appearance of a lineage-specific sex determination system in this species. To address this question, we sequenced the genome of Caucasian diploid persimmon, focusing on the lineage-specific duplication events. Evolutionary analyses on the duplicated pairs found a limited numbers of the genes which were potentially neofunctionalized via adaptive evolution after the duplication. Our results provide a potential path from the duplicated paralogs of a HD-Zip1 to dioecy, and shed light on how lineage-specific duplication events contribute to the evolution of a new sex determination system in a plant species.

## Results and discussion

### Draft genome sequencing of Diospyros

**Fig 1:**
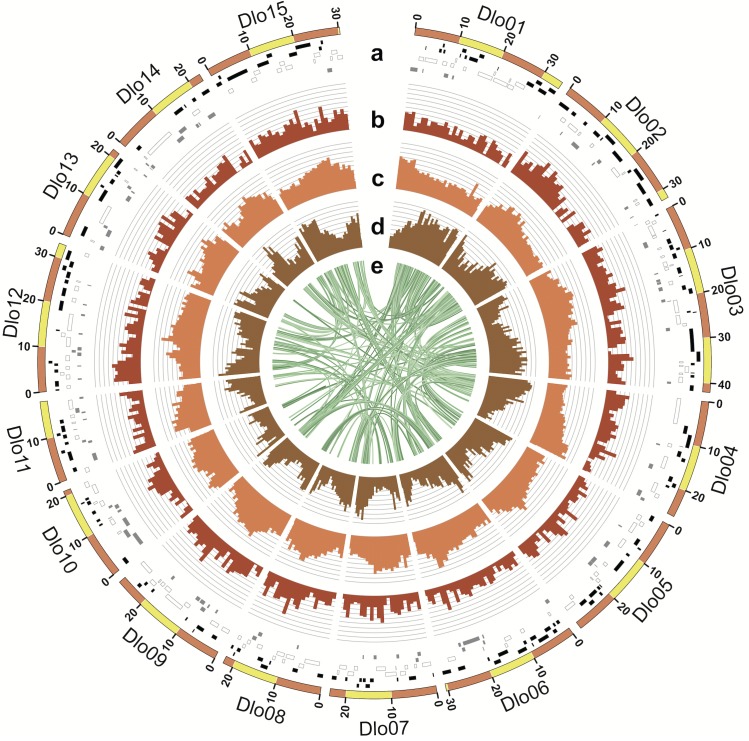
Characterization of the draft persimmon genome. **a**, Fifteen pseudomolecules with the genetically anchored contigs. Black, white and gray bars indicate the positions of the original contigs that were assembled in forward, reverse, or unknown direction respectively. **b**, Relative SNP density in the KK population. **c**, Relative density of repetitive sequences **d**, Relative gene density. **e**, Syntenic relationships within the persimmon genome.

Initially, we assembled a draft genome from ca 65X PacBio long read coverage of the expected haploid genome size (907Mb from flow cytometry [18], 877.7Mb from kmer analysis) using Falcon (S1 Fig, S1 Table). This resulted in 3,073 primary contigs totaling 746.1Mb, which covers ca 85% of the genome, and 5,901 “secondary” contigs, which are putative allelic contigs to the primary contigs. Next, we built three genetic maps, created from two segregating F1 populations (N = 314 and 119, see Materials and Methods and S2 Table). These maps were created from a total of 5,959 markers derived from GBS/ddRAD sequencing and allowed for the anchoring of the ca 61.8% scaffolds into 15 pseudomolecules (Fig 1, S1 Fig, S2 and S3 Tables).

To start characterizing this newly assembled genome, we documented sequence variation between female and male individuals of *D*. *lotus* and content and type of repeat sequences of the draft sequence compared to other sequences eudicots (Fig 1B and 1C, S3 and S4 Tables). Mapping of transcriptome data to this draft genome resulted in 40,532 predicted gene locations (Fig 1D, S1 Dataset). These numbers are similar to results from other asterid plant species, such as tomato (N = 34,879) [12] or kiwifruit (N = 39,040) [19] (S2 and S3 Figs). Of these primary genes, we selected 12,058 which were determined to be either unique or low copy number within the genome (see Materials and Methods).

### Identification of a whole-genome duplication event specific to the Diospyros genus

**Fig 2:**
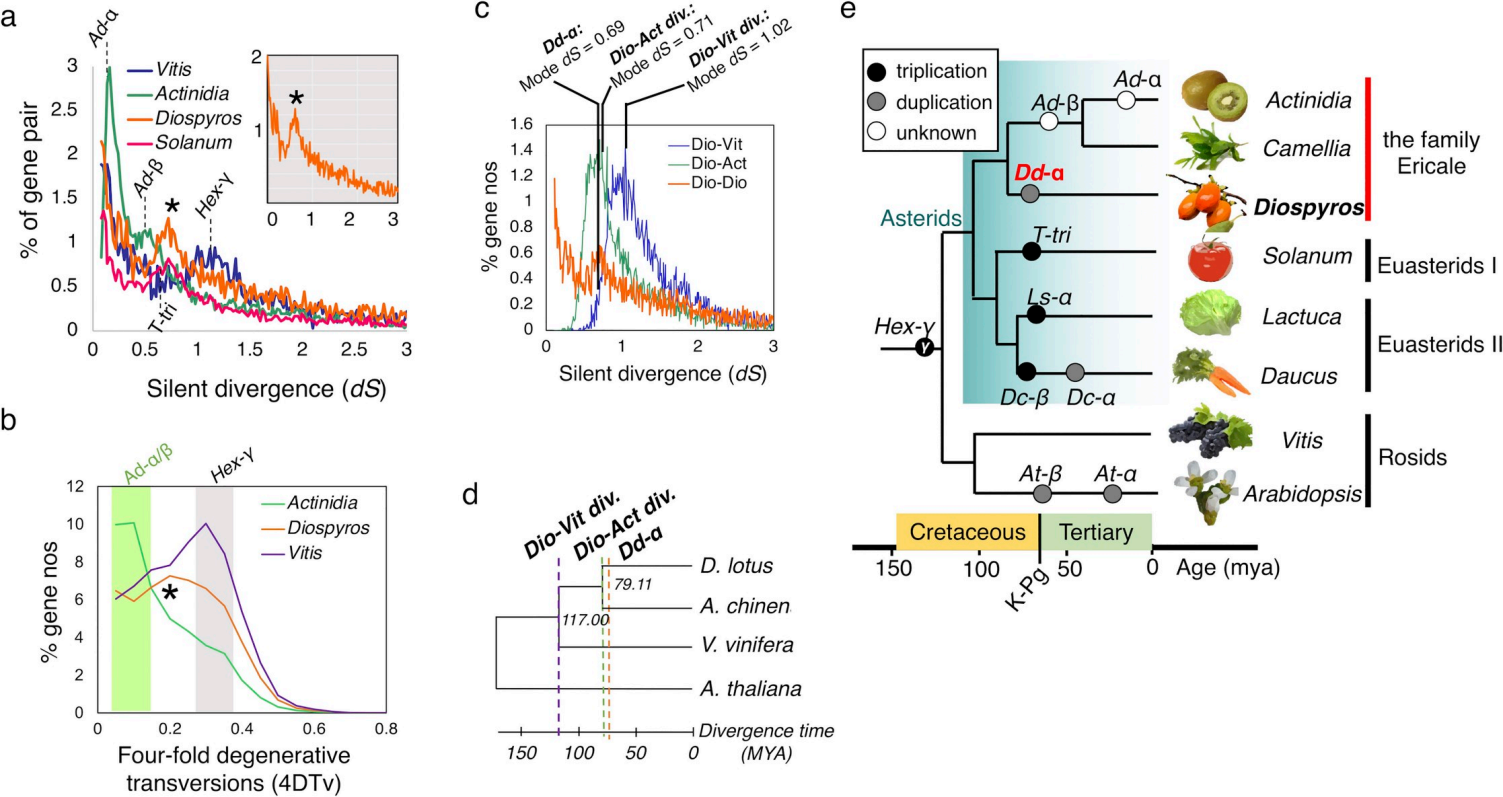
Characterization of lineage-specific whole-genome duplication events. **a**, Distribution of silent divergence rates between homologous gene pairs within the *Diospyros*, *Actinidia*, *Solanum*, and *Vitis* genomes. *Diospyros* shows a peak, indicated by an asterisk, at the same *dS* value as the *Solanum* triplication (T-tri), indicating the concurrent whole-genome duplication events. **b**, Comparison of the 4-fold degenerative transversion rates (4DTv) between the putative paralogous gene pairs, in the *Diospyros*, *Actinidia*, and *Vitis* genomes. Consistent with the distribution of *dS* values, a peak, which corresponds to *Dd*-α, was detected specifically in the persimmon genome, as indicated by an asterisk (*). In the *Actinidia* and *Vitis* genomes, peaks putatively corresponding the *Ad-α/β* and the hexaploidization-γ, were detected, as shown by the green and gray bands, respectively. **c**, Comparison of the *dS* values between the paralogous pairs in the *Diospyros* genome (orange), and the *dS* values between the orthologs in *Diospyros* and *Actinidia* (green), and in *Diospyros* and *Vitis* (purple). **d**, Estimated divergence time between *Diospyros*, *Actinidia*, and *Vitis*, with Arabidopsis as the outgroup. The concatenated sequences of 175 conserved genes across these species were used to determine divergence time, based on the previous estimated divergence of *Actinidia* and *Vitis* at 117MYA in the TIMETREE database (http://www.timetree.org). **e**, Summary of the lineage-specific WGD events in the asterids. The time scale is estimated from *dS* values and previous reports [21–23]. K-Pg, Cretaceous-Paleogene boundary.

To investigate gene duplication patterns, we analyzed the distribution of silent divergence rate (*dS*) between homologous gene pairs. We compared the distribution of silent divergence rate of homologous gene pairs within the persimmon genome, with those within the kiwifruit (*Actinidia*), tomato (*Solanum*) and grape (*Vitis*) genomes. A subset of persimmon genes formed a clear peak of silent divergence rate (Fig 2A, *dS* = ca 0.5–0.9, mode *dS* = 0.69), suggesting that a whole-genome duplication (WGD) event, named *Dd*-α, occurred in this clade. Next, we performed a genome-wide synteny analysis, based on the location of the gene pairs with *dS* values ranging from 0.5 and 0.9, using SynMap in CoGe [20] (https://genomevolution.org/coge/). The results indicated long syntenic blocks throughout the persimmon genome (S4A and S4B Fig). The genomic regions including the gene pairs in this peak exhibited long regions of synteny (S5 Fig). The distribution of four-fold synonymous (degenerative) third-codon transversion (4DTv) supported this lineage-specific WGD (Fig 2B). Comparison of intraspecific *dS* between homologous gene pairs in the *Diospyros* genome and interspecific *dS* between the orthologs from *Diospyros* and *Actinidia*, or from *Diospyros* and *Vitis*, indicated that the *Dd*-α event postdated the divergence of *Diospyros* and *Actinidia*, and might coincide with the divergence of the Ebenaceae family (Fig 2C and 2D). Two other events, *Ad-α* and *Ad-β*, have been inferred by a similar analysis in the *Actinidia* genome [19] (S6 Fig) but are not detectable in the *Diospyros* genome. Thus, *Actinidia* and *Diospyros* differ by at least three lineage-specific ancestral WGD events. These occurred at a time similar to previously reported whole-genome duplication events in the asterids [19, 21, 22], as well as across the angiosperms [11, 23], concentrated around the K-Pg (Cretaceous-Paleogen) boundary (Fig 2E).

### Only a few gene, including MeGI, exhibit signs of positive selection but divergent expression patterns are common following the WGD event

**Fig 3:**
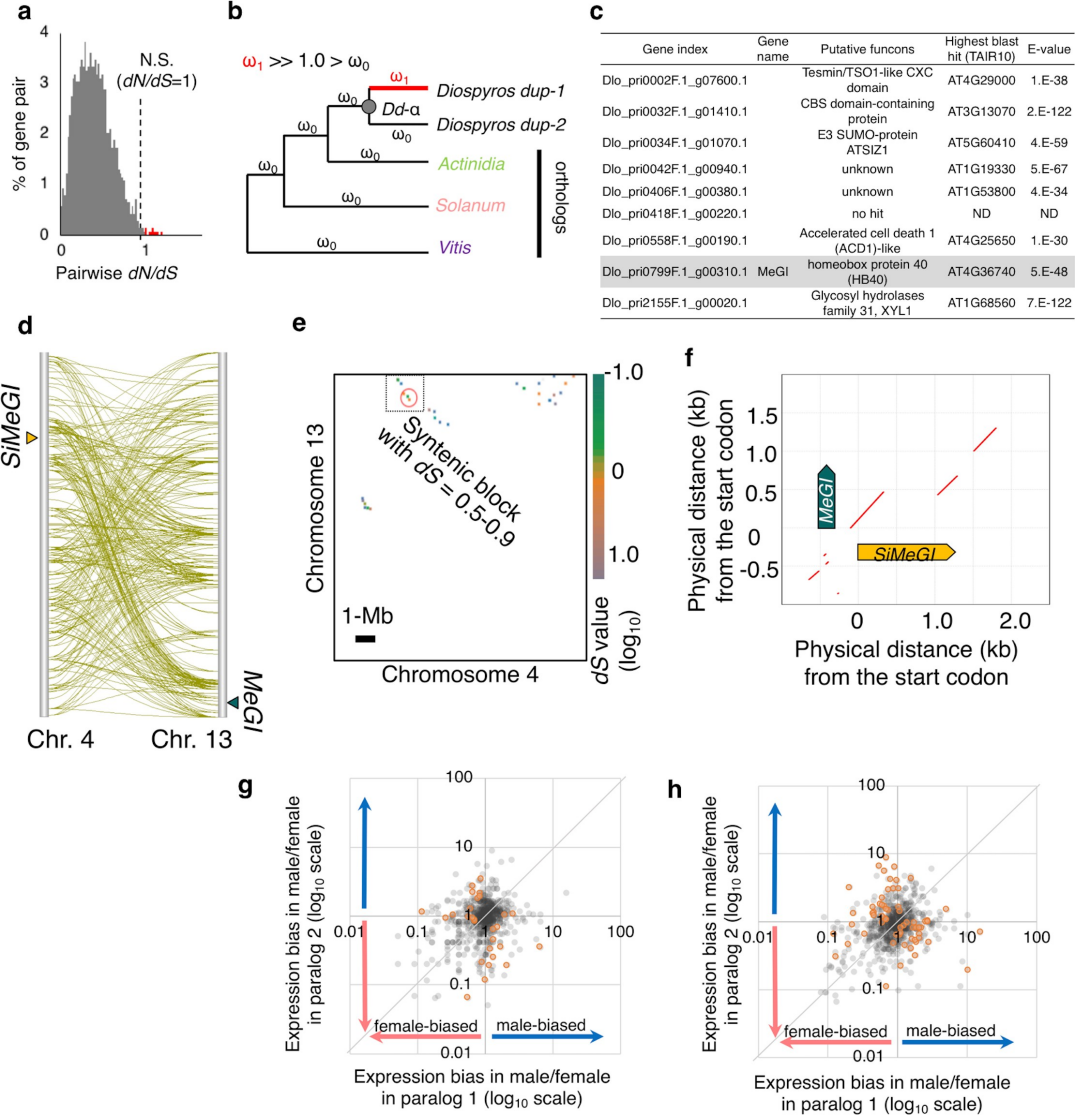
Fate of paleoduplicated genes in *Diospyros*. **a**, Distribution of the pairwise *dN/dS* values in the *Dd-α*-derived paralogous gene pairs, from the alignment of the full ORF sequences. Most gene pairs are under purifying selection (*dN/dS* < 1.0), while only approximately 0.3% of the gene pairs (shown in red) exhibited neutral selection (N.S.) or weak positive selection (*dN/dS* ~ 1.0). **b**, model for the detection of the genes that underwent significant site-branch specific positive selection (posterior probability > 0.99 in Bayes Empirical Bayes method) after *Dd*-α, using *Actinidia*, *Solanum*, and *Vitis* as outgroups. **c**, Functional annotation of the 9 genes that underwent significant site-branch specific positive selection after *Dd*-α. *MeGI* is highlighted in gray. **d**, Inter-chromosomal collinearity between Chr. 4 and Chr. 13. Genes pairs showing significant similarity (<e^-100^ in blastp) are linked (green lines). The segments surrounding *SiMeGI* and *MeGI* exhibit syntenic collinearity. **e**, Synteny analysis of chromosomes 4 and 13, based on gene order using SynMap (CoGe). The dotted rectangle highlights blocks of gene pairs with *dS* values ranging between approximately 0.5 and 0.9, including the *MeGI*-*SiMeGI pair*. The *MeGI*-*SiMeGI* syntenic region is indicated by a red circle, a more detailed figure is available in Supplementary S7 Fig. **f**, Microsynteny analysis of the genomic fragments including *SiMeGI* and *MeGI*, using promer in MUMmer. **g-h**, Comparison of the expression patterns of paralog pairs derived from the *Dd*-α event, focusing on the sex differentiation stages. The ratio of expression levels in male versus female developing flowers (**g**) and mature flowers (**h**) were compared in the paralogs putatively derived from the *Dd*-α WGD event. The ratios were expressed in log_10_ scale. Approximately 10% of the gene pairs exhibited a statistically significant (*P* < 0.01, 2x2 Fisher’s exact test, orange circles) expression bias between the two paralogs (S3 Dataset), and 18.5% of the gene pairs (*N* = 242) showed >5-fold differences between the two paralogs.

To explore the evolutionary significance of lineage-specific duplications, and particularly of the *Dd-α* WGD event, *dN/dS* values between the duplicated gene pairs putatively derived from the *Dd-α* WGD events (N = 2,619) were calculated. The *dN/dS* values averaged over the coding regions indicated that most of the duplicates experienced either purifying or neutral selection (*dN/dS* ≤ 1.0, Fig 3A). In contrast, site- and evolutionary branch-specific tests for positive selection (dN/dS >> 1.0), using PAML, suggested that at least 9 genes experienced strong positive selection (posterior probability > 0.99 in Bayes Empirical Bayes analysis) following the *Dd-α* WGD event (Fig 3B and 3C). Importantly, *MeGI* and its paralog, named *Sister of MeGI* (*SiMeGI*), were one of these 9 gene pairs. Consistently, *MeGI* and *SiMeGI* were included in the same gene family after OrthoMCL analysis. They are located on Chr 13 (Dlo_pri0799F.1) and Chr 4 (Dlo_pri0025F.1), respectively, and these regions showed syntenic collinearity around these genes based on sequence similarity (Fig 3D–3F, S7 Fig). Syntenic blocks derived from gene order (with genes with *dS* values between 0.5 and 0.9) were observed with SynMap in CoGe as well (Fig 3E). These findings are consistent with the hypothesis that they were derived from the *Dd-α* WGD event, although we cannot exclude the possibility that they were generated from a simple segmental duplication concurrent with the *Dd-α*.

In contrast to very small number of genes exhibiting positive selection, a larger proportion of the gene pairs derived from the *Dd-α* WGD events exhibited significant differences in expression patterns. We described expression patterns in male and female buds/flowers using transcriptome data from 8 time points throughout the annual cycle (see Materials and Methods for details). Our results suggest that 45.5% of the gene pairs (597/1,311 pairs) showed significant differentiation (Pearson product-moment correlation test *r*^2^ < 0.3, S2 Dataset). To investigate differences in expression pattern between male and female flowers throughout development, we conducted 2x2 Fisher’s exact test on the *Dd-α*-derived gene pairs (see Materials and Methods) and identified 36 and 65 gene pairs (of 1,311 pairs) exhibiting significant differentiation (*p* < 0.01) in expression patterns between male and female flowers at developing and maturing stages, respectively (Fig 3G and 3H, S3 Dataset). These might have potentially contributed to the establishment of *Diospyros*-specific sex determining mechanisms. Such frequent variation in expression patterns is consistent with previous results in soybean [24] and could have originated from rapid evolution in *cis*-motifs after WGD.

### Adaptive evolution of MeGI to act specifically for repression of androecium development

**Fig 4:**
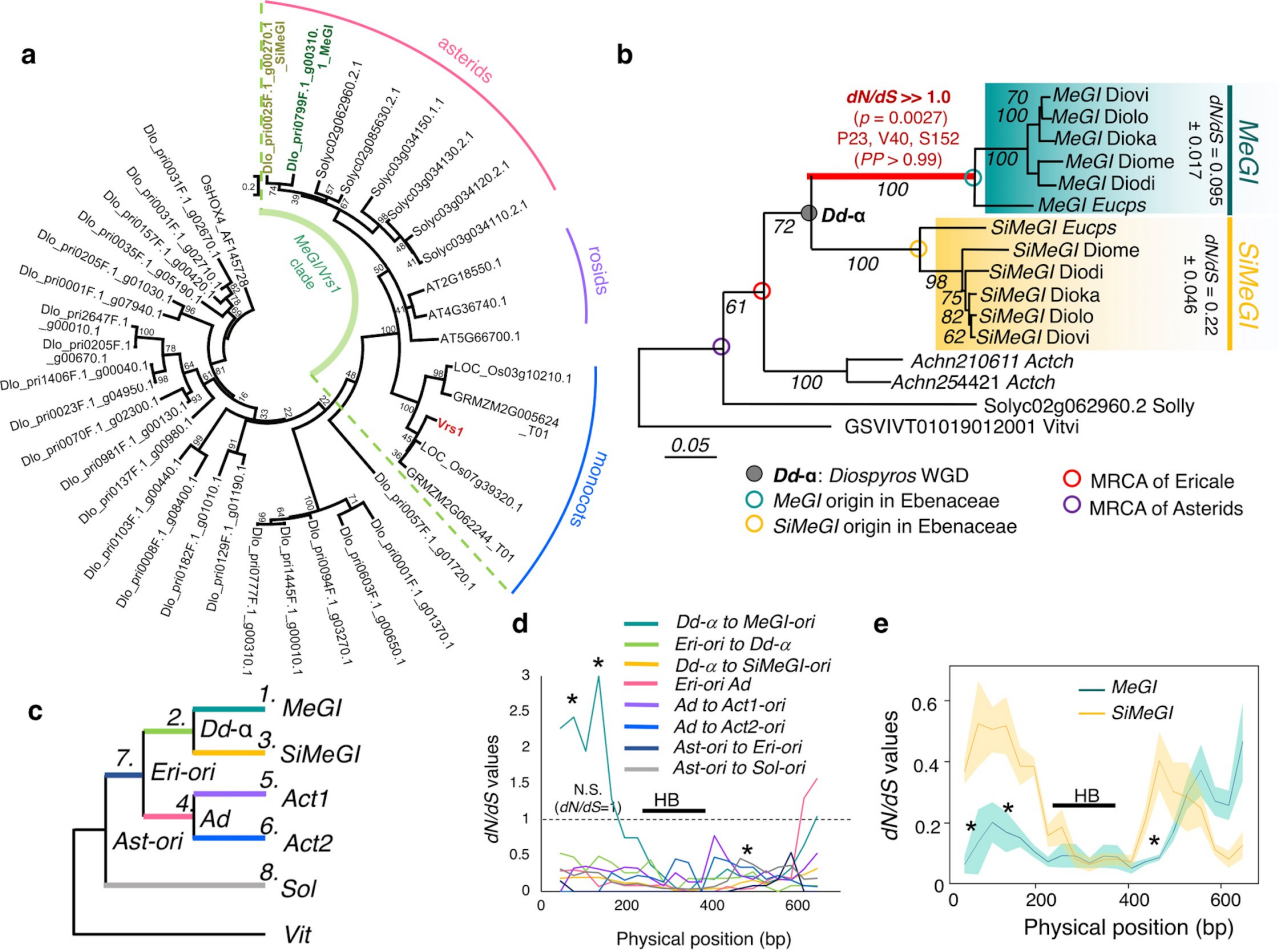
Lineage-specific adaptive evolution of *MeGI*. **a,** Phylogeny of the HD-Zip1 type homeodomain genes in the *D*. *lotus* genome. Only *MeGI* and *SiMeGI* were nested within the *MeGI/Vrs1*-clade with statistically significant support (100/100 and 74/100 for the divergence of *MeGI/Vrs1* clade and *MeGI/SiMeGI* subclade in *D*. *lotus*, respectively). **b**, Divergence of the *MeGI/SiMeGI*-like orthologs in the asterids and evidence of strong positive selection immediately after the *Dd-α* WGD event in *Diospyros* species (colored in red). No significant positive selection was detected elsewhere in this tree. Pairwise *dN/dS* values within the current *MeGI* (0.095) and *SiMeGI* (0.22) sequences suggest that both genes have been functionally fixed. **c**, Branch-specific *dN/dS* rates sliding window analysis of *MeGI/SiMeGI*-like genes from various asterid species. *MeGI* specifically exhibits positive selection in the 5’ region (~0-170bp). The three asterisks indicate the positions of the positively selected sites according to the site-branch specific detection analysis performed using PAML. The position of the homeobox domain (HB) is indicated by the thick black line. **d**, Sliding window assessment of the pairwise *dN/dS* values in the current *MeGI* and *SiMeGI* alleles. All three of the positions positively selected in *MeGI* sites after the *Dd-α* WGD event (asterisks) are under stronger purifying selection in *MeGI* than in *SiMeGI*, consistent with a situation of an adaptive evolution utilizing the mutations positively selected after WGD.

Genome-wide survey of the HD-Zip1 family, to which *MeGI* belongs, found 34 genes in the *D*. *lotus* genome. Phylogenetic analysis of *MeGI/Vrs1* orthologs from representative angiosperm species indicated that only *MeGI* and *SiMeGI* belong in the *MeGI/Vrs1* clade (bootstrap = 100/100, Fig 4A, S8 Fig). Finer evolutionary analysis on the *MeGI/SiMeGI* orthologs, to detect site-branch specific evolutionary rates using PAML, indicated that specific regions of *MeGI* experienced strong positive selection soon after the *Dd-α* event (Fig 4B, p = 0.0027 for *dN/dS* > 1.0, post. prob. > 0.99 for P23-V40-S152, and Fig 4C and 4D, *dN/dS* > 2.0 for the region between 45 and 165 bp in the sliding window test).

On the other hand, *MeGI* experienced strong purifying selection overall (average *dN/dS* = 0.095) since the establishment of the Ebenaceae (*Euclea* and *Diospyros*) (Fig 4B). Furthermore, the regions that experienced positive selection early are currently under stronger purifying selection in *MeGI* than in *SiMeGI* (Fig 4E). This is also consistent with the idea that *MeGI* first underwent neofunctionalization following the whole-genome duplication event, and that these changes were later fixed by positive selection. On the other hand, stronger purifying selection in *MeGI* than in *SiMeGI* could reflect lesser functional importance of *SiMeGI* (or possibly that it is degenerating since the whole-genome duplication occurred). Alternatively, it could reflect the need to conserve high sequence homology between *OGI* and *MeGI* in order to maintain the regulatory role of *OGI* via smRNA targeting *MeGI*.

**Fig 5:**
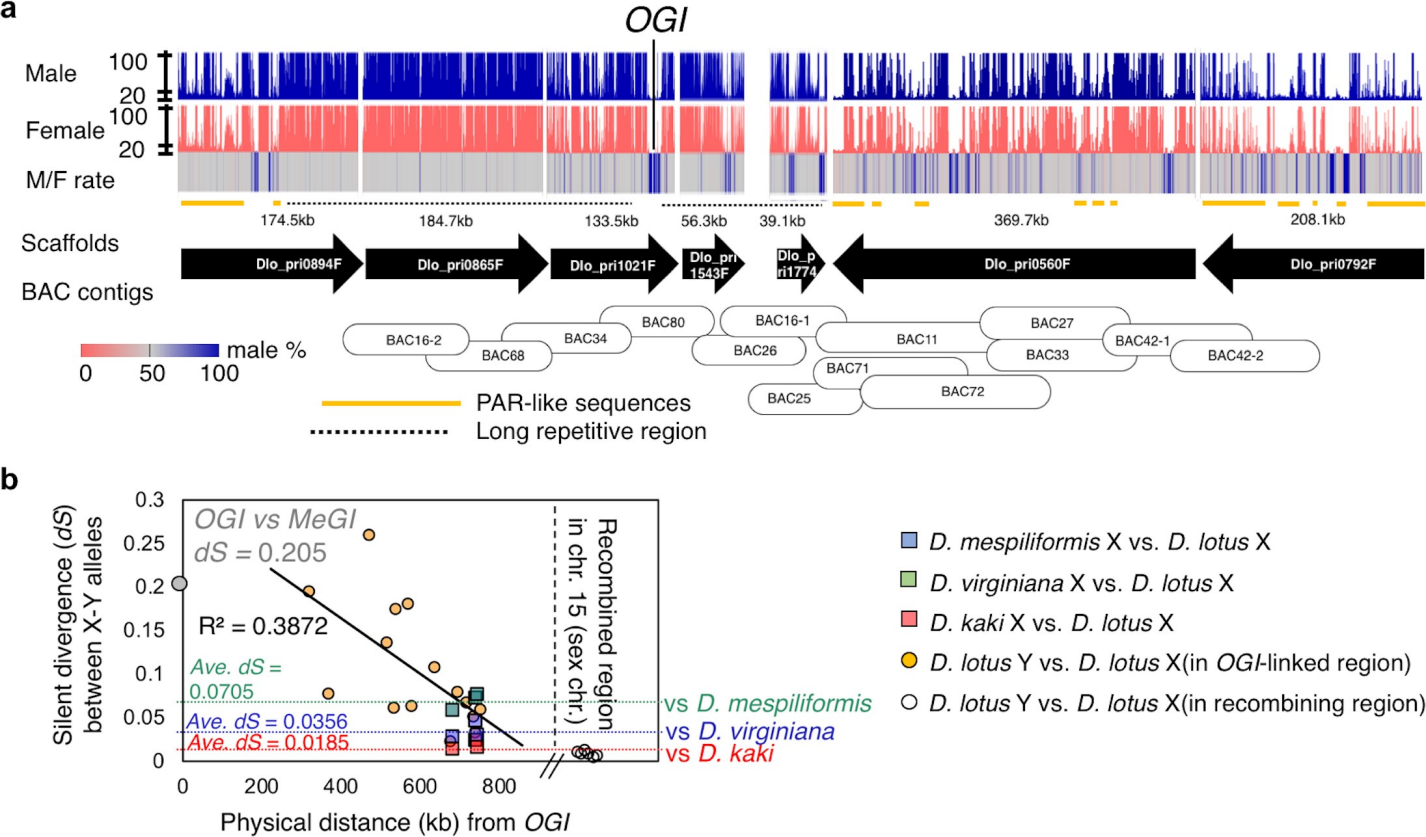
Functional differentiation between *MeGI* and *SiMeGI*. **a-h**, *N*. *tabacum* transgenic lines expressing either of *MeGI* or *SiMeGI* under the control of the 35S promoter. The lines expressing *MeGI* (**a-c**) showed rudimental anthers (**a**) which did not produce functional pollen grains (**b**), and severe dwarfism with chlorophyll starvation and narrow leaves (**c**, see S7 Fig for the detail). The lines expressing *SiMeGI* (**d-f**) developed regular anthers (**d**) which produced fertile pollen (**e**), and showed moderate dwarfism (**f**). pis: pistil, ra: rudimental anthers, an: anthers. **g-h**, Both *MeGI*- and *SiMeGI*-overexpressing lines were phenotypically different from the control plants transformed with empty vectors (cont), but the *MeGI*-expressing lines exhibited more severe departure from the WT controls for specific traits, such as leaves width (see S9 Fig). Bars indicate 5mm for a and d, 50mm for c, f, g, and h. **i-j,** expression patterns of *MeGI*, *SiMeGI*, and *PI*, with *actin* as a positive control, in the transgenic lines transformed with CaMV35S-*MeGI* (i) and CaMV35S-*SiMeGI* (j). **k**, DNA motifs identified as preferentially bound to following transcription factors all nested within the *MeGI/SiMeGI* clade: *MeGI* [25], *SiMeGI* (our experiments), and three Arabidopsis HD-ZIP1 genes [29], using DAP-Seq analyses (see Methods). **l-n**, expression patterns of *MeGI* and *SiMeGI* in buds and flower primordia were highly correlated (Pearson’s *r* > 0.7). Expression levels in female (**l**) and male (**m**) are expressed as RPKM values. **n**, Developmental stages.

Consistent with the evolutionary analysis presented above, ectopic expression of *MeGI* or *SiMeGI* in *Nicotiana tabacum* indicate differentiation of their protein functions. Constitutive induction of *MeGI* under the control of the CaMV35S promoter resulted in severely dwarfed plants and repressed androecium development (Fig 5A–5C, 5G and 5H, S9 Fig, S5 Table), consistent with previous results using the same construct in *A*. *thaliana* [5]. On the other hand, constitutive induction of *SiMeGI* under the control of the same promoter resulted in plants of only slightly reduced stature and normal androecium development in *N*. *tabacum* (Fig 5D–5H, S9 Fig, S6 Table). The function of *MeGI* as repressor of androecium in persimmon is due to the ability to regulate *PISTILLATA* (*PI*) in young developing androecium [25]. The expression level of *PI* in *N*. *tabacum* was significantly down-regulated in the transgenic lines with *MeGI*, while the lines transformed with *SiMeGI* showed no changes in *PI* expression (Fig 5I and 5J). In Arabidopsis, which is a very far lineage from *Diospyros*, high expression of *SiMeGI* typically did not result in altered flower morphology although it occasionally resulted in inhibited androecium development (S10 Fig, S7 and S8 Tables).

Taken together, our results are consistent with the hypothesis that a role in androecium development is specific to *MeGI*. This is further supported by the fact that mutants of the *MeGI/SiMeGI* orthologs which are normally expressed in flower primordia in other angiosperm species, do not affect androecia development [15–17]. Our evolutionary analyses revealed that the positive selection that affected *MeGI* specifically did not occur on the region binding to the target *cis*-motifs, called homeobox-domain (HB) (Fig 4D and 4E), but rather on the 5’ undefined region and on the leucine zipper region putatively forming heterodimers [26, 27]. This was supported by the results of DNA affinity purification sequencing (DAP-Seq) [28] using *MeGI* [25] or *SiMeGI* fused to a Halo-tag. This allowed us to identify which genes and/or motifs is preferentially targeted by each of these transcription factors. The DAP-Seq reads were mapped to the *D*. *lotus* genome to characterize the accumulated recognition motifs (see Materials and Methods). We identified the motifs using the top 1,000 high-confidence peaks, and determined that the AATWATT sequence was enriched when using MeGI [25] and SiMeGI as the probes (Fig 5K). This motif is commonly recognized by the Arabidopsis HD-ZIP1 genes as well [25, 29]. Thus, it is possible that the feminization role of *MeGI* could have resulted from either increased efficiency or novel affinity to interact with other factors. Finally, the native expression patterns of *MeGI* and *SiMeGI* in persimmon are also slightly different in developing buds and flower primordia (Fig 5L–5N, S11 and S12 Figs). Specifically, *MeGI* exhibits higher expression than *SiMeGI* during the flower maturing stages (Fig 5L–5N). This expression differentiation might also contribute to *MeGI-*specific feminizing function.

### Formation of a lineage-specific, and slowly evolving Y-chromosome in Diospyros

**Fig 6:**
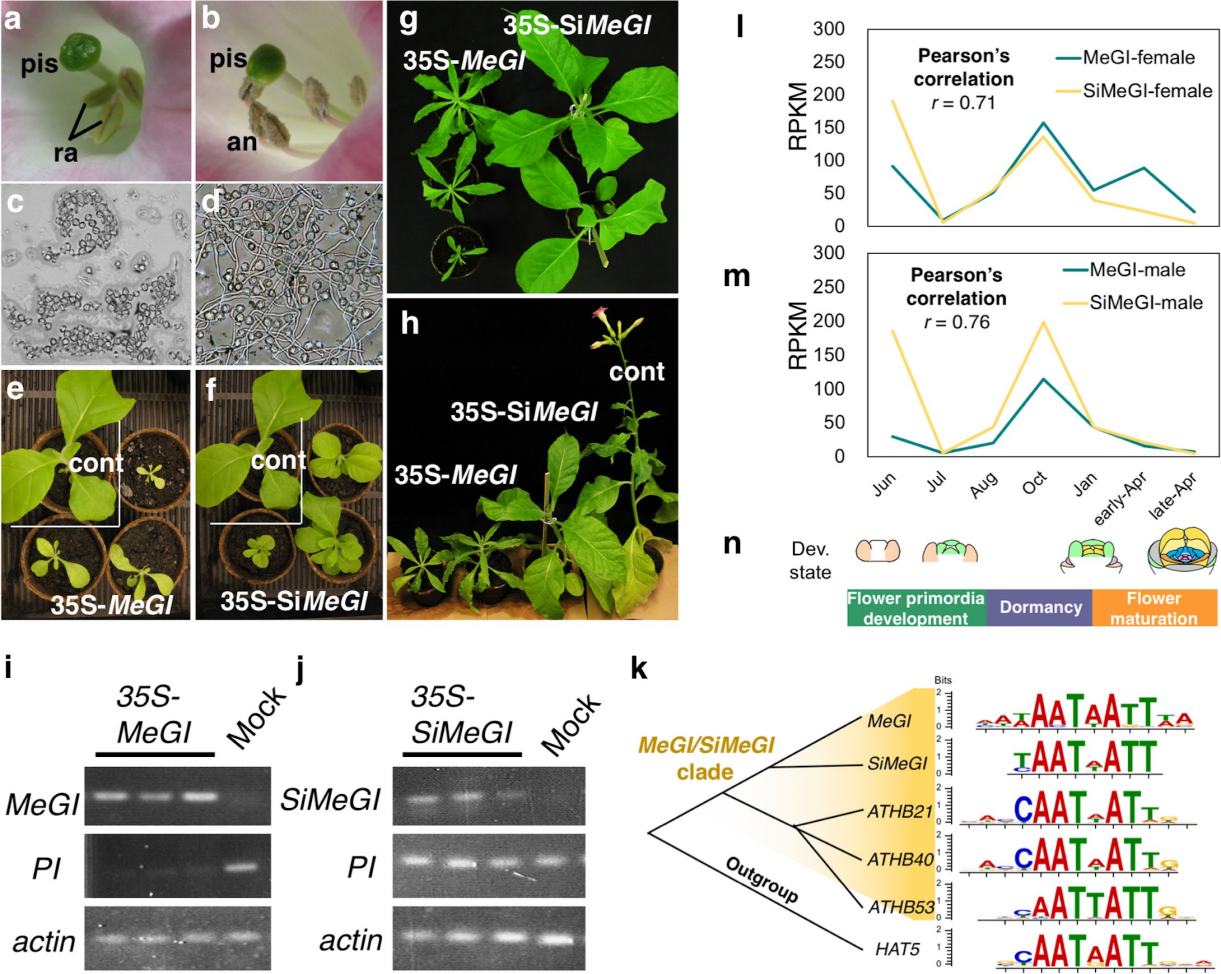
Genomic context of the Y-chromosomal region surrounding *OGI*. **a**, Read coverage from male (blue) and female (pink) samples and male/female coverage ratio across the scaffolds covering the male-specific region of the Y-chromosome. For both the male and female reads, expected coverage a single-copy sites is approximately 20 (grey lines across). This male-specific region was assembled via anchoring of the scaffolds with BAC sequences. Approximately 1.3Mb region was covered by Y-allelic scaffolds. More than 400kb of long repetitive sequences (dotted lines), flank *OGI*. Outer regions of these hyper repetitive sequences contain male-specific sequences (blue bands in M/F rate) and pseudo autosomal region (PAR)-like sequences (orange lines), where M/F rate was less than 70%, and the percentage of repetitive sequences was much lower. **b**, The silent divergence rate (*dS*) between X and Y alleles of the genes located in the PAR-like sequences (orange circles) decreases with distance to *OGI*. Stil, for most of these genes, the *dS* value between the X and Y alleles was larger than the average interspecific *dS* between the X alleles of *D*. *lotus* and *D*. *mespiliformis* (green square and dotted line), *D*. *lotus* and *D*. *virginiana* (blue square and dotted line), and *D*. *lotus* and *D*. *kaki* (red square dotted line). These results suggest that, in these PAR-like sequences, recombination between the X and Y alleles was suppressed before the divergence of *Diospyros* species, or at least predates the divergence between *D*. *lotus* and *D*. *kaki*. *dS* values for genes located in the regions closest to *OGI* are comparable to *dS* values between *OGI* and *MeGI* (gray circle, *dS* = 0.205), which suggest that little or no recombination occurred between these sequences after the establishment of *OGI*. In comparison, dS values between the X and Y alleles of genes located in the recombining region of chromosomes 15 are much lower (while circles on the right).

To investigate the sequence and structure of the sex chromosomes, we undertook the following steps. First, we anchored some pseudo-autosomal scaffolds to chromosome 15, using sex-linked SNPs markers, previously derived from the F1 population described above (S9 Table). On the other hand, the male-specific region of the Y-chromosome, including *OGI*, could not be anchored using SNPs, presumably due to large structural variation between the X and Y chromosome at these locations. Therefore, we anchored 7 Y-chromosomal scaffolds surrounding *OGI* using the end-sequences of BAC clones selected for sequencing based on successive walking starting from *OGI* itself [5]. We then assessed their genomic context by mapping short sequencing reads from male and female individuals of the KK population (Fig 5A) to these scaffolds. The regions flanking *OGI* were male-specific or hyper-repetitive, often including palindrome-like structures (S13 Fig), that are consistent with the sequence context of sex chromosomes in animal [30]. Putative pseudo-autosomal region (PAR)-like sequences, which include both X- and Y-allelic genes, were observed only 200–300 kb from *OGI* (Fig 6A). Such a short Y-specific region is consistent with our previous results [5].

The silent divergence rate (*dS*) between the X and Y alleles of the genes located within the PAR-like sequences decreased with increasing distance from *OGI*, but was always higher than the interspecific *dS* rate between *D*. *lotus* and other *Diospyros* species (Fig 6B), suggesting that recombination was suppressed in the regions flanking OGI before the divergence of some *Diospyros* species. Large structural variation specific to male (or large male-specific region) were frequently observed within the PAR-like sequences (Fig 6A). Contrary to the observation of synteny around *MeGI* and *SiMeGI*, sequence similarity between the regions surrounding *OGI* (Chr. 15) and *MeGI* (Chr. 13), was only observed in the transcriptional regions of *MeGI* and *OGI* (S14 Fig). No significant gene order synteny was observed using SynMap (CoGe). Finally, the phylogenetic relationship between *SiMeGI*, *MeGI*, and the inverted and forward repeats of *OGI* (S15 Fig) suggested that the inverted structure of *OGI* was derived from local inversion after segmental duplication of *MeGI* and (proto)*OGI*. Altogether, these results suggest that *OGI* was not derived from a whole-genome duplication event but from a local segmental duplication event.

### Transitions towards dioecy are associated with duplication events

**Fig 7:**
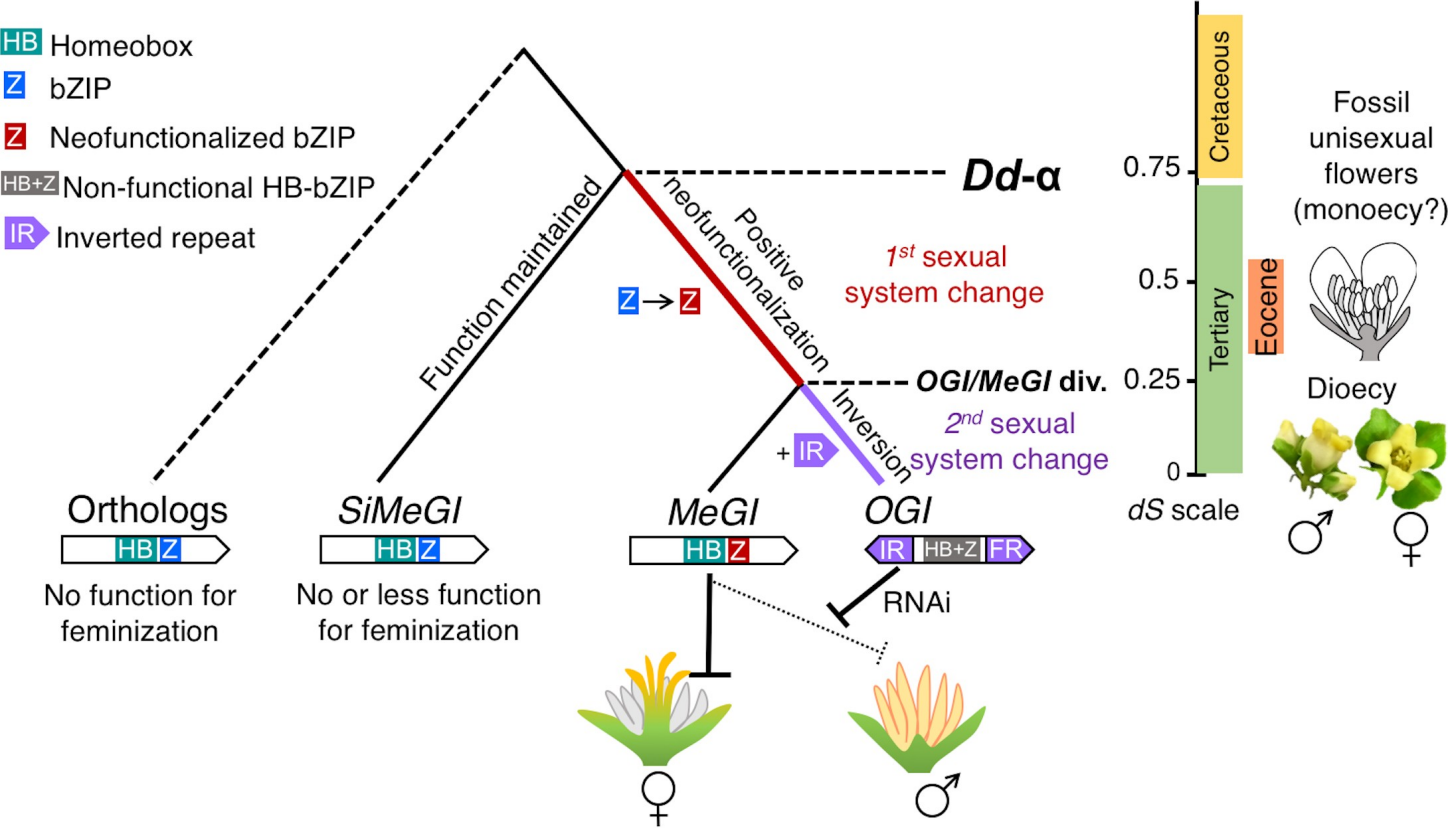
Hypothetical model for the role of duplication events in the evolution sexual systems in *Diospyros*. The *Dd-α* event triggered positive selection on the 5-end and bZIP motifs, resulting in the acquisition of a new role for *MeGI* as repressor of male organs. This was potentially associated with the first switch in sexual system, from hermaprhodistism to monoeocy. The following duplication event, a segmental event, resulted in formation of *OGI*, containing an inverted repeat, which acquired the function of repression of *MeGI* expression via small-RNA production. This potentially triggered the establishment of the XY (heterogametic male) sexual system [5]. On the right, the *dS* scale, corresponds to the evolution of the *MeGI/SiMeGI* families and the observed sexual systems in each era. Based on the study of fossil records, unisexual (male) flowers were present during the Eocene era, which occurred significantly later than the *Dd-α* event (or the K-Pg boundary) [65]. The *OGI/MeGI* divergence and the establishment of the current function of *OGI* is ancestral to diversification within *Diospyros* and consistently, the Y-encoded *OGI* regulates dioecy in the whole *Diospyros* genus [5].

Our results suggest the following working hypothesis for the evolutionary path into dioecy in *Diospyros*. The *Diospyros*-specific WGD event, *Dd-α* resulted in the appearance of *MeGI* and promoted the neofunctionalization of this gene into a dominant suppressor of androecium, as a feminization factor. This was followed by a second, segmental duplication of *MeGI* to derive a Y-encoded *OGI*, which is a dominant repressor of *MeGI* (Fig 7). Interestingly, the information available so far from other dioecious species hints at the possibility that this type of pattern may have played a role in the evolution of dioecy in other species. For example, in the establishment of dioecy in garden asparagus, the Y-encoded putative sex determinant, *SOFF*, is thought to have originated from an *Asparagus*-specific gene duplication event, which was followed by the acquisition of its function as a dominant suppressor of feminization (SuF) [6]. Furthermore, the Y-encoded putative sex determinant in kiwifruit (*Actinidia* spp.), *Shy Girl*, which acts as a dominant suppressor of feminization, also arose via an *Actinidia*-specific duplication event [7], probably one of the *Actinidia*-specific WGD events, *Ad-*α [19]. These parallel paths towards the independent evolution of all three of these sex determinants is probably not coincidental, but consistent with the theoretical framework described above. In flowering plants, transition into separated sexuality requires the appearance and selection of a gain-of-function event in order to acquire a dominant suppressor(s), such as *MeGI*. Whole-genome duplication events provide good opportunities for such a scenario. The concentration of independent paleoplodization events in the K-Pg boundary is consistent with the adaptive evolution of plants against the substantial environmental changes, including mass extinction of their pollinators that took place at the time [11, 31]. A selfing habit engendered by polyploidy would be advantageous, but protracted evolutionary success would be favored by an eventual return to outcrossing. The neofunctionalization of *MeGI* resulting in the acquisition of a lineage-specific new sexual system could be one of these adaptive strategies. This hypothesis is also consistent with the observed wide diversity of sex determination system within plants.

## Materials and methods

### Initial genome sequence assembly

Dormant buds of *D*. *lotus* cv. Kunsenshi-male were burst in the dark for 2-weeks to harvest chlorophyll-starved young leaves (S10 Table). High molecular weight DNA were extracted using the Genome-tip 100/G kit (QIAGEN, Tokyo, Japan), followed by purification using phenol/chloroform extraction. Libraries were size-selected using the Blue Pippin and the following size minimums: 12 kb (14 SMRT cells), 15 kb (34 SMRT cells) and 16 kb (12 SMRT cells). A total of 60 SMRT cells and 54 Gb of PacBio raw data were obtained using the PacBio RSII. Filtered sub-reads were pooled and the longest were retained for assembly, by removing all filtered subreads shorter than 12 kb. This resulted in approximately 32x coverage of the estimated 1 Gb haploid genome size. PacBio reads were assembled using Falcon, producing 3,417 primary contigs and 6,318 alternate contigs. Next, all contigs were assessed for the presence of contaminating sequences by aligning each contig to a custom database using BLASTN+ version 2.2.31+. The custom database contained Kiwifruit psuedomolecule (http://bioinfo.bti.cornell.edu/pub/kiwifruit/Kiwifruit_pseudomolecule.fa.gz), the *A*. *thaliana* chromosomes (http://ftp.arabidopsis.org/home/tair/Genes/TAIR10_genome_release/TAIR10_chromosome_files/), as well as the human draft genome and representative bacterial / archaeal genome databases (pre-formatted blast+ database http://ftp.ncbi.nlm.nih.gov/blast/documents/blastdb.html). Hits to the two contaminant databases were identified and used to remove sequences that were largely contaminant, or to trim those with non-contaminant sequences at least 10kb long. After this step, 3,252 primary and 5,939 alternative contigs were retained. This set of contaminant-free contigs were next polished using quiver (version 2.3.0–140936) and default parameters. After this last step, 3,073 primary and 5,901 alternative contigs remained.

### Illumina library construction and sequencing

#### Genomic libraries

Approximately 1.5 μg of genomic DNA was used for the construction of Illumina genomic libraries; the DNA was fragmented using NEBNext dsDNA Fragmentase (New England BioLabs; NEB) for 40–60 min at 37°C and cleaned using Agencourt AMPure XP (Beckman Coulter Genomics, Tokyo, Japan) for size selection. To select fragments ranging between 300 and 600 bp, 27 μl of AMPure was added to the 63 μl reaction. After a brief incubation at RT, 90 μl of the supernatant was transferred to a new tube and 20 μl water and 30 μl AMPure were added. After a second brief incubation at RT, the supernatant was discarded and the DNA was eluted from the beads in 20 μl of water, as recommended. Next, DNA fragments were subjected to end repair using NEB’s End Repair Module Enzyme Mix, and A-base overhangs were added with Klenow (NEB), as recommended by the manufacturer. A-base addition was followed by AMPure cleanup using 1.8:1 (v/v) AMPure reaction. Barcoded NEXTflex adaptors (Bioo Scientific, Austin, USA) were ligated at room temperature using NEB Quick Ligase (NEB) following the manufacturer’s recommendations. To remove contamination of self-ligated adapter dimers, libraries were size-selected using AMPure in 0.8:1 (v/v) AMPure:reaction volume to select for adapter-ligated DNA fragments at least 400-bp long. Half of the eluted DNA was enriched by PCR reaction using Prime STAR Max (Takara, Tokyo, Japan) at the following PCR conditions: 30 s at 98°C, 10 cycles of 10 s at 98°C, 30 s at 65°C and 30 s at 72°C and a final extension step of 5 min at 72°C. Enriched libraries were purified with AMPure (0.7:1 v/v AMPure to reaction volume), and quality and quantity were assessed using the Agilent BioAnalyzer (Agilent Technologies, Tokyo, Japan) and Qubit fluorometer (Invitrogen, Waltham, USA). Libraries were sequenced using Illumina’s HiSeq 2500 or HiSeq4000 (150-bp paired-end reads).

#### GBS/ddRAD-Seq libraries

Two F1 mapping populations, derived from crosses between two *D*. *lotus*, Kunsenshi-male and Kunsenshi-female, and between two *D*. *lotus*, Kunsenshi-male and Budogaki-female, were employed for ddRAD-Seq [32] and GBS [33] analyses to construct genetic linkage maps. The former and latter mapping populations were named KK (n = 314) and VM (n = 119), respectively. Genomic DNA was extracted from the leaves of each line using the CTAB method. The ddRAD-Seq libraries for KK and VM were constructed using restriction enzymes *PstI* and *Msp*I [34], while the GBS library for KK were prepared using with *Pst*I [33].

#### mRNA libraries

Developing buds and flowers from two *D*. *lotus* individuals, Kunsenshi-male and Kunsenshi-female, were harvested from June to April to cover the annual cycle of leaves/flower development. Total RNA was extracted using the Plant RNA Reagent (Invitrogen) and purified by phenol/chloroform extraction. Five micrograms of total RNA was processed in preparation for Illumina Sequencing, according to a previous report [5]. In brief, mRNA was purified using the Dynabeads mRNA purification kit (Life Technologies, Tokyo, Japan). Next, cDNA was synthesized via random priming using Superscript III (Life Technologies) followed by heat inactivation for 5 min at 65°C. Second-strand cDNA was synthesized using the second-strand buffer (200 mM Tris–HCl, pH 7.0, 22 mM MgCl_2_ and 425 mM KCl), DNA polymerase I (NEB, Ipswich, USA) and RNaseH (NEB) with incubation at 16°C for 2.5 h. Double-stranded cDNA was purified using AMPure with a 0.7:1 (v/v) AMPure to reaction volume ratio. The resulting double-stranded cDNA was subjected to fragmentation and library construction, as described above, for genomic library preparation. Ten cycles of PCR enrichment were performed using the method described above. The constructed libraries were sequenced on Illumina’s HiSeq 4000 sequencer (50-bp single-end reads).

#### DAP-Seq libraries

The DAP genomic DNA libraries were prepared as previously described [25, 28, 35]. Briefly, the Covaris M220 ultrasonicator (with the manufacturer-recommended setting) was used to fragment gDNA to an average size of 200 bp. The resulting fragmented gDNA was ligated to the NEXTflex adaptors (Bioo Scientific, Austin, USA) as described, to make genomic libraries. The full-length *SiMeGI* cDNA was cloned into the pDONR221 vector (Life Technologies) and then transferred to the pIX-Halo using LR clonase II (Life Technologies) to generate pIX-Halo-SiMeGI. pIX-Halo-MeGI has been constructed previously [25]. The N-terminally Halo-tagged *MeGI* and *SiMeGI* were produced using the TNT SP6 Coupled Wheat Germ Extract System (Promega, Fitchburg, WI, USA) and purified with Magne HaloTag beads (Promega). A total of 50 ng DAP gDNA library was incubated with Halo-tagged *MeGI* and *SiMeGI* at room temperature for 1 h.

#### Sequencing

The ddRAD Seq sequences were obtained at the Kazusa DNA Research Institute. The GBS sequences were obtained from the Genomic Diversity Facility (Cornell University). All other Illumina sequencing were conducted at the Vincent J. Coates Genomics Sequencing Laboratory at UC Berkeley, and the raw sequencing reads were processed using custom Python scripts developed in the Comai laboratory and available online (http://comailab.genomecenter.ucdavis.edu/index.php/Barcoded_data_preparation_tools), as previously described. In brief, reads were split based on index information and trimmed for quality (average Phred sequence quality > 20 over a 5 bp sliding window) and adaptor sequence contamination. A read length cut-off of 35 bps was applied to mRNA reads. Sequencing analysis of ddRAD-Seq libraries was performed at the Kazusa DNA Research Institute, and data processing was conducted as described in Shirasawa et al. [34].

### Gene prediction and genome/genes annotation

The RNA-Seq data for gene prediction was obtained from developing buds and flowers from *D*. *lotus* Kunsenshi-male at the following eight time points in 2013 to 2015 (June, July, August, October, January, March, early April, and late April) to cover the annual cycle of leaves/flower development. The RNA-Seq reads were trimmed according to previous reports [5]. The cleaned reads were mapped onto the scaffolds of DLO_r1.1 using TopHat 2.0.14 [36], and the BAM files obtained were used for BRAKER1 1.9 pipeline [37]. In the pipeline, GeneMark-ET 4.32 [38] and Augustus 3.1 [39] were used to construct the training set, and Augustus 3.1 was used for the gene prediction, using the training set. Genes were compared to the UniProtKB (http://www.uniprot.org/uniprot/) and of Araport11 [40] peptide sequences using BLASTP with E-value cutoff of 1E-10. Genes that were similar to those in the databases were categorized as “highly confident” (HC). Analysis of the conservation of the single-copy genes was conducted using BUSCO v1 [41]. Repeat sequences were detected using RepeatScout 1.0.5 [42] and RepeatMasker 4.0.6 (http://www.repeatmasker.org) against the Repbase database [43], according to the method used previously [44]. The HC genes on the primary scaffolds (DLO_r1.1 primary) were compared to the genes of *Actinidia chinensis* (kiwifruit; 39,040 genes [19]), *Vitis vinifera* (grape; 29,927 genes (IGGP 12x.31) [45]), *Solanum lycopersicum* (tomato; 34,789 genes (ITAG 3.10) [12]) and *Arabidopsis thaliana* (27,655 genes (Araport11)) using OrthoMCL 2.0.9. To estimate the divergence time between *D*. *lotus*, *A*. *chinensis*, *V*. *vinifera*, and *A*. *thaliana*, the single copy genes conserved amongst all four species were aligned by MUSCLE 3.8.31 [46]. InDels in the alignment were eliminated using Gblocks 0.91b [47], and the sequences were concatenated by species and used to construct the phylogenetic tree using the Maximum Likelihood method using MEGA 7.0.26 [48] with the Jones-Taylor-Thornton (JTT) model as the substitution model. The divergence time was estimated based on that between *A*. *chinensis* and *V*. *vinifera* (117 MYA) published in TIMETREE (http://www.timetree.org).

### Construction of the persimmon database

The sequence data obtained was released in the form of the PersimmonDB (http://persimmon.kazusa.or.jp). In the database, BLAST searches can be conducted against the scaffolds (DLO_r1.0) and pseudomolecules (DLO_r1.0_pseudomolecules), cds (DLO_r1.1_cds), and pep (DLO_r1.1_pep). Keyword searches are available against the results of the similarity searches against TrEMBL and peptide sequences in Araport11. The genomic and genic sequences, GFF files of the scaffolds and pseudomolecules, and BED files can be downloaded from the database. The scaffolds are also available under accession numbers BEWH01000001-BEWH01008975 (8,975 entries) in DDBJ. The raw sequence data is also available from under accession numbers DRA006168 (Illumina WGS for *D*. *lotus* Kunsenshi-male and female), DRA006169-DRA006176 (ddRAD-Seq/GBS for KK and VM populations), DRA006177 (RNA-Seq for *D*. *lotus* Kunsenshi-male), and DRA006182-DRA006184 (PacBio WGS for *D*. *lotus* Kunsenshi-male) in DDBJ.

### Genetic anchoring of the scaffold using two mapping populations

The sequence reads from the ddRAD-Seq and GBS libraries were mapped onto the primary contigs of the DLO_r1.0 reference sequence using Bowtie 2 (version 2.2.3) [49]. SNP calling was performed using the mpileup command of SAMtools (version 0.1.19) [50] and the view command of BCFtools [50]. High-confidence SNPs were selected using VCFtools (version 0.1.12b) [51] using the following parameters: ≥10× coverage of each sample (—minDP 10); >999 SNP quality value (—minQ 999); ≥0.2 minor allele frequency (—maf 0.2), and <0.5 missing data rate (—max-missing 0.5). Totals of 3,535 and 4,027 high-confident SNPs were obtained in the KK and VM populations, respectively. Genotype information for all lines were prepared for the CP mode of JoinMap (version 4) and classified into groups using the Grouping Module of JoinMap with LOD scores of 4 to 7. Marker order and relative map distances were calculated using its regression-mapping algorithm with the following parameters: Haldane’s mapping function ≤0.35 recombination frequency, and  ≥2.0 LOD score. LPmerge (version 1.5) [52] was used to integrate the linkage maps into a single consensus map. To construct pseudomolecule sequences, scaffolds assigned to the genetic map for the Kunsenshi-male, the cultivar used for the genome sequencing analysis, were ordered and oriented in accordance with marker order if at least two marker loci were mapped on a single scaffold. Otherwise, in the cases of a single marker on a scaffold, the orientation of the sequence was determined as “unknown”.

### Genomic characterization of the male-specific region of the Y-chromosome

For the male-specific regions of the Y-chromosome (MSY), including *OGI*, which was not anchored into Chr. 15 with SNPs markers, we aligned the candidate scaffolds using BAC-end sequences walking from the seed BAC clone that including *OGI* [5]. BAC library construction and screening were described previously [5]. A total of 14 BAC clones were isolated to connect each other, and anchor 7 scaffolds surrounding *OGI*. The genomic reads of 10 male and 10 female individuals from the KK population [5] were mapped to these sequences using BWA with the default mismatch allowance to determine reads coverage, and with no mismatch allowed to define the allele type (X or Y). In this study, >3kb sequences were defined as pseudo-autosomal region (PAR)-like sequences, based on the fact that > 30% of mapped reads were female (excluding repetitive sequences).

To investigate the timing of divergence (or suppression of recombination) between the X and Y alleles of the genes located within the MSY and within the seven anchored scaffolds, X-allelic sequences were reconstructed by mapping the Illumina reads from female individuals from the KK population, to the Y-allelic reference sequences. The X- and Y-allelic sequences were aligned with MAFFT ver. 7 and analyzed with DnaSP 5.1 [53] to detect *dS* values between X-Y alleles. To standardize the estimated timing of divergence, the interspecific *dS* values between the X-alleles in *D*. *lotus* and three *Diospyros* species (*D*. *kaki*, *D*. *virginiana*, and *D*. *mespiliformis*; [5]) were assessed using DnaSP 5.1. The X-Y allelic *dS* were also measured in the recombining regions of the sex chromosomes (Dlo_pri0017F.1 and Dlo_pri0114F.1).

### Comparative genomics

Whole genome-resequencing analysis on the Kunsenshi-male and female individuals were performed as described in Shirasawa et al. (2017) [54]. Paired-end sequences reads were obtained from the male and female lines with Illumina NextSeq, and trimmed and filtered based on quality score using Prinseq [55] and base similarity to adapter sequences, AGATCGGAAGAGC, using fastx_clipper in the FASTX-Toolkit (http://hannonlab.cshl.edu/fastx_toolkit). The resulting reads were mapped on the primary contigs of DLO_r1.0 reference sequence with Bowtie2, and single nucleotide polymorphisms were detected with SAMtools mpileup [50] and filtered with the conditions of sequence depth of ≥10 in each line (—minDP 10) and mapping quality of >200 in each SNP locus (—minQ 200) using VCFtools [51]. The effect of SNPs on gene function were predicted with SnpEff [56] to assign the SNPs to four impact categories, high, moderate, modifier, and low, predifined by SnpEff. Synteny relationship of the genome structures were predicted with PROmer program of Mummer package [57] between *Diospyros* (this study) and *Actinidia* [19], as well as within the *Diospyros lotus* genomic fragments. The results were filtered using delta-filter and default parameters, and options of -i 20 -u 20. The results were visualized using Mummerplot or Circos [58]. Collinearity of homologous genes (<e^-100^ in blastp analysis) was visualized using Strudel [59]. Collinearity of gene order was analyzed and visualized using SynMap and GEvo in CoGe [20] (https://genomevolution.org/coge/).

### Detection of genetic diversity within paralogs

Genes annotated as potential transposable elements by blastn/blastp using the TAIR/nr databases, and potentially repetitive genes which produced >5 homologous genes in the *D*. *lotus* genome (<e^-20^ in blastp), were discarded. *D*. *lotus* gene pairs showing significant sequence similarity (<e^-20^ in blastp), and their orthologs from three species, *Actinidia*, *Solanum* and *Vitis*, which were nested into the same gene family according to OrthoMCL results [60], were subjected to in-codon frame alignment using their protein and nucleotide sequences with Pal2Nal and MAFFT ver. 7 under the L-INS-i model. The resulting alignments were subjected to Mega v.6 to estimate the Jukes and Cantor corrected values of synonymous (*dS*) and non-synonymous (*dN*) substitutions and the index of evolutionary rate (*dN/dS*). The four-fold degenerative sites were extracted from the alignments with PAML (icode = 11), and their pairwise transversion rates (4DTv) were calculated according to previous reports [61]. To estimate the divergence time between the gene pairs, we adopted an estimated rate of 2.81 × 10^−9^ substitutions per synonymous site per year, according to the report in *Actinidia* [62].

### Evolutionary analysis on the paralogs derived from *Dd-α* WGD event

To search for signs of positive selection, aligned nucleotide sequences of each gene pair and an outgroup ortholog, from either the *Actinidia*, *Solunum* or *Vitis* genomes, were subjected to codon-based detection of positive selection test using PAML [63]. The statistical significance of positive selection on branches was evaluated using the likelihood ratio test of the null hypothesis that *dN/dS* = 1. Site-specific positive selection was assessed by Bayes Empirical Bayes analysis. To examine the positively selected sites common across the all three outgroups, in-frame alignments of the *D*. *lotus* gene pairs with the orthologs from all of the *Actinidia*, *Solanum* or *Vitis* genomes were used for the construction of evolutionary topologies using ML method by Mega v. 6, using the general time reversible (+I+G) model. Based on these alignments and topology, the branch- and site-specific positive selection test was performed using PAML, as well.

To define the phylogenetic relationship between the *MeGI*/*SiMeGI*-like orthologs/paralogs in angiosperms, genes showing significant homology (<1e^-10^ in blastp analysis) to a HD-ZIP1 OsHOX4 from *Oryza sativa*, which was previously used as the outgroup gene for the *MeGI* clade [5], were collected from the *Diospyros lotus*, *Solanum lycopersicum*, *Arabidopsis thaliana*, *Oryza sativa*, and *Zea mays* genomes. A total of 174 protein sequences from these genomes, and that of Vrs1 from barley [15] were aligned using MAFFT ver. 7, followed by manual pruning with SeaView. The pruned alignment was subjected to the NJ approach using Mega v. 6, with the JTT model, to construct phylogenetic tree (S6 Fig).

To assess selective pressure on *MeGI* and *SiMeGI*, their alleles from other members of the Ebenaceae family (*Diospyros* and *Euclea* genera), and their orthologs in the *Actinidia*, *Solunum* or *Vitis* genomes were subjected to in-codon frame alignment by MAFFT ver. 7, followed by a ML approach using Mega v. 6, with HKY+G model, to construct an evolutionary topology. The putative ancestral sequences of the *MeGI* and *SiMeGI* origins in the Ebenaceae family, and the sequences in the most recent common ancestor (MRCA) of the order Ericale and of the Asterids, were estimated using Mega. Informative SNPs in the aligned sequences were analyzed by DnaSP 5.1 [53] and used to calculate a series of window-average *dN/dS* values, from the start codon (ATG) in a 150-bp window with a 30-bp step size, until the walking window reached the stop codon.To assess differentiation of expression patterns between the *Dd-α*-derived paralog pairs, we conducted Pearson’s product moment correlation analysis and Fisher’s exact test. Differentiation between the developmental stages of the buds/flowers throughout the annual cycle was examined by the “cor.test” function in R (with “pearson” method), using mRNA-Seq transcriptome data from datapoints (S3 Dataset). Differentiation of expression pattern between male and female flowers was examined for each paralog pair using a 2x2 Fisher’s exact test (“fisher.test” function in R), and using mRNA-Seq transcriptome data from early developing stage and maturing stage, respectively (S3 Dataset).

### Transformation of *MeGI* and *SiMeGI*

Full length sequences of the *MeGI* and *SiMeGI* transcripts were amplified by PCR using PrimeSTAR Max (TaKaRa) from cDNA synthesized from RNA, itself derived from developing flower buds of *D*. *lotus* cv. Kunsenshi-male. The amplicons were cloned into the pGWB2 vector to place the genes under the control of CaMV35S promoter. We constructed pGWB2-*MeGI* and pGWB2-*SiMeGI* using the Gateway system (Invitrogen) and the pENTR/D-TOPO cloning kit and LR clonase. Tobacco plants (*N*. *tabacum*) cv. Petit Havana SR1 were grown *in vitro* under white light with 16-h-light and 8-h-dark cycles at 22°C until transformation. The binary construct was introduced into the *A*. *tumefaciens* strain EHA101. Young petioles and leaves of tobacco plants were transformed by the leaf disk method as previously described [5]. Transgenic plants were selected on Murashige and Skoog medium supplemented with 100 μg/mL kanamycin. Pollen tube germination was assessed 6 h after placing the pollen grains on 15% sucrose/0.005% boric acid/1.0% agarose media at 25°C. The pollen germination ratio was counted as average percentages in batches of 200 pollen grains from the first three flowers.

### RNA *in situ* hybridization

RNA *in situ* hybridization was performed as previously described [64], but with minor modifications. Briefly, bud samples were fixed in FAA (1.8% formaldehyde, 5% acetic acid, 50% ethanol), dehydrated using an ethanol: t-butanol series, and then embedded in paraffin. The embedded tissues were sliced into ca 10-μm sections, and the sections were mounted on FRONTIER coated glass slide (Matsunami Glass Ind., Japan). Paraffin was removed with xylene, and the tissue sections were rehydrated in an ethanol series. The tissue sections were then incubated in a Proteinase K solution (700U/mL Proteinase K, 50mM EDTA, 0.1M Tris-HCl pH 7.5) for 30 min at 37°C, followed by acetylation with acetic anhydride (0.25% acetic anhydride in 0.1 M triethanolamine solution) for 10 min. Full length *MeGI* and *SiMeGI* cDNA sequences were cloned into the pGEM-T Easy vector (Promega, WI, USA) to synthesize the DIG-labelled probes, respectively. Antisense RNA probes were synthesized using the DIG-labeling RNA synthesis kit (Roche, Switzerland), according to the manufacturer’s instruction. The probe solution including RNaseOUT (Thermo Fisher Scientific, Waltham, USA) was applied to the slides and covered with parafilm. Hybridization was performed at 48°C for >16 h. For detection, 0.1% Anti-Digoxigenin-AP Fab fragments (Sigma-Aldrich, St. Louis, USA) was used as the secondary antibody to stain with NBT/BCIP solutions.

## Supporting information

S1 Fig kmer distribution to estimate genome size and the degree of heterozygosity. The distribution of distinct *k*-mers (k  =  17) from the Illumina short reads showed two peaks at multiplicities of 32 and 54. The low and high peaks represent heterozygous and homozygous sequences, respectively. We estimated the genome size to be 877.7 Mb from the higher peak. This estimation almost agreed with the value measured by flow cytometry, 907 Mb, which was calculated from the nuclear DNA content in *D*. *lotus* of 1.85 pg/2 C (Tamura et al., 1998) and an assumption that 1 pg of DNA is equivalent to 980 Mb (Bennett et al., 2000).
(PDF)

S2 Fig Genetic anchoring data (Dlo01-15). Genetic linkage map (left bars) and physical map of Dlo_r1.0 pseudomolecule sequences (right bars). Colors in the genetic map represent density of SNPs per 5 cM, while black, white and gray bars in the physical map indicate the positions of the forward, invert, and unknown directional scaffold sequences integrated in the pseudomolecule, respectively.
(PDF)

S3 Fig Conservation of gene and repetitive sequences across representative plant species. **a,** Amino acid sequences were compared among genes from *D*. *lotus* (40,532 genes; DLO_r1.1 primary), *A*. *chinensis* (39,040 genes (Huang et al., 2013)), *V*. *vinifera* (29,927 genes (IGGP 12x.31) (Jaillon et al., 2009)), *S*. *lycopersicum* (34,789 genes (ITAG 3.10) (The Tomato Genome Consortium, 2012)), and *A*. *thaliana* (27,655 genes (Araport11) (Cheng et al., 2017)) using OrthoMCL v2.0.9 (Li et al., 2003) with default parameters. The numbers of clusters were shown in the intersections of the Venn diagram. **b,** Repetitive sequences were identified by RepeatMasker v4.0.6 (http://www.repeatmasker.org) using Repbase v406 (http://www.girinst.org/repbase/) and RepeatScout v1.0.5 for the genome sequences of *D*. *lotus* (8,974 sequences; DLO_r1.0), *A*. *chinensis* (30 pseudomolecules (Huang et al., 2013)), *S*. *lycopersicum* (13 pseudomolecules (SL3.0) (The Tomato Genome Consortium, 2012)), *Lactuca sativa* (lettuce; 9 pseudomolecules (V8) (Reyes-Chin-Wo et al., 2017)), *V*. *vinifera* (33 chromosomes (IGGP 12x.31) (Jaillon et al., 2009)), *Prunus persica* (peach; 8 pseudomolecules (v2.0.a1) (Verde et al., 2013), *Carica papaya* (papaya; 5,901 scaffold sequences (ASGPBv0.4) (Ming et al., 2008)), and *A*. *thaliana* (5 chromosomes, chloroplast and mitochondria genomes (TAIR10)). The percentage of repetitive sequences against the total length of the genome sequence were calculated for each of the result of RepeatMasker and RepeatScout and compared among the plant species.
(PDF)

S4 Fig Genome-wide syntenic analysis. **a**, Synteny analysis based on gene order using CoGe SynMap, and using only the gene pairs identified as putatively derived from the *Dd-α*, with *dS* values between 0.5 and 0.9. The masked syntenic blocks (*dS* < 0.5 or *dS* > 0.9) are shown in gray. Syntenic blocks were detected with (B)lastz using default parameters. Long syntenic blocks with *dS* = 0.5–0.9 were conserved throughout the genome, and were consistent with the main duplicated blocks shown in Fig 1. Panel b is more detailed view of the region highlighted by red rectangles and annotated Syn I. **b**, Example of a large syntenic CDS blocks of genes with *dS* values between 0.5 and 0.9, between chromosome 1 and 2.
(PDF)

S5 Fig Gene duplication patterns following the *Dd-α* event. **a,** Heat map for the numbers of genes derived from *Dd-α*, shared between two chromosomes. For instance, Dlo01 shared many paralogs with Dlo02 and Dlo12, while Dlo02 and Dlo12 shared few paralogs with each other. The patterns of such affinities between the chromosomes suggests a paleotetraploidization event. **b,** Syntenic relationship between the putatively *Dd-α*-derived paralogous genes within the *Diospyros* genome.
(PDF)

S6 Fig Genome-wide synteny between *Diospyros* and *Actinidia*. Dot plots of the syntenic genomic regions between *Diospyros* and *Actinidia*. As represented in the orange box, a single genomic segment from *Actinidia* corresponds to two syntenic *Diospyros* genome regions which are derived from the *Dd-α*. In the orange box, the middle regions of Dlo01 and Dlo02, and Dlo03 and Dlo06 are duplicated regions via *Dd-α* (see Fig 1 and S4 Fig). On the other hand, as represented in the green box, a genomic segment from *Diospyros* corresponds to at maximum four syntenic *Actinidia* genome regions which are derived from the double *Actinidia*-specific whole-genome duplication events (Ad-α and Ad-β) (Huang et al., 2013). These results indicate that, in the evolution of the order Ericales, *Dd-α* and Ad-α/β occurred independently in the *Diospyros* and *Actinidia* ancestral genomes, respectively.
(PDF)

S7 Fig Physical relationship of the syntenic segments between the regions surrounding *MeGI* and *SiMeGI*. Syntenic relationships in the *MeGI* and *SiMeGI* surrounding regions, using GEvo (CoGe). The high-scoring segment pairs (HSP) detected are shown here connected with red lines. They correspond to gene pairs with *dS* values ranging between 0.5 and 0.9, or regions flanking genes, in the Dlo_pri0025F and Dlo_pri0799F genomic contigs.
(PDF)

S8 Fig Phylogenetic tree of the angiosperm HD-ZIP1 family. Phylogeny of the HD-Zip1 type homeodomain genes in representative angiosperm genomes (*Solanum lycopersicum*, *Oryza sativa*, *Zea mays*, and *Arabidopsis thaliana*) and the *D*. *lotus* genome. The 175 HD-ZIP1 genes clustered into 8 major clades (Clade I-VIII), which each included at least one homolog from all 5 species used in this study (see Materials and Methods). It is important to note that the root branches of clades VI and VII were not statistically significant though (32/100 and 28/100, respectively). *MeGI* and *SiMeGI* from persimmon, the three closest orthologs from Arabidopsis, *Vrs1* from barley and *LOC_Os07g39320* from rice were all nested within clade IV (colored in light green). No other persimmon paralog nested into clade IV (bootstrap = 100/100). This suggested that the other persimmon paralogs had diverged from *MeGI* and *SiMeGI* before the divergence of the angiosperms (or the time of divergence between monocots and dicots).
(PDF)

S9 Fig Overexpression of *MeGI* and *SiMeGI* under the control of CaMV35S promoter in *N. tabacum*. **a-c**, 1-week old transgenic lines. The *MeGI*-induced lines (**a**) frequently showed clear irregularities in development, in comparison to the *SiMeGI* lines (**b**) or empty cassette-induced lines (**c**). **d**, Comparison of 4-weeks old transgenic plants. The *MeGI*-induced lines (center) uniformly showed more severe growth inhibition, than the *SiMeGI*-induced lines (left). **e**, close-up picture of the *MeGI*-induced line corresponding to the individual marked with an asterisk in the panel (**d**). The leaves showed irregular shapes with significantly less veins. **f**, comparison of the appearance of 15-weeks old transgenic lines. The *MeGI*-induced line (left) exhibited dwarfism, but the total number of leaves were comparable to the control plants (right), while the internode lengths were shorter than the control, as shown in the panel “**g**”. **h**, Differentiation of the leave shapes and structures in the control (left) and the *MeGI*-induced line (right). The *MeGI*-induced lines produced narrow and serrated leaves. Bars indicate 10mm for a-c, and e; 50mm for d, f, g, and h.
(PDF)

S10 Fig Overexpression of *MeGI* and *SiMeGI* under the control of CaMV35S promoter in *A. thaliana*. **a**, Dissection of the control Arabidopsis plant transformed with an empty cassette. an: anther, pe: petal, sg: stigma. **b-e**, p35S-*MeGI* transgenic lines. Dissected flowers show rudimental anthers (ra) (**b-c**). Approximately half of the transgenic plants are semi-dwarf (semi-dwf) (**d**) or complete dwarf (**e**). They also frequently showed leaf serration, which is consistent with our previous analysis of the p35S-*MeGI* induced Arabidopsis plants (Akagi et al., 2014). **f-i**, p35S-*SiMeGI* transgenic lines. The transgenic plants occasionally showed rudimental anthers similar to the *MeGI*-induced lines (**f-g**). A part of the *SiMeGI*-induced lines showed semi-dwarfism (**h**), but full dwarfism was never observed in the 63 transgenic lines. Over 95% of the *SiMeGI*-induced lines were hermaphroditic, where the numbers of stamens are properly maintained (**i**), in contrast to the *MeGI*-induced lines (Akagi et al., 2014). Bars indicate 1mm for a, b, f, and i; 0.1mm for c and g; 10mm for d-e and h. j, Distribution of the number of female (fe) and hermaphrodite (herm) individuals in the p35-*MeGI* (green), p35S-*SiMeGI* (yellow), and p35S-empty (cont; gray) transgenic lines. k, Distribution of the number of complete dwarf (dwf), semi-dwarf (semi-dwf) and normal individuals in the p35-*MeGI*, p35S-*SiMeGI*, and p35S-empty (cont) transgenic lines.
(PDF)

S11 Fig *in situ* RNA hybridization. RNA *in situ* hybridization in developing buds and flower primordia, using *MeGI* (**a-c**) and *SiMeGI* (**d-f**) sequences as probes. In the cross section of the developing buds (**a** and **d**), the *MeGI* signal is strong in flower buds only (fb) (**a**), while *SiMeGI* showed significant signal in the pith (Pi) and young leaves (ly), as well as in flower buds (**d**). This is consistent with our expression analyses using laser capture micro-dissected (LCM) samples (S12 Fig). In the longitudinal sections of the developing buds (**b** and **e**), both *MeGI* and *SiMeGI* signals are confined to the meristematic region, especially in the shoot apical meristems (sam). At a later developing stage (**c** and **f**), flower primordia (fp) and bract (br) showed substantial signals of both *MeGI* (**c**) and *SiMeGI* (**f**). Bars indicate 50μm.
(PDF)

S12 Fig Expression analysis of *MeGI/SiMeGI* in laser capture microdissection (LCM). Longitudinal (**a**) and cross (**b**) sections of buds from *D*. *lotus*, Kunsenshi-male, and the target of the LCM. We targeted flower buds (red), young leaf or leaf buds (blue), and pith or cambium (green). **c**, the section after laser captions. **d**, qRT-PCR analysis to detect relative expression of the *MeGI* and *SiMeGI* among the organs, at early developmental stages (Jun-Jul) when the flower primordia form. Consistent with the results of the in situ hybridization (S11 Fig), *MeGI* expression was much stronger in flower buds than in pith or young leaves, while the difference in expression levels between the three organs was less drastic for *SiMeGI*. For both graphs, the expression level in flower buds was defined as “1”. **e**, comparison of the expression level of *MeGI* and *SiMeGI* in the developing flower buds. Illumina mRNA-Seq analysis was conducted on the LCM samples to detect RPKM values of *MeGI* and *SiMeGI*. *SiMeGI* was expressed higher than or comparative to the *MeGI*, in the developing flower buds. Notwithstanding, the reduction in *MeGI* expression in this stage affect the flower sexuality and the inflorescent structure (Akagi et al., 2014). **f**, relative expression of the *MeGI* and *SiMeGI* in different organs, during dormancy stage (Dec) when the development of flower primordia halt. Flower buds showed no significant expression of either *MeGI* or *SiMeGI*.
(PDF)

S13 Fig Structure of the sequence surrounding *OGI*. Self-syntenic collinearity was detected in the Y-chromosomal region flanking *OGI* (Scaffold Dlo_pri1021F.1). Inverted and forwarded repeat blocks were frequently conserved, of which some act for small-RNA productions. Forward and reverse syntenic strands are shown in red and blue, respectively.
(PDF)

S14 Fig Syntenic analysis between the regions surrounding *OGI* and *MeGI*. Scaffold Dlo_pri0799F.1, which includes *MeGI* on Chr. 13, and scaffold Dlo_pri1021F.1, which includes *OGI* on Chr. 15, were aligned to each other to detect syntenic blocks. Both scaffolds are continuous and devoid of sequence gaps. Segmental collinearity was not detected between these regions, except for the transcriptional regions of *OGI* and *MeGI*.
(PDF)

S15 Fig Phylogenetic analysis of the establishment of *MeGI*, proto *OGI*, and the inverted repeat of *OGI*. The nucleotide sequences of *SiMeGI*, *MeGI*, and the forward and inverted repeats of *OGI* were aligned to each other to estimate their relative timing of establishment. Our results indicated that the gene duplication event that generated *MeGI* and proto *OGI* (pink outlined circle) postdated the *Dd-α* whole-genome duplication event (or concurrent segmental duplication) which produced the *MeGI* and *SiMeGI* pair (gray filled circle). This result is well supported (bootstrap 99/100). Later, the inverted repeat of *OGI* was probably generated by local inversion of the proto *OGI* (blue outlined circle, bootstrap = 92/100). FR: forward repeat, IR: inverted repeat. Achn210611 from *Actinidia chinensis* was used as the outgroup. Divergence of Achn210611 from the *MeGI/SiMeGI* family predated the *Dd-α* (Fig 4B). This topology was constructed with MEGA v6 using the maximum likelihood method (GTR+I+G, gamma = 3, complete deletion).
(PDF)

S1 Table Summary statistics for the initial genome assembly of *D. lotus* cv. Kunsenshi-male. (PDF)

S2 Table Number of SNPs and length of genetic linkage maps in *D. lotus*. Genetic maps for the four parental lines of the two mapping populations (KK and VM), were built using the pseudo-test cross method. All linkage groups were anchored to the 15 chromosomes of the *D*. *lotus* draft genome assembly. The sex-determinant locus was mapped to linkage group 15, suggesting the Dlo15 is the sex chromosome. More detailed information about the maps and SNPs are available from the Persimmon Genome Database (http://persimmon.kazusa.or.jp)
(PDF)

S3 Table Number of annotated SNPs and indels between the female and male lines of *D. lotus* 'Kunsenshi'. SNPs and indels were identified from whole-genome resequencing analysis of female and male lines of *D*. *lotus*, and functionally annotated and classified into four categories predefined by SnpEff (Cingolani et al., 2012): high (e.g. nonsense mutations and frameshift mutations)-, moderate (e.g. missense mutations)-, modifier (e.g. intron and intergenic mutations)- and low-impact (e.g. synonymous mutations) mutations (see http://snpeff.sourceforge.net for details). Further details about these SNPs and Indels are available from the Persimmon Genome Database (http://persimmon.kazusa.or.jp)
(PDF)

S4 Table Comparison of the repeat sequences in representative eudicot genomes. Repetitive sequences amounted for 630.2 Mb (66.6%) of the total length of the final genome assembly. Unique repeats were abundant in the *D*. *lotus* genomes, constituting 49.8% of all repeats. Of the known types of repeats, Class I LTR elements were observed most frequently (11.2%).
(PDF)

S5 Table Phenotypic characterization of the p35S*-MeGI N. tabacum* transformed lines. (PDF)

S6 Table Phenotypic characterization of the p35S*-SiMeGI N. tabacum* transformed lines. (PDF)

S7 Table Phenotypic characterization of the p35S*-MeGI A. thaliana* transformed lines. (PDF)

S8 Table Phenotypic characterization of the p35S*-SiMeGI A. thaliana* transformed lines. (PDF)

S9 Table Anchoring of chromosome 15 using sex-linked (Y-allelic) SNPs markers. (PDF)

S10 Table Plant materials. (PDF)

S1 Dataset Table of predicted gene locations based on BLAST results. (XLSX)

S2 Dataset Result of the Pearson correlation test for correlation between the expression patterns of paralog pairs. For each paralog pair, the r and t-test p-values are indicated, in addition to the RPKM values at each of the 16 expression time points selected.
(XLSX)

S3 Dataset Results of the Fisher Exact test of the relationship between expression of the two paralogs in each paralog pair in male and female developing flowers. For each paralog pair, the ratio of male to female expression is indicated as well as the result of the Fisher 2 x 2 Exact test p-value.
(XLSX)

## References

[1] Renner SS. The relative and absolute frequencies of angiosperm sexual systems: dioecy, monoecy, gynodioecy, and an up-dated online database. Amer J Bot. 2014; 101: 1588–1596. 25326608 10.3732/ajb.1400196

[2] Liu Z, Moore PH, Ma H, Ackerman CM, Ragiba M, Yu Q, et al A primitive Y chromosome in papaya marks incipient sex chromosome evolution. Nature. 2004; 427: 348–352. 10.1038/nature02228 14737167

[3] Wang J, Na J-K, Yu Q, Gschwend AR, Han J, Zeng F, et al Sequencing papaya X and Yh chromosomes reveals molecular basis of incipient sex chromosome evolution. Proc Nat Acad Sci USA. 2012; 109: 13710–13715. 10.1073/pnas.1207833109 22869747 PMC3427123

[4] Kazama Y, Ishii K, Aonuma W, Ikeda T, Kawamoto H, et al A new physical mapping approach refines the sex-determining gene positions on the *Silene latifolia* Y-chromosome. Sci Rep. 2016; 6: 18917 10.1038/srep18917 26742857 PMC4705512

[5] Akagi T, Henry IM, Tao R, Comai L. A Y-chromosome-encoded small RNA acts as a sex determinant in persimmons. Science. 2014; 346: 646–650. 10.1126/science.1257225 25359977

[6] Harkess A, Zhou J, Xu C, Bowers JE, Van der Hulst R, et al The asparagus genome sheds light on the origin and evolution of a young Y chromosome. Nat Comm. 2017; 8: 1279. 10.1038/s41467-017-01064-8 PMC5665984 29093472

[7] Akagi T, Henry IM, Ohtani H, Morimoto T, Beppu K, Kataoka I, et al A Y-encoded suppressor of feminization arose via lineage-specific duplication of a cytokinin response regulator in kiwifruit. Plant Cell. 2018; 30: 780–795. 10.1105/tpc.17.00787 29626069 PMC5969274

[8] Charlesworth B, Charlesworth D. A model for the evolution of dioecy and gynodioecy. Amer Nat. 1978; 112: 975–997.

[9] Charlesworth D, Charlesworth B. Population genetics of partial male-sterility and the evolution of monoecy and dioecy. Heredity. 1978; 41: 137–153.

[10] Flagel LE, Wendel JF. Gene duplication and evolutionary novelty in plants. New Phytol. 2009; 183: 557–564. 10.1111/j.1469-8137.2009.02923.x 19555435

[11] Van de Peer Y, Mizrachi E, Marchal K. The evolutionary significance of polyploidy. Nat Rev Genet. 2017; 18: 411–424. 10.1038/nrg.2017.26 28502977

[12] The Tomato Genome Consortium. The tomato genome sequence provides insights into fleshy fruit evolution. Nature. 2012; 485: 635–641. 10.1038/nature11119 22660326 PMC3378239

[13] Olsen JL, Rouzé P, Verhelst B, Lin YC, Bayer T, et al The genome of the seagrass *Zostera marina* reveals angiosperm adaptation to the sea. Nature. 2016; 530: 331–335. 10.1038/nature16548 26814964

[14] Fraser LG, Tsang GK, Datson PM, De Silva HN, Harvey CF, Gill GP, et al A gene-rich linkage map in the dioecious species *Actinidia chinensis* (kiwifruit) reveals putative X/Y sex-determining chromosomes. BMC Genom. 2009; 10: 102. 10.1186/1471-2164-10-102 PMC2661093 19284545

[15] Komatsuda T, Pourkheirandish M, He C, Azhaguvel P, Kanamori H, Perovic D, et al Six-rowed barley originated from a mutation in a homeodomain-leucine zipper I-class homeobox gene. Proc Nat Acad Sci USA. 2007; 104: 1424–1429. 10.1073/pnas.0608580104 17220272 PMC1783110

[16] Whipple CJ, Kebrom TH, Weber AL, Yang F, Hall D, Meeley R, *grassy tillers1* promotes apical dominance in maize and responds to shade signals in the grasses. Proc Nat Acad Sci USA. 2011; 108: E506–E512. 10.1073/pnas.1102819108 21808030 PMC3158142

[17] Sakuma S, Pourkheirandish M, Hensel G, Kumlehn J, Stein N, Tagiri A, et al Divergence of expression pattern contributed to neofunctionalization of duplicated HD-Zip I transcription factor in barley. New Phytol. 2013; 197: 939–948. 10.1111/nph.12068 23293955

[18] Tamura M, Tao R, Yonemori K, Ustunomiya N, Sugiura A. Ploidy level and genome size of several *Diospyros* species. J Jpn Soc Hortic Sci. 1998; 67: 306–312.

[19] Huang S, Ding J, Deng D, Tang W, Sun H, Liu D, et al Draft genome of the kiwifruit *Actinidia chinensis*. Nat Comm. 2013; 4: 2640. 10.1038/ncomms3640 PMC4089393 24136039

[20] Lyons E, Pedersen B, Kane J, Alam M, Ming R, Tang H, et al Finding and comparing syntenic regions among Arabidopsis and the outgroups papaya, poplar, and grape: CoGe with Rosids. Plant Physiol. 2008; 148: 1772–1781. 10.1104/pp.108.124867 18952863 PMC2593677

[21] Iorizzo M, Ellison S, Senalik D, Zeng P, Satapoomin P, et al A high-quality carrot genome assembly provides new insights into carotenoid accumulation and asterid genome evolution. Nat Genet. 2016; 48: 657–666. 10.1038/ng.3565 27158781

[22] Reyes-Chin-Wo S, Wang Z, Yang X, Kozik A, Arikit S, Song C, et al Genome assembly with in vitro proximity ligation data and whole-genome triplication in lettuce. Nat Comm. 2017; 8: 14953. 10.1038/ncomms14953 PMC5394340 28401891

[23] Vanneste K, Baele G, Maere S, Van De Peer Y. Analysis of 41 plant genomes supports a wave of successful genome duplications in association with the Cretaceous–Paleogene boundary. Genome Res. 2014; 24: 1334–1347. 10.1101/gr.168997.113 24835588 PMC4120086

[24] Roulin A, Auer PL, Libault M, Schlueter J, Farmer A, May G, et al The fate of duplicated genes in a polyploid plant genome. Plant J. 2013; 73: 143–153. 10.1111/tpj.12026 22974547

[25] Yang HW, Akagi T, Kawakatsu T, Tao R. Gene networks orchestrated by *MeGI*: a single‐factor mechanism underlying sex determination in persimmon. Plant J. 2019; 98: 97–111. 10.1111/tpj.14202 30556936 PMC6850717

[26] Ariel FD, Manavella PA, Dezar CA, Chan RL. The true story of the HD-Zip family. Trends Plant Sci. 2007; 12: 419–426. 10.1016/j.tplants.2007.08.003 17698401

[27] Sakuma S, Salomon B, Komatsuda T. The domestication syndrome genes responsible for the major changes in plant form in the Triticeae crops. Plant Cell Physiol. 2011; 52: 738–749. 10.1093/pcp/pcr025 21389058 PMC3093126

[28] Bartlett A., O'Malley R.C., Huang S.C., Galli M., Nery J.R., Gallavotti A. et al Mapping genome-wide transcription-factor binding sites using DAP-seq. Nat Protoc. 2017; 1659–1672. 10.1038/nprot.2017.055 28726847 PMC5576341

[29] Khan A, Fornes O, Stigliani A, Gheorghe M, Castro-Mondragon JA, van der Lee R, et al JASPAR 2018: update of the open-access database of transcription factor binding profiles and its web framework. Nucleic Acids Res. 2018; D260–D266. 10.1093/nar/gkx1126 29140473 PMC5753243

[30] Bachtrog D, Mank JE, Peichel CL, Kirkpatrick M, Otto SP, Ashman T-L, et al Sex determination: why so many ways of doing it? PLoS Biol. 2014; 10.1371/journal.pbio.1001899 PMC4077654 24983465

[31] Wilf P, Labandeira CC, Johnson KR, Ellis B. Decoupled plant and insect diversity after the end-Cretaceous extinction. Science. 2006; 313: 1112–1115. 10.1126/science.1129569 16931760

[32] Peterson BK, Weber JN, Kay EH, Fisher HS, Hoekstra HE. Double digest RADseq: an inexpensive method for de novo SNP discovery and genotyping in model and non-model species. PLoS ONE. 2012; 7: e37135 10.1371/journal.pone.0037135 22675423 PMC3365034

[33] Elshire RJ, Glaubitz JC, Sun Q, Poland JA, Kawamoto K, Buckler ES, et al A robust, simple genotyping-by-sequencing (GBS) approach for high diversity species. PLoS ONE. 2011; 6: e19379 10.1371/journal.pone.0019379 21573248 PMC3087801

[34] Shirasawa K, Hirakawa H, Isobe S. Analytical workflow of double-digest restriction site-associated DNA sequencing based on empirical and *in silico* optimization in tomato. DNA Res. 2016; 23: 145–153. 10.1093/dnares/dsw004 26932983 PMC4833422

[35] O'Malley RC, Huang SC, Song L, Lewsey MG, Bartlett A, Nery JR, et al Cistrome and epicistrome features shape the regulatory DNA landscape. Cell. 2016; 165: 1280–1292. 10.1016/j.cell.2016.04.038 27203113 PMC4907330

[36] Trapnell C, Pachter L, Salzberg SL. TopHat: discovering splice junctions with RNA-Seq. Bioinformatics. 2009; 25: 1105–1111. 10.1093/bioinformatics/btp120 19289445 PMC2672628

[37] Hoff KL, Lange S, Lomsadze A, Morodovsky M, Stanke M. BRAKER1: Unsupervised RNA-Seq-based genome annotation with GeneMark-ET and AUGUSTUS. Bioinformatics. 2016; 32: 767–769. 10.1093/bioinformatics/btv661 26559507 PMC6078167

[38] Lomsadze A, Gemayel K, Tang S, Borodovsky M. Modeling leaderless transcription and atypical genes results in more accurate gene prediction in prokaryotes. Genome Res. 2018; 28: 1079–1089. 10.1101/gr.230615.117 29773659 PMC6028130

[39] Stanke M, Waack S. Gene prediction with a Hidden-Markov model and a new intron submodel. Bioinformatics. 2003; 19: 215–225. 10.1093/bioinformatics/btg1080 14534192

[40] Krishnakumar V, Hanlon MR, Contrino S, Ferlanti ES, Karamycheva S, Kim M, et al Araport: the Arabidopsis information portal. Nucl Acids Res. 2015; 43: D1003–1009. 10.1093/nar/gku1200 25414324 PMC4383980

[41] Simão FA, Waterhouse RM, Ioannidis P, Kriventseva EV, Zdobnob EM. BUSCO: assessing genome assembly and annotation completeness with single-copy orthologs. Bioinformatics 2015; 31: 3210–3212. 10.1093/bioinformatics/btv351 26059717

[42] Price AL, Jones NC, Pevzner PA. De novo identification of repeat families in large genomes. Bioinformatics. 2005; 21: Suppl 1 i351–358. 15961478 10.1093/bioinformatics/bti1018

[43] Bao W, Kojima KK, Kohany O. Update, a database of repetitive elements in eukaryotic genomes. Mobile DNA. 2015; 6: 11 10.1186/s13100-015-0041-9 26045719 PMC4455052

[44] Hirakawa H, Shirasawa K, Miyatake K, Nunome T, Negoro S, Ohyama A, et al Draft genome sequence of eggplant (*Solanum melongena* L.): the representative solanum species indigenous to the old world. DNA Res. 2014; 21: 649–660. 10.1093/dnares/dsu027 25233906 PMC4263298

[45] Jaillon O, Aury J-M, Noel B, Policriti A, Clepet C, et al The grapevine genome sequence suggests ancestral hexaploidization in major angiosperm phyla. Nature. 2009; 449: 463–467. 10.1038/nature06148 17721507

[46] Edgar RC. MUSCLE: a multiple sequence alignment method with reduced time and space complexity. BMC Bioinfo. 2004; 5: 113. 10.1186/1471-2105-5-113 PMC517706 15318951

[47] Castresana J. Selection of conserved blocks from multiple alignments for their use in phylogenetic analysis. Mol Biol Evol. 2000; 17: 540–552. 10.1093/oxfordjournals.molbev.a026334 10742046

[48] Tamura K, Stecher G, Peterson D, Filipski A, Kumar S. MEGA6: Molecular Evolutionary Genetics Analysis Version 6.0. Mol Biol Evol. 2013; 30: 2725–2729. 10.1093/molbev/mst197 24132122 PMC3840312

[49] Langmead B, Salzberg SL. Fast gapped-read alignment with Bowtie 2. Nat Methods. 2012; 9: 357–359. 10.1038/nmeth.1923 22388286 PMC3322381

[50] Li H, Handsaker B, Wysoker A, Fennell T, Ruan J, Homer N, et al The Sequence Alignment/Map format and SAMtools. Bioinformatics. 2009; 25: 2078–2079. 10.1093/bioinformatics/btp352 19505943 PMC2723002

[51] Danecek P, Auton A, Abecasis G, Albers CA, Banks E, DePistro MA, et al The variant call format and VCFtools, Bioinformatics. 2011; 27: 2156–2158. 10.1093/bioinformatics/btr330 21653522 PMC3137218

[52] Endelman JB, Plomion C. LPmerge: an R package for merging genetic maps by linear programming. Bioinformatics. 2014; 30: 1623–1624. 10.1093/bioinformatics/btu091 24532720

[53] Librado P, Rozas J. DnaSP v5: a software for comprehensive analysis of DNA polymorphism data. Bioinformatics. 2009; 25: 1451–1452. 10.1093/bioinformatics/btp187 19346325

[54] Shirasawa K, Isuzugawa K, Ikenaga M, Saito Y, Yamamoto T, Hirakawa H, et al The genome sequence of sweet cherry (*Prunus avium*) for use in genomics-assisted breeding. DNA Res. 2017; 24: 499–508. 10.1093/dnares/dsx020 28541388 PMC5737369

[55] Schmieder R, Edwards R. Quality control and preprocessing of metagenomic datasets, Bioinformatics. 2011; 27: 863–864. 10.1093/bioinformatics/btr026 21278185 PMC3051327

[56] Cingolani P, Platts A, Wang LL, Coon M, Nguyen T, Wang L, et al A program for annotating and predicting the effects of single nucleotide polymorphisms, SnpEff: SNPs in the genome of *Drosophila melanogaster* strain *w*^1118^; *iso*-2; *iso*-3. Fly. 2012; 6: 80–92. 10.4161/fly.19695 22728672 PMC3679285

[57] Delcher AL, Phillippy A, Carlton J, Salzberg SL. Fast algorithms for large-scale genome alignment and comparison. Nucl Acids Res. 2002; 30: 2478–83. 10.1093/nar/30.11.2478 12034836 PMC117189

[58] Krzywinski M, Schein J, Birol İ, Connors J, Gascoyne R, Horsman D, et al Circos: an information aesthetic for comparative genomics. Genome Res. 2009; 19: 1639–1645. 10.1101/gr.092759.109 19541911 PMC2752132

[59] Bayer M, Milne I, Stephen G, Shaw P, Cardle L, Wright F, et al Comparative visualization of genetic and physical maps with Strudel. Bioinformatics. 2011; 27: 1307–1308. 10.1093/bioinformatics/btr111 21372085 PMC3077070

[60] Li L, Stoeckert CJ, Roos DS. OrthoMCL: Identification of ortholog groups for eukaryotic genomes. Genome Res. 2003; 13: 2178–2189. 10.1101/gr.1224503 12952885 PMC403725

[61] Tuskan GA, Difazio S, Jansson S, Bohlmann J, et al The genome of black cottonwood, *Populus trichocarpa* (Torr. & Gray). Science. 2006; 313: 1596–1604. 10.1126/science.1128691 16973872

[62] Shi T, Huang H, Barker MS. Ancient genome duplications during the evolution of kiwifruit (*Actinidia*) and related Ericales. Annals Bot. 2010; 106: 497–504. 10.1093/aob/mcq129 PMC2924827 20576738

[63] Yang Z. PAML: a program package for phylogenetic analysis by maximum likelihood. Computer Appl Biosci. 1997; 13: 555–556. 10.1093/bioinformatics/13.5.555 9367129

[64] Esumi T, Tao R, Yonemori K. Relationship between floral development and transcription levels of *LEAFY* and *TERMINAL FLOWER 1* homologs in Japanese pear (*Pyrus pyrifolia* Nakai) and quince (*Cydonia oblonga* Mill.). J Jpn Soc Hortic Sci. 2007; 76: 294–304.

[65] Christophel DC, Basinger JF. Earliest floral evidence for the Ebenaceae in Australia. Nature. 1982; 296: 439–441.

