# Correction: The persimmon genome reveals clues to the evolution of a lineage-specific sex determination system in plants

**DOI:** 10.1371/journal.pgen.1008845

**Published:** 2020-05-26

**Authors:** Takashi Akagi, Kenta Shirasawa, Hideki Nagasaki, Hideki Hirakawa, Ryutaro Tao, Luca Comai, Isabelle M. Henry

The images for Figs 5 and 6 are incorrectly switched. The image that appears as Fig 5 should be Fig 6, and the image that appears as Fig 6 should be Fig 5. The figure captions appear in the correct order. Please see the correct Figs 5 and 6 here.

**Fig 5:**
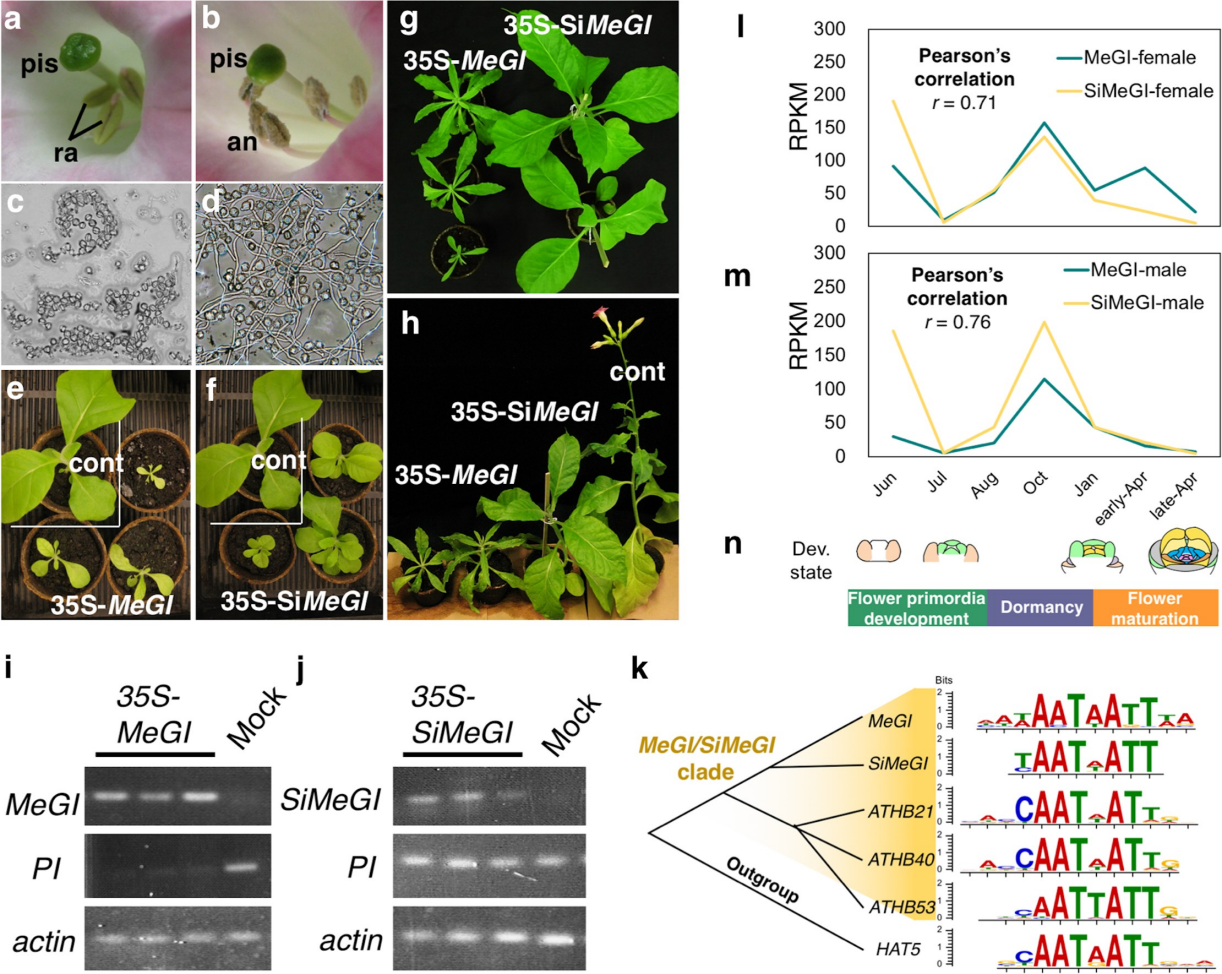
Functional differentiation between *MeGI* and *SiMeGI*. **a-h**, *N*. *tabacum* transgenic lines expressing either of *MeGI* or *SiMeGI* under the control of the 35S promoter. The lines expressing *MeGI* (**a-c**) showed rudimental anthers (**a**) which did not produce functional pollen grains (**b**), and severe dwarfism with chlorophyll starvation and narrow leaves (**c**, see S7 Fig for the detail). The lines expressing *SiMeGI* (**d-f**) developed regular anthers (**d**) which produced fertile pollen (**e**), and showed moderate dwarfism (**f**). pis: pistil, ra: rudimental anthers, an: anthers. **g-h**, Both *MeGI*- and *SiMeGI*-overexpressing lines were phenotypically different from the control plants transformed with empty vectors (cont), but the *MeGI*-expressing lines exhibited more severe departure from the WT controls for specific traits, such as leaves width (see S9 Fig). Bars indicate 5mm for a and d, 50mm for c, f, g, and h. **i-j,** expression patterns of *MeGI*, *SiMeGI*, and *PI*, with *actin* as a positive control, in the transgenic lines transformed with CaMV35S-*MeGI* (i) and CaMV35S-*SiMeGI* (j). **k**, DNA motifs identified as preferentially bound to following transcription factors all nested within the *MeGI/SiMeGI* clade: *MeGI* [25], *SiMeGI* (our experiments), and three Arabidopsis HD-ZIP1 genes [29], using DAP-Seq analyses (see Methods). **l-n**, expression patterns of *MeGI* and *SiMeGI* in buds and flower primordia were highly correlated (Pearson’s *r* > 0.7). Expression levels in female (**l**) and male (**m**) are expressed as RPKM values. **n**, Developmental stages.

**Fig 6:**
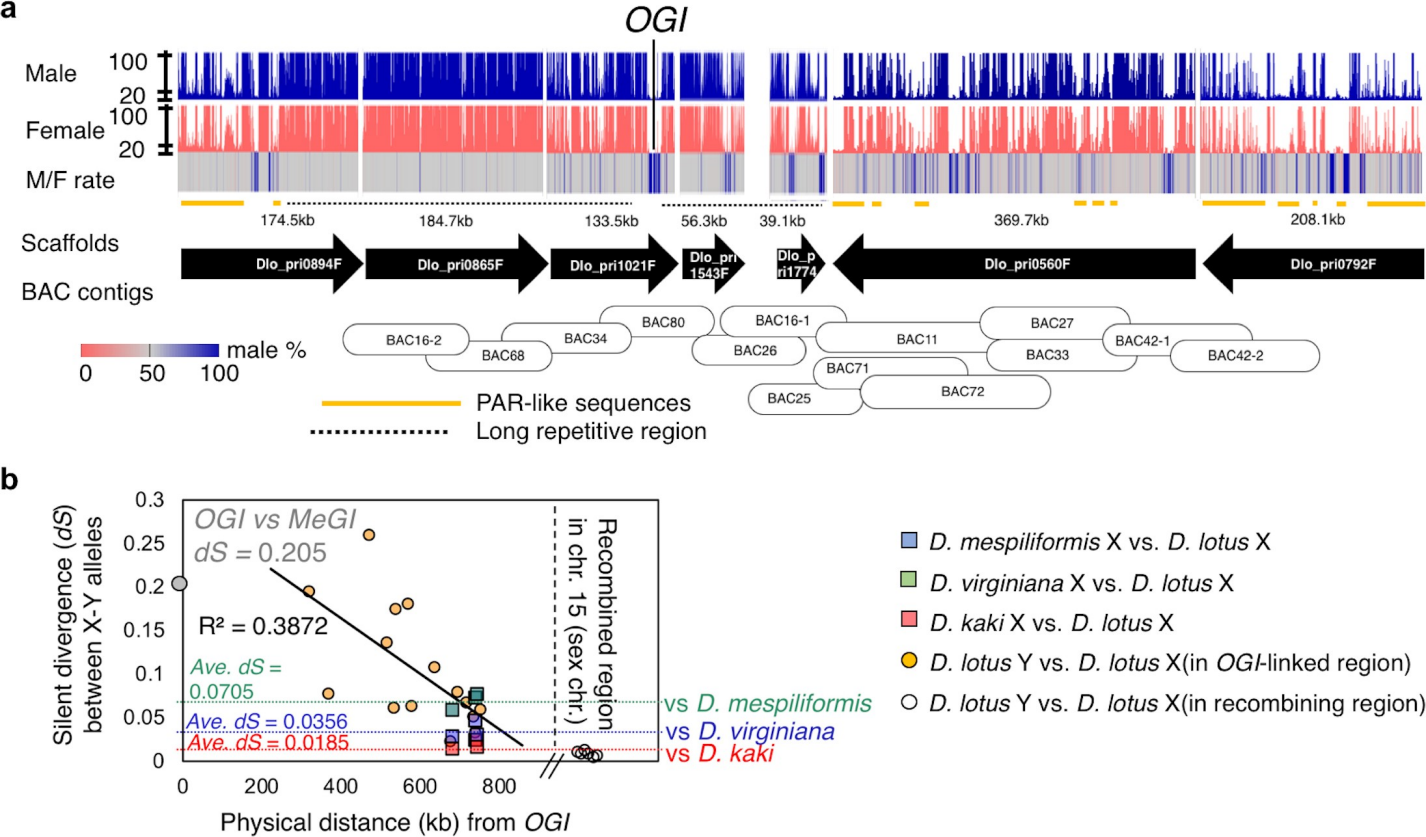
Genomic context of the Y-chromosomal region surrounding *OGI*. **a**, Read coverage from male (blue) and female (pink) samples and male/female coverage ratio across the scaffolds covering the male-specific region of the Y-chromosome. For both the male and female reads, expected coverage a single-copy sites is approximately 20 (grey lines across). This male-specific region was assembled via anchoring of the scaffolds with BAC sequences. Approximately 1.3Mb region was covered by Y-allelic scaffolds. More than 400kb of long repetitive sequences (dotted lines), flank *OGI*. Outer regions of these hyper repetitive sequences contain male-specific sequences (blue bands in M/F rate) and pseudo autosomal region (PAR)-like sequences (orange lines), where M/F rate was less than 70%, and the percentage of repetitive sequences was much lower. **b**, The silent divergence rate (*dS*) between X and Y alleles of the genes located in the PAR-like sequences (orange circles) decreases with distance to *OGI*. Stil, for most of these genes, the *dS* value between the X and Y alleles was larger than the average interspecific *dS* between the X alleles of *D*. *lotus* and *D*. *mespiliformis* (green square and dotted line), *D*. *lotus* and *D*. *virginiana* (blue square and dotted line), and *D*. *lotus* and *D*. *kaki* (red square dotted line). These results suggest that, in these PAR-like sequences, recombination between the X and Y alleles was suppressed before the divergence of *Diospyros* species, or at least predates the divergence between *D*. *lotus* and *D*. *kaki*. *dS* values for genes located in the regions closest to *OGI* are comparable to *dS* values between *OGI* and *MeGI* (gray circle, *dS* = 0.205), which suggest that little or no recombination occurred between these sequences after the establishment of *OGI*. In comparison, dS values between the X and Y alleles of genes located in the recombining region of chromosomes 15 are much lower (while circles on the right).
