# Supplementary material for: The persimmon genome reveals clues to the evolution of a lineage-specific sex determination system in plants
Source: PLoS Genet. 2020 Feb 18;16(2):e1008566. doi: 10.1371/journal.pgen.1008566 (PMC7048303; doi:10.1371/journal.pgen.1008566)
Supplement: S1 Fig — The distribution of distinct k-mers (k = 17) from the Illumina short reads showed two peaks at multiplicities of 32 and 54. The low and high peaks represent heterozygous and homozygous sequences, respectively. We estimated the genome size to be 877.7 Mb from the higher peak. This estimation almost agreed with the value measured by flow cytometry, 907 Mb, which was calculated from the nuclear DNA content in D. lotus of 1.85 pg/2 C (Tamura et al., 1998) and an assumption that 1 pg of DNA is equivalent to 980 Mb (Bennett et al., 2000). (PDF) [file pgen.1008566.s001.pdf]

Supplementary Information

S1 Figure: kmer distribution to estimate genome size and the degree of heterozygosity

The distribution of distinct *k*-mers (k = 17) from the Illumina short reads showed two peaks at multiplicities of 32 and 54. The low and high peaks represent heterozygous and homozygous sequences, respectively. We estimated the genome size to be 877.7 Mb from the higher peak. This estimation almost agreed with the value measured by flow cytometry, 907 Mb, which was calculated from the nuclear DNA content in *D. lotus* of 1.85 pg/2 C (Tamura et al., 1998) and an assumption that 1 pg of DNA is equivalent to 980 Mb (Bennett et al., 2000).

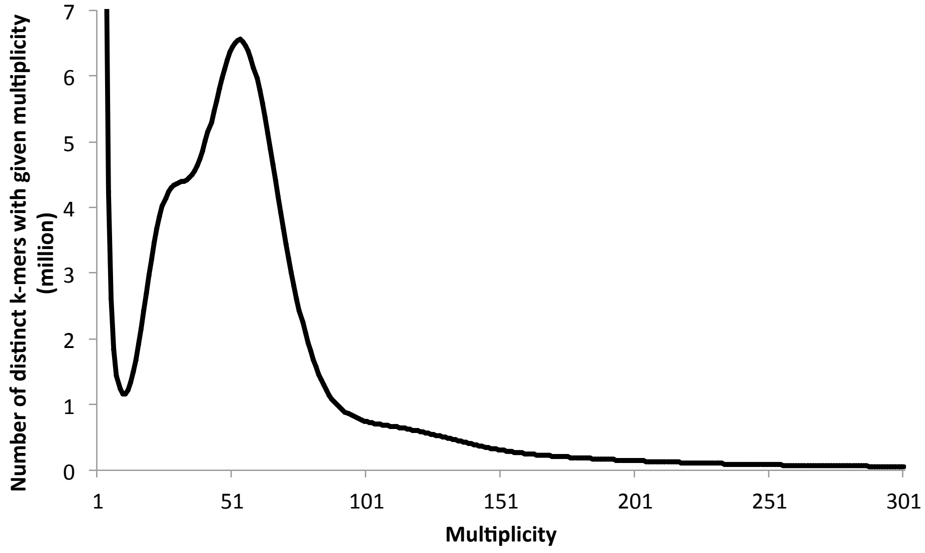

References

M. D. Bennett, P. Bhandol, I. J. Leitch, Nuclear DNA amounts in angiosperms and their modern uses - 807 new estimates. *Ann. Bot.* **86**, 859-909 (2000).

Tamura M, Tao R, Yonemori K, Ustunomiya N, Sugiura A. Ploidy level and genome size of several *Diospyros* species. J Jpn Soc Hortic Sci. 1998; 67: 306-312.
