# Supplementary material for: The persimmon genome reveals clues to the evolution of a lineage-specific sex determination system in plants
Source: PLoS Genet. 2020 Feb 18;16(2):e1008566. doi: 10.1371/journal.pgen.1008566 (PMC7048303; doi:10.1371/journal.pgen.1008566)

S2 Figure: Genetic anchoring data (Dlo01-15).

Genetic linkage map (left bars) and physical map of Dlo_r1.0 pseudomolecule sequences (right bars). Colors in the genetic map represent density of SNPs per 5 cM, while black, white and gray bars in the physical map indicate the positions of the forward, invert, and unknown directional scaffold sequences integrated in the pseudomolecule, respectively.

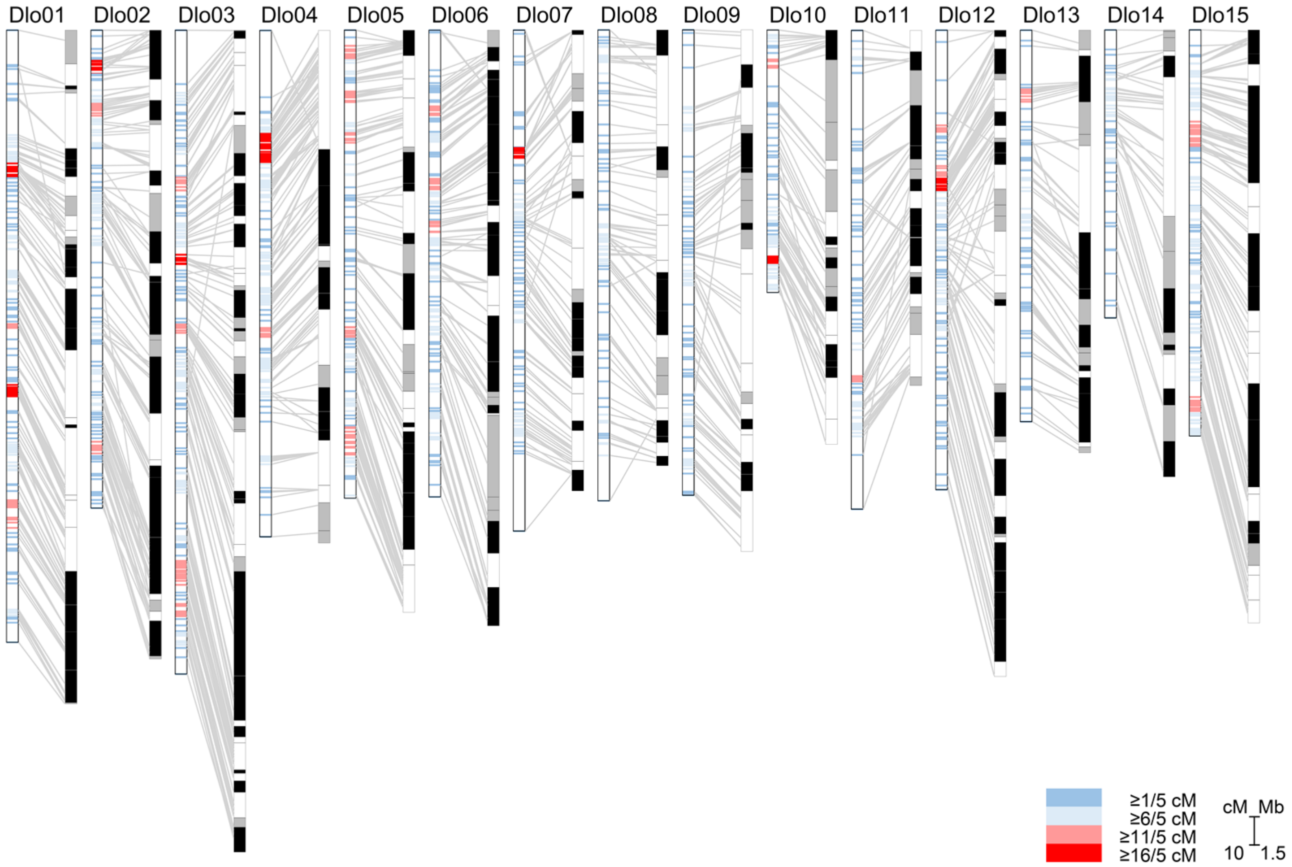

Supplement: S2 Fig — Genetic linkage map (left bars) and physical map of Dlo_r1.0 pseudomolecule sequences (right bars). Colors in the genetic map represent density of SNPs per 5 cM, while black, white and gray bars in the physical map indicate the positions of the forward, invert, and unknown directional scaffold sequences integrated in the pseudomolecule, respectively. (PDF) [file pgen.1008566.s002.pdf]
