# Supplementary material for: The persimmon genome reveals clues to the evolution of a lineage-specific sex determination system in plants
Source: PLoS Genet. 2020 Feb 18;16(2):e1008566. doi: 10.1371/journal.pgen.1008566 (PMC7048303; doi:10.1371/journal.pgen.1008566)

S3 Figure: Conservation of gene and repetitive sequences across representative plant species

a, Amino acid sequences were compared among genes from *D. lotus* (40,532 genes; DLO_r1.1 primary), *A. chinensis* (39,040 genes (Huang et al., 2013)), *V. vinifera* (29,927 genes (IGGP 12x.31) (Jaillon et al., 2009)), *S. lycopersicum* (34,789 genes (ITAG 3.10) (The Tomato Genome Consortium, 2012)), and *A. thaliana* (27,655 genes (Araport11) (Cheng et al., 2017)) using OrthoMCL v2.0.9 (Li et al., 2003) with default parameters. The numbers of clusters were shown in the intersections of the Venn diagram. **b,** Repetitive sequences were identified by RepeatMasker v4.0.6 (http://www.repeatmasker.org) using Repbase v406 (http://www.girinst.org/repbase/) and RepeatScout v1.0.5 for the genome sequences of *D. lotus* (8,974 sequences; DLO_r1.0), *A. chinensis* (30 pseudomolecules (Huang et al., 2013)), *S. lycopersicum* (13 pseudomolecules (SL3.0) (The Tomato Genome Consortium, 2012)), *Lactuca sativa* (lettuce; 9 pseudomolecules (V8) (Reyes-Chin-Wo et al., 2017)), *V. vinifera* (33 chromosomes (IGGP 12x.31) (Jaillon et al., 2009)), *Prunus persica* (peach; 8 pseudomolecules (v2.0.a1) (Verde et al., 2013), *Carica papaya* (papaya; 5,901 scaffold sequences (ASGPBv0.4) (Ming et al., 2008)), and *A. thaliana* (5 chromosomes, chloroplast and mitochondria genomes (TAIR10)). The percentage of repetitive sequences against the total length of the genome sequence were calculated for each of the result of RepeatMasker and RepeatScout and compared among the plant species.

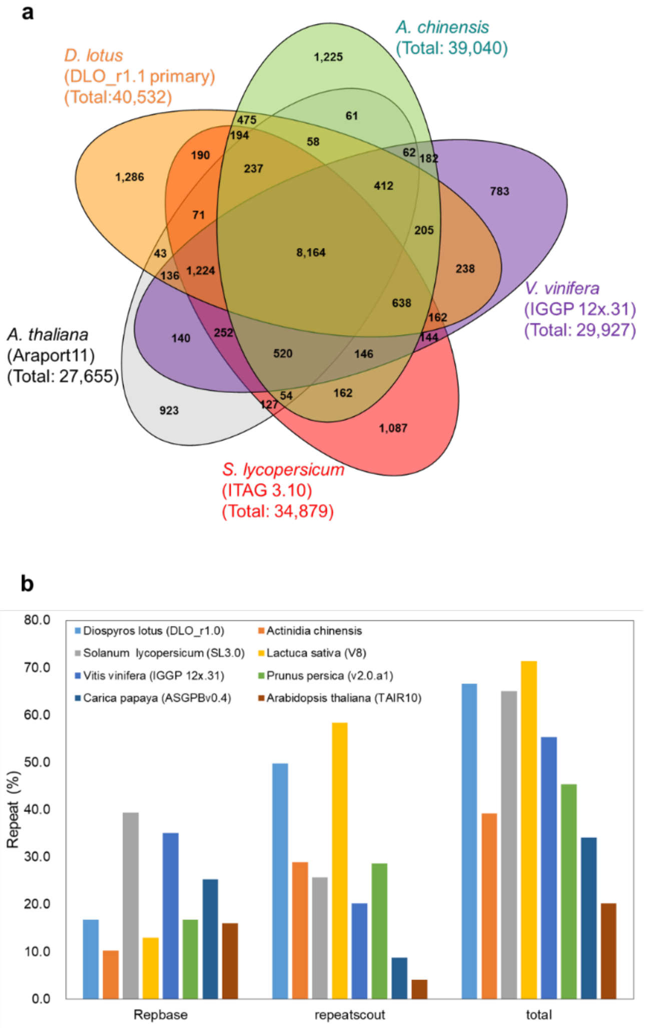

Supplement: S3 Fig — a, Amino acid sequences were compared among genes from D. lotus (40,532 genes; DLO_r1.1 primary), A. chinensis (39,040 genes (Huang et al., 2013)), V. vinifera (29,927 genes (IGGP 12x.31) (Jaillon et al., 2009)), S. lycopersicum (34,789 genes (ITAG 3.10) (The Tomato Genome Consortium, 2012)), and A. thaliana (27,655 genes (Araport11) (Cheng et al., 2017)) using OrthoMCL v2.0.9 (Li et al., 2003) with default parameters. The numbers of clusters were shown in the intersections of the Venn diagram. b, Repetitive sequences were identified by RepeatMasker v4.0.6 (http://www.repeatmasker.org) using Repbase v406 (http://www.girinst.org/repbase/) and RepeatScout v1.0.5 for the genome sequences of D. lotus (8,974 sequences; DLO_r1.0), A. chinensis (30 pseudomolecules (Huang et al., 2013)), S. lycopersicum (13 pseudomolecules (SL3.0) (The Tomato Genome Consortium, 2012)), Lactuca sativa (lettuce; 9 pseudomolecules (V8) (Reyes-Chin-Wo et al., 2017)), V. vinifera (33 chromosomes (IGGP 12x.31) (Jaillon et al., 2009)), Prunus persica (peach; 8 pseudomolecules (v2.0.a1) (Verde et al., 2013), Carica papaya (papaya; 5,901 scaffold sequences (ASGPBv0.4) (Ming et al., 2008)), and A. thaliana (5 chromosomes, chloroplast and mitochondria genomes (TAIR10)). The percentage of repetitive sequences against the total length of the genome sequence were calculated for each of the result of RepeatMasker and RepeatScout and compared among the plant species. (PDF) [file pgen.1008566.s003.pdf]
