# Supplementary material for: The persimmon genome reveals clues to the evolution of a lineage-specific sex determination system in plants
Source: PLoS Genet. 2020 Feb 18;16(2):e1008566. doi: 10.1371/journal.pgen.1008566 (PMC7048303; doi:10.1371/journal.pgen.1008566)

S4 Figure: Genome-wide syntenic analysis

a, Synteny analysis based on gene order using CoGe SynMap, and using only the gene pairs identified as putatively derived from the *Dd-α,* with *dS* values between 0.5 and 0.9. The masked syntenic blocks (*dS* < 0.5 or *dS* > 0.9) are shown in gray. Syntenic blocks were detected with (B)lastz using default parameters. Long syntenic blocks with *dS* = 0.5-0.9 were conserved throughout the genome, and were consistent with the main duplicated blocks shown in Figure 1. Panel b is more detailed view of the region highlighted by red rectangles and annotated Syn I. **b**, Example of a large syntenic CDS blocks of genes with *dS* values between 0.5 and 0.9, between chromosome 1 and 2.

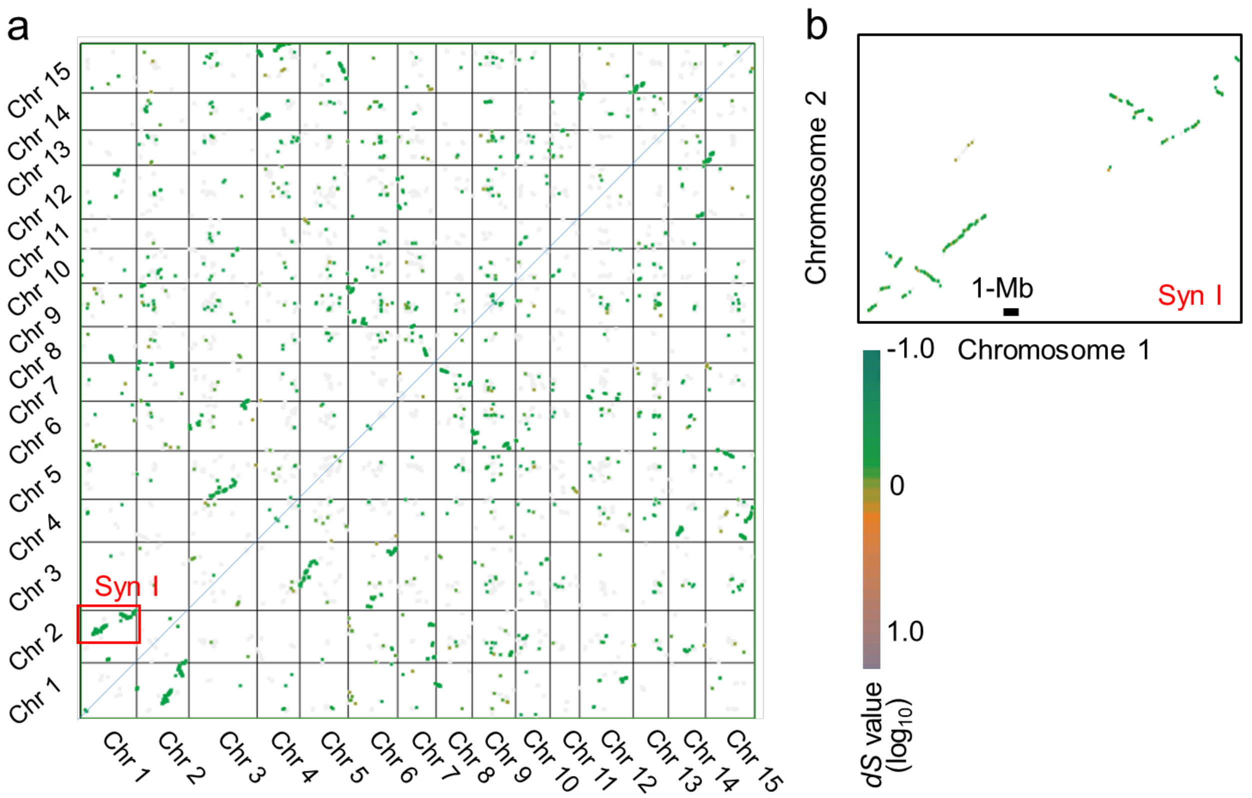

Supplement: S4 Fig — a, Synteny analysis based on gene order using CoGe SynMap, and using only the gene pairs identified as putatively derived from the Dd-α, with dS values between 0.5 and 0.9. The masked syntenic blocks (dS < 0.5 or dS > 0.9) are shown in gray. Syntenic blocks were detected with (B)lastz using default parameters. Long syntenic blocks with dS = 0.5–0.9 were conserved throughout the genome, and were consistent with the main duplicated blocks shown in Fig 1. Panel b is more detailed view of the region highlighted by red rectangles and annotated Syn I. b, Example of a large syntenic CDS blocks of genes with dS values between 0.5 and 0.9, between chromosome 1 and 2. (PDF) [file pgen.1008566.s004.pdf]
