# Supplementary material for: The persimmon genome reveals clues to the evolution of a lineage-specific sex determination system in plants
Source: PLoS Genet. 2020 Feb 18;16(2):e1008566. doi: 10.1371/journal.pgen.1008566 (PMC7048303; doi:10.1371/journal.pgen.1008566)

S5 Figure: Gene duplication patterns following the *Dd-α* event.

a, Heat map for the numbers of genes derived from *Dd-α* (*dS* values between 0.5 and 0.9), shared between two chromosomes. For instance, Dlo01 (Chromosome 1) shared many paralogs with Dlo02 (Chromosome 2) and Dlo12 (Chromosome 12), while Dlo02 and Dlo12 shared few paralogs with each other. The patterns of such affinities between the chromosomes suggests a paleotetraploidization event. **b,** Syntenic relationship between the putatively *Dd-α*-derived paralogous genes within the *Diospyros* genome.

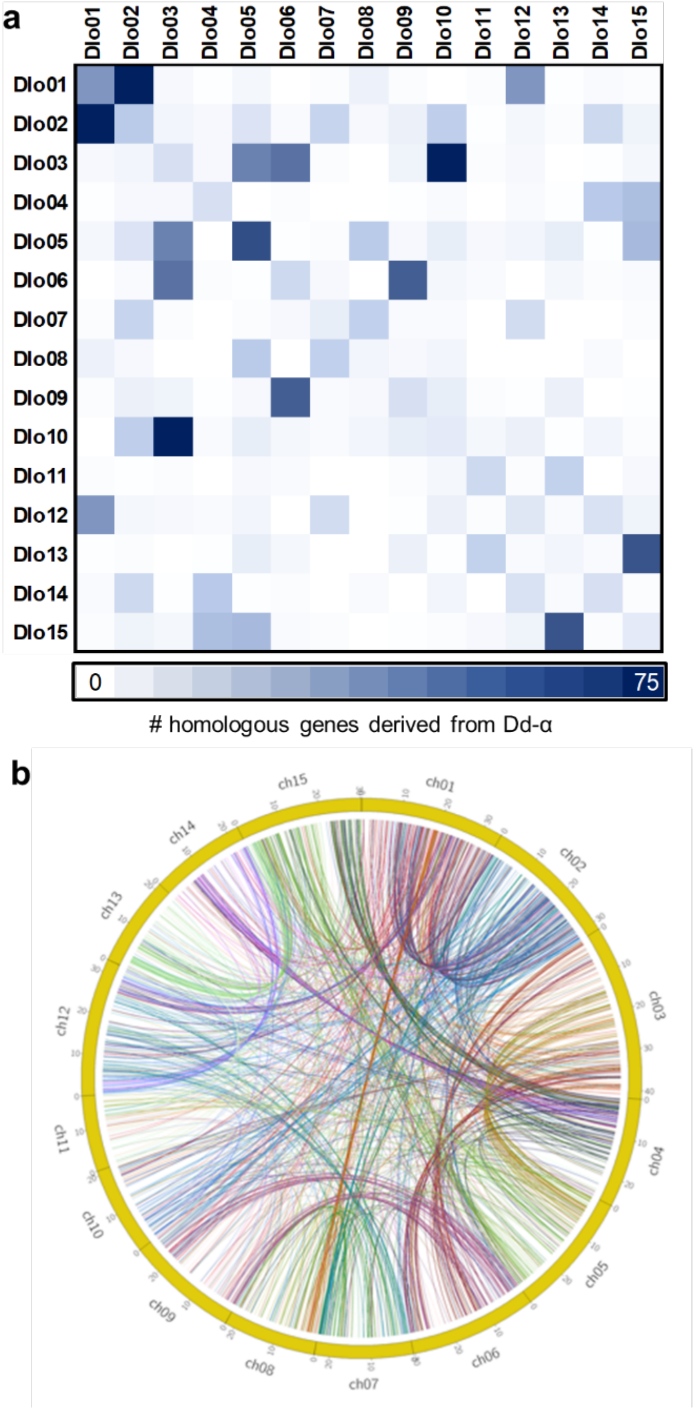

Supplement: S5 Fig — a, Heat map for the numbers of genes derived from Dd-α, shared between two chromosomes. For instance, Dlo01 shared many paralogs with Dlo02 and Dlo12, while Dlo02 and Dlo12 shared few paralogs with each other. The patterns of such affinities between the chromosomes suggests a paleotetraploidization event. b, Syntenic relationship between the putatively Dd-α-derived paralogous genes within the Diospyros genome. (PDF) [file pgen.1008566.s005.pdf]
