# Supplementary material for: The persimmon genome reveals clues to the evolution of a lineage-specific sex determination system in plants
Source: PLoS Genet. 2020 Feb 18;16(2):e1008566. doi: 10.1371/journal.pgen.1008566 (PMC7048303; doi:10.1371/journal.pgen.1008566)

S6 Figure: Genome-wide synteny between *Diospyros* and *Actinidia*

Dot plots of the syntenic genomic regions between *Diospyros* and *Actinidia*. As represented in the orange box, a single genomic segment from *Actinidia* corresponds to two syntenic *Diospyros* genome regions which are derived from the *Dd-α*. In the orange box, the middle regions of Dlo01 and Dlo02, and Dlo03 and Dlo06 are duplicated regions via *Dd-α* (see Figure 1g and S4-5 Figure). On the other hand, as represented in the green box, a genomic segment from *Diospyros* corresponds to at maximum four syntenic *Actinidia* genome regions which are derived from the double *Actinidia*-specific genome-wide duplication events (Ad-α and Ad-β) (Huang et al., 2013). These results indicate that, in the evolution of the order Ericales, *Dd-α* and Ad-α/β occurred independently in the *Diospyros* and *Actinidia* ancestral genomes, respectively.

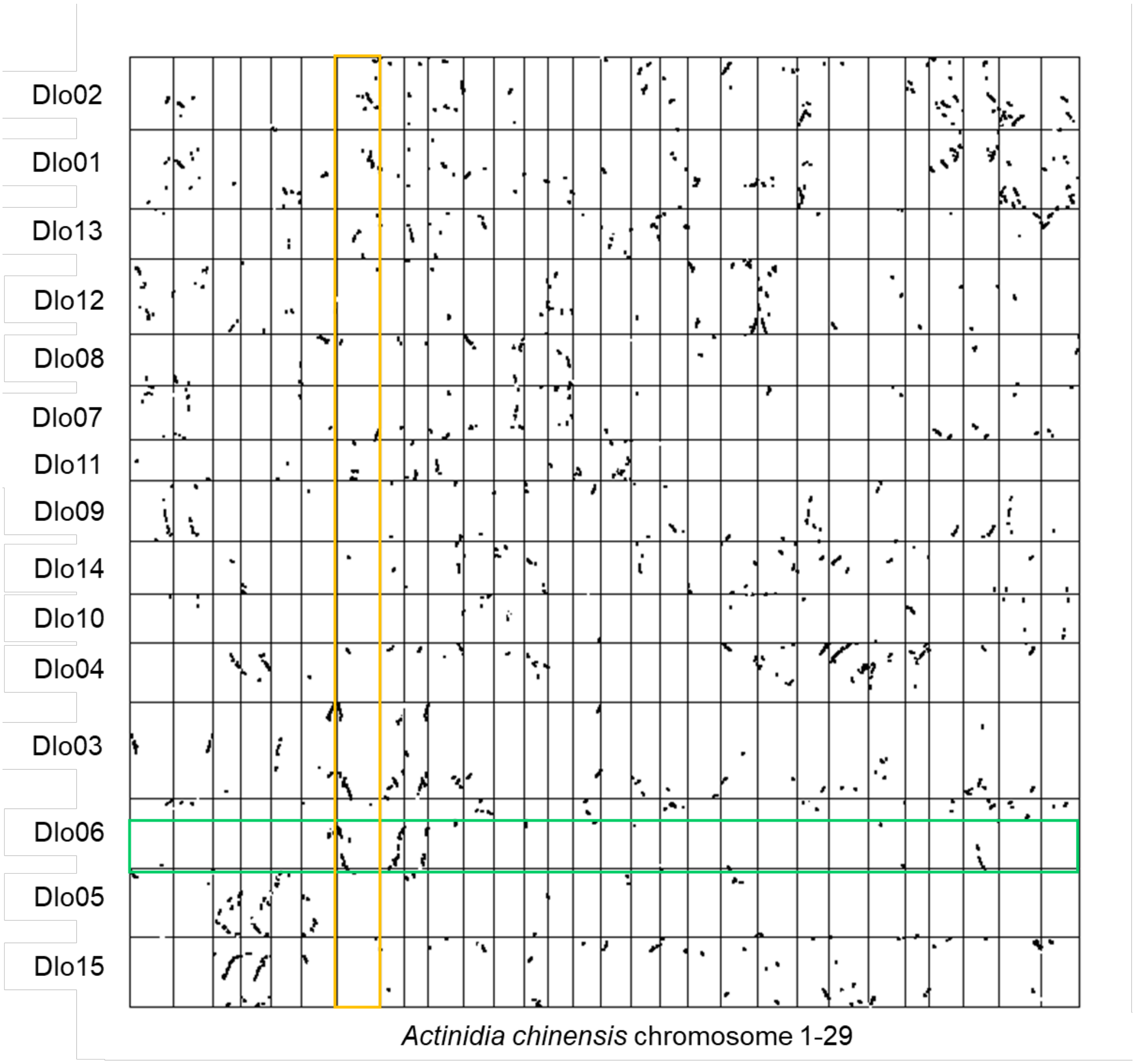

Supplement: S6 Fig — Dot plots of the syntenic genomic regions between Diospyros and Actinidia. As represented in the orange box, a single genomic segment from Actinidia corresponds to two syntenic Diospyros genome regions which are derived from the Dd-α. In the orange box, the middle regions of Dlo01 and Dlo02, and Dlo03 and Dlo06 are duplicated regions via Dd-α (see Fig 1 and S4 Fig). On the other hand, as represented in the green box, a genomic segment from Diospyros corresponds to at maximum four syntenic Actinidia genome regions which are derived from the double Actinidia-specific whole-genome duplication events (Ad-α and Ad-β) (Huang et al., 2013). These results indicate that, in the evolution of the order Ericales, Dd-α and Ad-α/β occurred independently in the Diospyros and Actinidia ancestral genomes, respectively. (PDF) [file pgen.1008566.s006.pdf]
