# Supplementary material for: The persimmon genome reveals clues to the evolution of a lineage-specific sex determination system in plants
Source: PLoS Genet. 2020 Feb 18;16(2):e1008566. doi: 10.1371/journal.pgen.1008566 (PMC7048303; doi:10.1371/journal.pgen.1008566)

**S7 Figure: Physical relationships of the syntenic segments between the regions surrounding *MeGI* and *SiMeGI***

Syntenic relationships in the *MeGI* and *SiMeGI* surrounding regions, using GEvo (CoGe). The high-scoring segment pairs (HSP) detected are shown here connected with red lines. They correspond to gene pairs with *dS* values ranging between 0.5 and 0.9, or regions flanking genes, in the Dlo_pri0025F and Dlo_pri0799F genomic contigs.

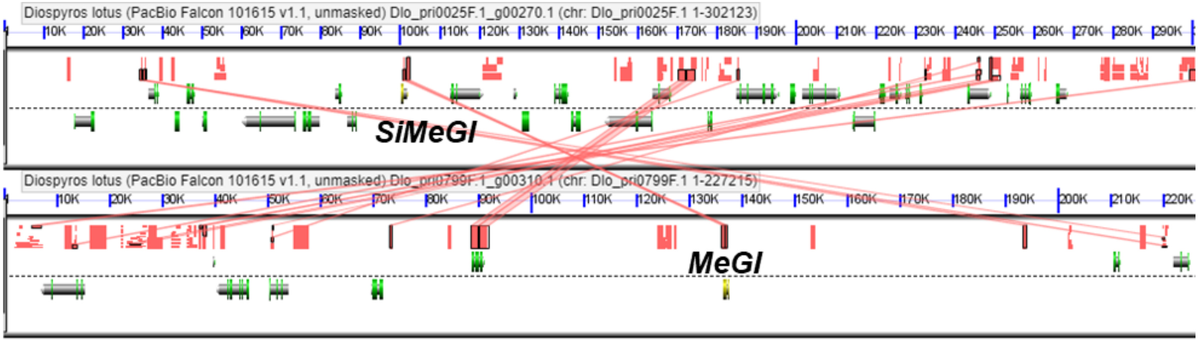

Supplement: S7 Fig — Syntenic relationships in the MeGI and SiMeGI surrounding regions, using GEvo (CoGe). The high-scoring segment pairs (HSP) detected are shown here connected with red lines. They correspond to gene pairs with dS values ranging between 0.5 and 0.9, or regions flanking genes, in the Dlo_pri0025F and Dlo_pri0799F genomic contigs. (PDF) [file pgen.1008566.s007.pdf]
