# Supplementary material for: The persimmon genome reveals clues to the evolution of a lineage-specific sex determination system in plants
Source: PLoS Genet. 2020 Feb 18;16(2):e1008566. doi: 10.1371/journal.pgen.1008566 (PMC7048303; doi:10.1371/journal.pgen.1008566)

S8 Figure: Phylogenetic tree of the angiosperm HD-ZIP1 family.

Phylogeny of the HD-Zip1 type homeodomain genes in representative angiosperm genomes (*Solanum lycopersicum, Oryza sativa, Zea mays*, and *Arabidopsis thaliana*) and the *D. lotus* genome. The 175 HD-ZIP1 genes clustered into 8 major clades (Clade I-VIII), which each included at least one homolog from all 5 species used in this study (see Materials and Methods). It is important to note that the root branches of clades VI and VII were not statistically significant though (32/100 and 28/100, respectively). *MeGI* and *SiMeGI* from persimmon, the three closest orthologs from Arabidopsis, *Vrs1* from barley and *LOC_Os07g39320* from rice were all nested within clade IV (colored in light green). No other persimmon paralog nested into clade IV (bootstrap = 100/100). This suggested that the other persimmon paralogs had diverged from *MeGI* and *SiMeGI* before the divergence of the angiosperms (or the time of divergence between monocots and dicots).

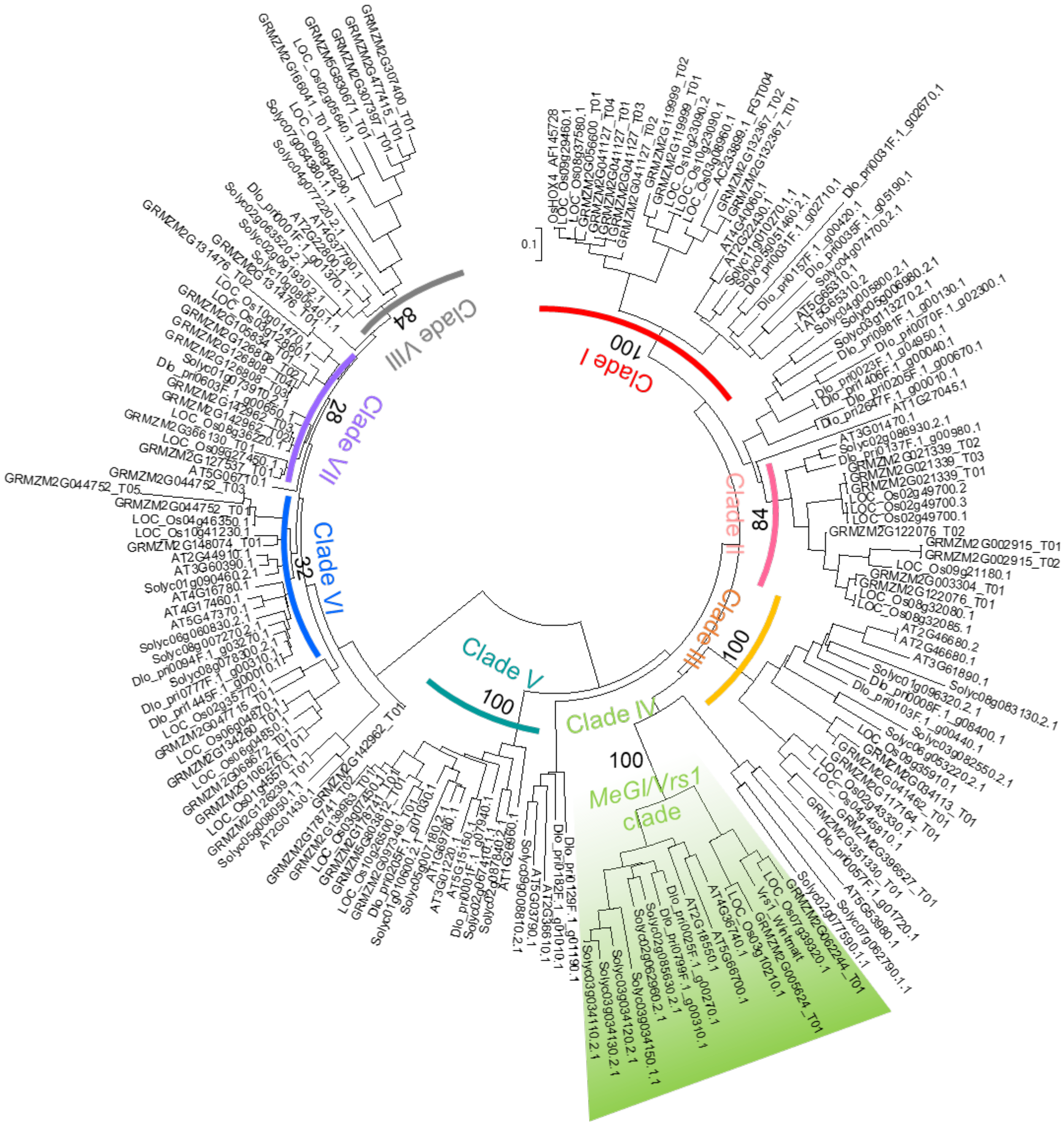

Supplement: S8 Fig — Phylogeny of the HD-Zip1 type homeodomain genes in representative angiosperm genomes (Solanum lycopersicum, Oryza sativa, Zea mays, and Arabidopsis thaliana) and the D. lotus genome. The 175 HD-ZIP1 genes clustered into 8 major clades (Clade I-VIII), which each included at least one homolog from all 5 species used in this study (see Materials and Methods). It is important to note that the root branches of clades VI and VII were not statistically significant though (32/100 and 28/100, respectively). MeGI and SiMeGI from persimmon, the three closest orthologs from Arabidopsis, Vrs1 from barley and LOC_Os07g39320 from rice were all nested within clade IV (colored in light green). No other persimmon paralog nested into clade IV (bootstrap = 100/100). This suggested that the other persimmon paralogs had diverged from MeGI and SiMeGI before the divergence of the angiosperms (or the time of divergence between monocots and dicots). (PDF) [file pgen.1008566.s008.pdf]
