# Supplementary material for: The persimmon genome reveals clues to the evolution of a lineage-specific sex determination system in plants
Source: PLoS Genet. 2020 Feb 18;16(2):e1008566. doi: 10.1371/journal.pgen.1008566 (PMC7048303; doi:10.1371/journal.pgen.1008566)

S9 Figure: Overexpression of *MeGI* and *SiMeGI* under the control of CaMV35S promoter in *N. tabacum*

a-c, 1-week old transgenic lines. The *MeGI*-induced lines (**a**) frequently showed clear irregularities in development, in comparison to the *SiMeGI* lines (**b**) or empty cassette-induced lines (**c**). **d**, Comparison of 4-weeks old transgenic plants. The *MeGI*-induced lines (center) uniformly showed more severe growth inhibition, than the *SiMeGI*-induced lines (left). **e**, close-up picture of the *MeGI*-induced line corresponding to the individual marked with an asterisk in the panel (**d**). The leaves showed irregular shapes with significantly less veins. **f**, comparison of the appearance of 15-weeks old transgenic lines. The *MeGI*-induced line (left) exhibited dwarfism, but the total number of leaves were comparable to the control plants (right), while the internode lengths were shorter than the control, as shown in the panel "**g**". **h**, Differentiation of the leave shapes and structures in the control (left) and the *MeGI*-induced line (right). The *MeGI*-induced lines produced narrow and serrated leaves. Bars indicate 10mm for a-c, and e; 50mm for d, f, g, and h.

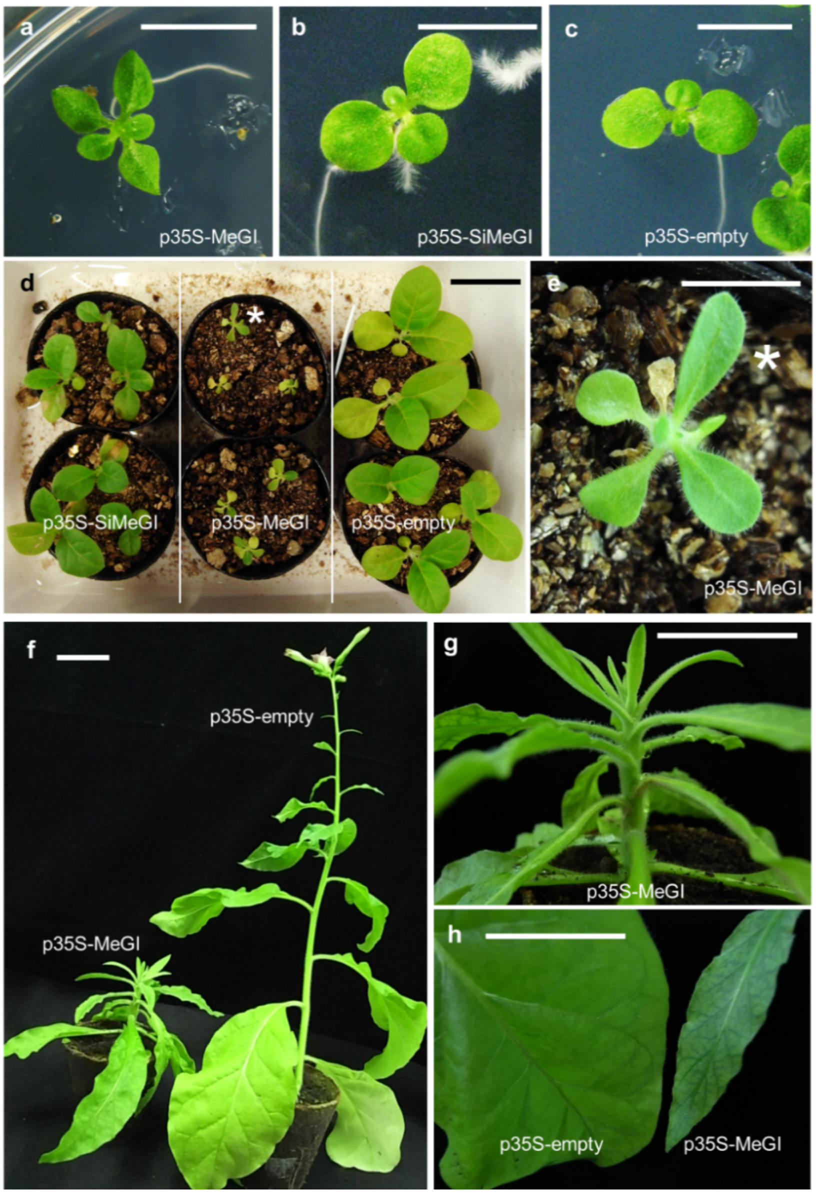

Supplement: S9 Fig — a-c, 1-week old transgenic lines. The MeGI-induced lines (a) frequently showed clear irregularities in development, in comparison to the SiMeGI lines (b) or empty cassette-induced lines (c). d, Comparison of 4-weeks old transgenic plants. The MeGI-induced lines (center) uniformly showed more severe growth inhibition, than the SiMeGI-induced lines (left). e, close-up picture of the MeGI-induced line corresponding to the individual marked with an asterisk in the panel (d). The leaves showed irregular shapes with significantly less veins. f, comparison of the appearance of 15-weeks old transgenic lines. The MeGI-induced line (left) exhibited dwarfism, but the total number of leaves were comparable to the control plants (right), while the internode lengths were shorter than the control, as shown in the panel “g”. h, Differentiation of the leave shapes and structures in the control (left) and the MeGI-induced line (right). The MeGI-induced lines produced narrow and serrated leaves. Bars indicate 10mm for a-c, and e; 50mm for d, f, g, and h. (PDF) [file pgen.1008566.s009.pdf]
