# Supplementary material for: The persimmon genome reveals clues to the evolution of a lineage-specific sex determination system in plants
Source: PLoS Genet. 2020 Feb 18;16(2):e1008566. doi: 10.1371/journal.pgen.1008566 (PMC7048303; doi:10.1371/journal.pgen.1008566)

**S10 Figure: Overexpression of *MeGI* and *SiMeGI* under the control of CaMV35S promoter in *A. thaliana*.**

**a**, Dissection of the control Arabidopsis plant transformed with an empty cassette. an: anther, pe: petal, sg: stigma. **b-e**, p35S-*MeGI* transgenic lines. Dissected flowers show rudimental anthers (ra) (**b-c**). Approximately half of the transgenic plants are semi-dwarf (semi-dwf) (**d**) or complete dwarf (**e**). They also frequently showed leaf serration, which is consistent with our previous analysis of the p35S-*MeGI* induced Arabidopsis plants (Akagi et al., 2014). **f-i**, p35S-*SiMeGI* transgenic lines. The transgenic plants occasionally showed rudimental anthers similar to the *MeGI*-induced lines (**f-g**). A part of the *SiMeGI*-induced lines showed semi-dwarfism (**h**), but full dwarfism was never observed in the 63 transgenic lines. Over 95% of the *SiMeGI*-induced lines were hermaphroditic, where the numbers of stamens are properly maintained (**i**), in contrast to the *MeGI*-induced lines (Akagi et al., 2014). Bars indicate 1mm for a, b, f, and i; 0.1mm for c and g; 10mm for d-e and h. j, Distribution of the number of female (fe) and hermaphrodite (herm) individuals in the p35-*MeGI* (green), p35S-*SiMeGI* (yellow), and p35S-empty (cont; gray) transgenic lines. k, Distribution of the number of complete dwarf (dwf), semi-dwarf (semi-dwf) and normal individuals in the p35-*MeGI*, p35S-*SiMeGI*, and p35S-empty (cont) transgenic lines.

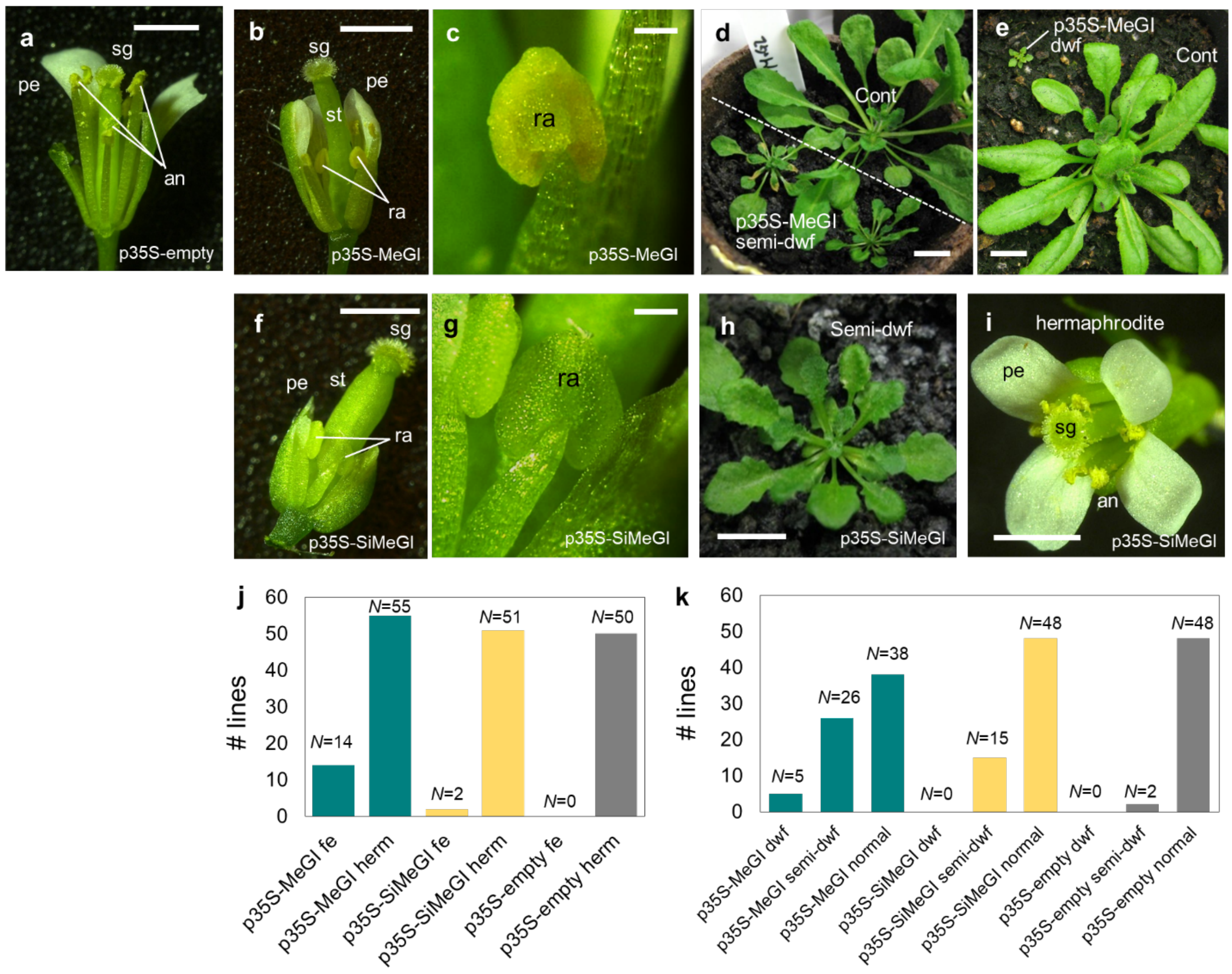

Supplement: S10 Fig — a, Dissection of the control Arabidopsis plant transformed with an empty cassette. an: anther, pe: petal, sg: stigma. b-e, p35S-MeGI transgenic lines. Dissected flowers show rudimental anthers (ra) (b-c). Approximately half of the transgenic plants are semi-dwarf (semi-dwf) (d) or complete dwarf (e). They also frequently showed leaf serration, which is consistent with our previous analysis of the p35S-MeGI induced Arabidopsis plants (Akagi et al., 2014). f-i, p35S-SiMeGI transgenic lines. The transgenic plants occasionally showed rudimental anthers similar to the MeGI-induced lines (f-g). A part of the SiMeGI-induced lines showed semi-dwarfism (h), but full dwarfism was never observed in the 63 transgenic lines. Over 95% of the SiMeGI-induced lines were hermaphroditic, where the numbers of stamens are properly maintained (i), in contrast to the MeGI-induced lines (Akagi et al., 2014). Bars indicate 1mm for a, b, f, and i; 0.1mm for c and g; 10mm for d-e and h. j, Distribution of the number of female (fe) and hermaphrodite (herm) individuals in the p35-MeGI (green), p35S-SiMeGI (yellow), and p35S-empty (cont; gray) transgenic lines. k, Distribution of the number of complete dwarf (dwf), semi-dwarf (semi-dwf) and normal individuals in the p35-MeGI, p35S-SiMeGI, and p35S-empty (cont) transgenic lines. (PDF) [file pgen.1008566.s010.pdf]
