# Supplementary material for: The persimmon genome reveals clues to the evolution of a lineage-specific sex determination system in plants
Source: PLoS Genet. 2020 Feb 18;16(2):e1008566. doi: 10.1371/journal.pgen.1008566 (PMC7048303; doi:10.1371/journal.pgen.1008566)

S11 Figure: *in situ* RNA hybridization

RNA *in situ* hybridization in developing buds and flower primordia, using *MeGI* (**a-c**) and *SiMeGI* (**d-f**) sequences as probes. In the cross section of the developing buds (**a** and **d**), the *MeGI* signal is strong in flower buds only (fb) (**a**), while *SiMeGI* showed significant signal in the pith (Pi) and young leaves (ly), as well as in flower buds (**d**). This is consistent with our expression analyses using laser capture micro-dissected (LCM) samples (S12 Figure). In the longitudinal sections of the developing buds (**b** and **e**), both *MeGI* and *SiMeGI* signals are confined to the meristematic region, especially in the shoot apical meristems (sam). At a later developing stage (**c** and **f**), flower primordia (fp) and bract (br) showed substantial signals of both *MeGI* (**c**) and *SiMeGI* (**f**). Bars indicate 50μm.

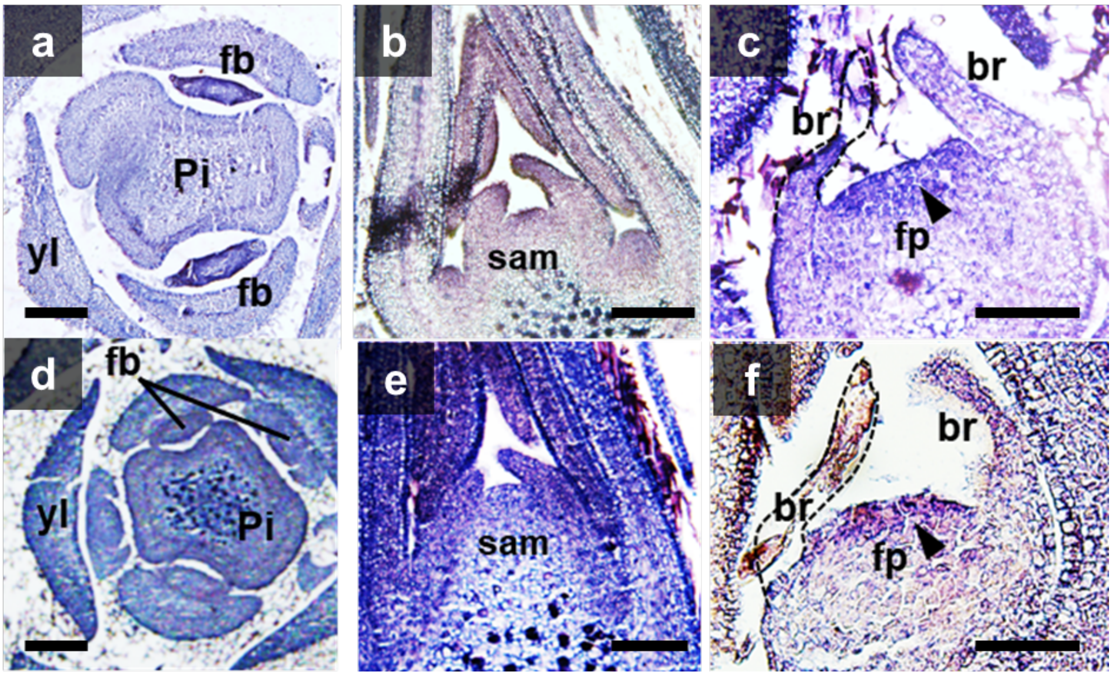

Supplement: S11 Fig — RNA in situ hybridization in developing buds and flower primordia, using MeGI (a-c) and SiMeGI (d-f) sequences as probes. In the cross section of the developing buds (a and d), the MeGI signal is strong in flower buds only (fb) (a), while SiMeGI showed significant signal in the pith (Pi) and young leaves (ly), as well as in flower buds (d). This is consistent with our expression analyses using laser capture micro-dissected (LCM) samples (S12 Fig). In the longitudinal sections of the developing buds (b and e), both MeGI and SiMeGI signals are confined to the meristematic region, especially in the shoot apical meristems (sam). At a later developing stage (c and f), flower primordia (fp) and bract (br) showed substantial signals of both MeGI (c) and SiMeGI (f). Bars indicate 50μm. (PDF) [file pgen.1008566.s011.pdf]
