# Supplementary material for: The persimmon genome reveals clues to the evolution of a lineage-specific sex determination system in plants
Source: PLoS Genet. 2020 Feb 18;16(2):e1008566. doi: 10.1371/journal.pgen.1008566 (PMC7048303; doi:10.1371/journal.pgen.1008566)

S12 Figure: Expression analysis of *MeGI/SiMeGI* in laser capture microdissection (LCM)

Longitudinal (**a**) and cross (**b**) sections of buds from *D. lotus*, Kunsenshi-male, and the target of the LCM. We targeted flower buds (red), young leaf or leaf buds (blue), and pith or cambium (green). **c**, the section after laser captions. **d**, qRT-PCR analysis to detect relative expression of the *MeGI* and *SiMeGI* among the organs, at early developmental stages (Jun-Jul) when the flower primordia form. Consistent with the results of the in situ hybridization (S11 Figure), *MeGI* expression was much stronger in flower buds than in pith or young leaves, while the difference in expression levels between the three organs was less drastic for *SiMeGI*. For both graphs, the expression level in flower buds was defined as “1”. **e**, comparison of the expression level of *MeGI* and *SiMeGI* in the developing flower buds. Illumina mRNA-Seq analysis was conducted on the LCM samples to detect RPKM values of *MeGI* and *SiMeGI*. *SiMeGI* was expressed higher than or comparative to the *MeGI,* in the developing flower buds. Notwithstanding, the reduction in *MeGI* expression in this stage affect the flower sexuality and the inflorescent structure (Akagi et al., 2014). **f**, relative expression of the *MeGI* and *SiMeGI* in different organs, during dormancy stage (Dec) when the development of flower primordia halt. Flower buds showed no significant expression of either *MeGI* or *SiMeGI*.

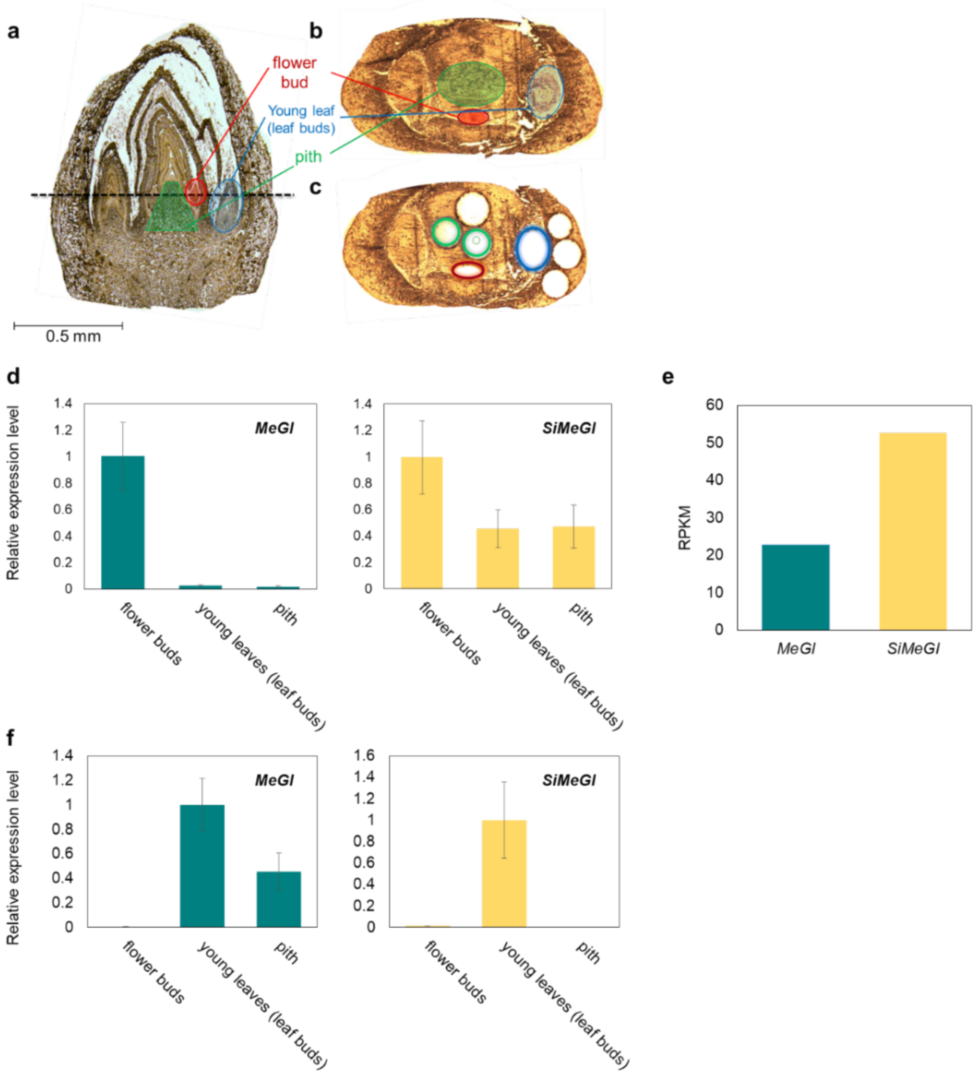

Supplement: S12 Fig — Longitudinal (a) and cross (b) sections of buds from D. lotus, Kunsenshi-male, and the target of the LCM. We targeted flower buds (red), young leaf or leaf buds (blue), and pith or cambium (green). c, the section after laser captions. d, qRT-PCR analysis to detect relative expression of the MeGI and SiMeGI among the organs, at early developmental stages (Jun-Jul) when the flower primordia form. Consistent with the results of the in situ hybridization (S11 Fig), MeGI expression was much stronger in flower buds than in pith or young leaves, while the difference in expression levels between the three organs was less drastic for SiMeGI. For both graphs, the expression level in flower buds was defined as “1”. e, comparison of the expression level of MeGI and SiMeGI in the developing flower buds. Illumina mRNA-Seq analysis was conducted on the LCM samples to detect RPKM values of MeGI and SiMeGI. SiMeGI was expressed higher than or comparative to the MeGI, in the developing flower buds. Notwithstanding, the reduction in MeGI expression in this stage affect the flower sexuality and the inflorescent structure (Akagi et al., 2014). f, relative expression of the MeGI and SiMeGI in different organs, during dormancy stage (Dec) when the development of flower primordia halt. Flower buds showed no significant expression of either MeGI or SiMeGI. (PDF) [file pgen.1008566.s012.pdf]
