# Supplementary material for: The persimmon genome reveals clues to the evolution of a lineage-specific sex determination system in plants
Source: PLoS Genet. 2020 Feb 18;16(2):e1008566. doi: 10.1371/journal.pgen.1008566 (PMC7048303; doi:10.1371/journal.pgen.1008566)

**S13 Figure: Structure of the sequence surrounding *OGI*.**

Self-syntenic collinearity was detected in the Y-chromosomal region flanking *OGI* (Scaffold Dlo_pri1021F.1). Inverted and forwarded repeat blocks were frequently conserved, of which some act for small-RNA productions. Forward and reverse syntenic strands are shown in red and blue, respectively.

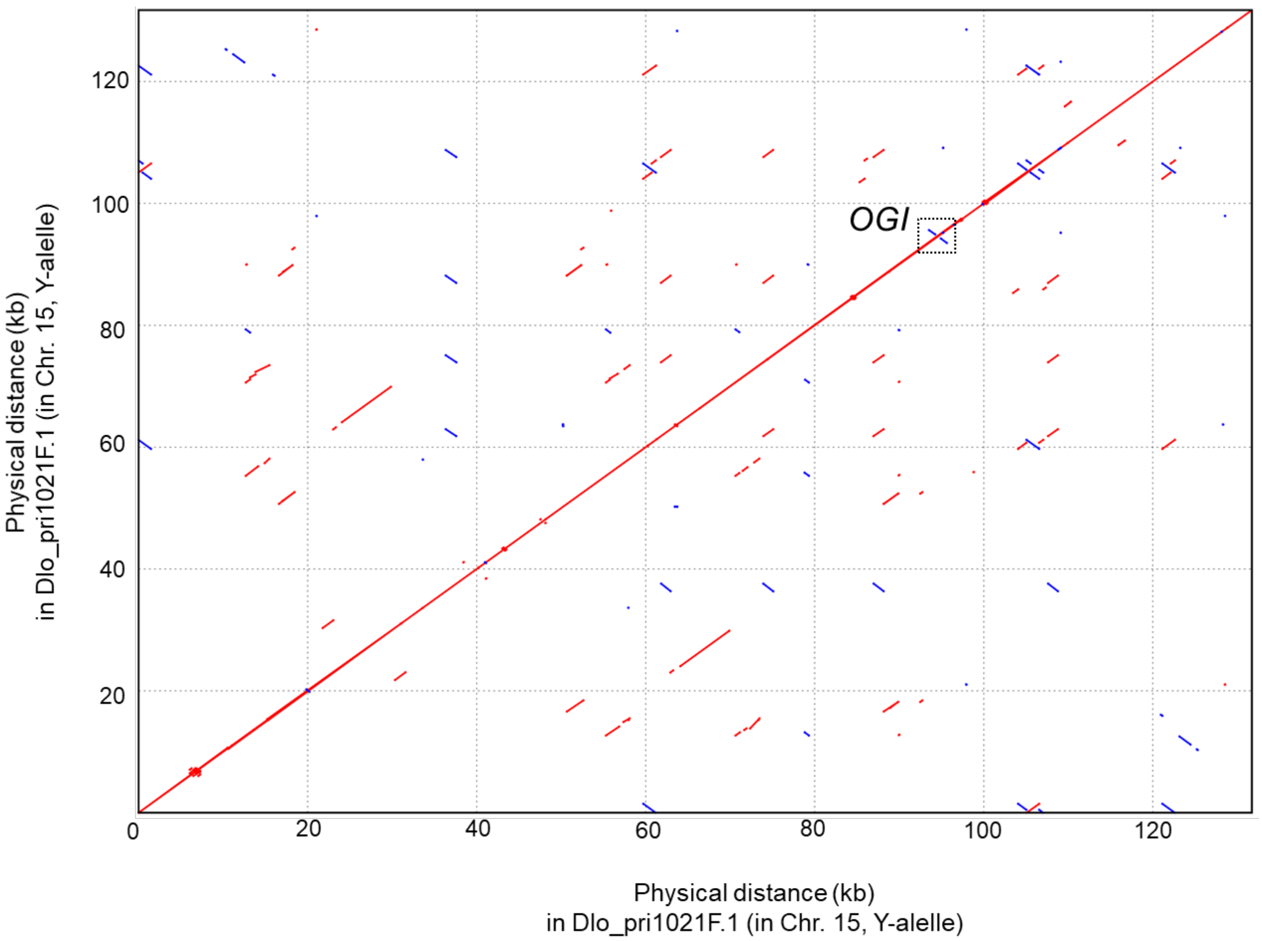

Supplement: S13 Fig — Self-syntenic collinearity was detected in the Y-chromosomal region flanking OGI (Scaffold Dlo_pri1021F.1). Inverted and forwarded repeat blocks were frequently conserved, of which some act for small-RNA productions. Forward and reverse syntenic strands are shown in red and blue, respectively. (PDF) [file pgen.1008566.s013.pdf]
