# Supplementary material for: The persimmon genome reveals clues to the evolution of a lineage-specific sex determination system in plants
Source: PLoS Genet. 2020 Feb 18;16(2):e1008566. doi: 10.1371/journal.pgen.1008566 (PMC7048303; doi:10.1371/journal.pgen.1008566)

S14 Figure: Syntenic analysis between the regions surrounding *OGI* and *MeGI*.

Scaffold Dlo_pri0799F.1, which includes *MeGI* on Chr. 13, and scaffold Dlo_pri1021F.1, which includes *OGI* on Chr. 15, were aligned to each other to detect syntenic blocks. Both scaffolds are continuous and devoid of sequence gaps. Segmental collinearity was not detected between these regions, except for the transcriptional regions of *OGI* and *MeGI*.

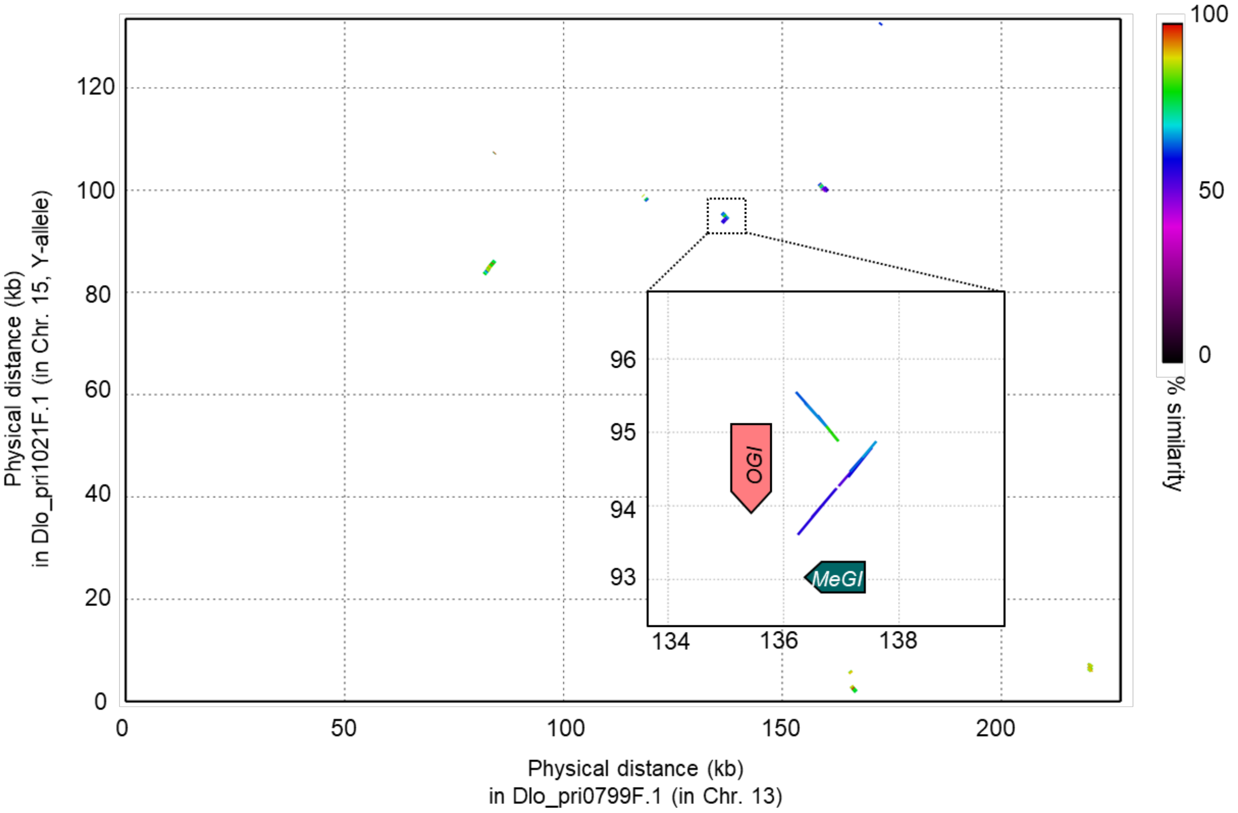

Supplement: S14 Fig — Scaffold Dlo_pri0799F.1, which includes MeGI on Chr. 13, and scaffold Dlo_pri1021F.1, which includes OGI on Chr. 15, were aligned to each other to detect syntenic blocks. Both scaffolds are continuous and devoid of sequence gaps. Segmental collinearity was not detected between these regions, except for the transcriptional regions of OGI and MeGI. (PDF) [file pgen.1008566.s014.pdf]
