# Supplementary material for: The persimmon genome reveals clues to the evolution of a lineage-specific sex determination system in plants
Source: PLoS Genet. 2020 Feb 18;16(2):e1008566. doi: 10.1371/journal.pgen.1008566 (PMC7048303; doi:10.1371/journal.pgen.1008566)

**S15 Figure: Phylogenetic analysis of the establishment of *MeGI*, proto *OGI*, and the inverted repeat of *OGI*.**

The nucleotide sequences of *SiMeGI*, *MeGI*, and the forward and inverted repeats of *OGI* were aligned to each other to estimate their relative timing of establishment. Our results indicated that the gene duplication event that generated *MeGI* and proto *OGI* (pink outlined circle) postdated the *Dd-* $\alpha$ whole-genome duplication event (or concurrent segmental duplication) which produced the *MeGI* and *SiMeGI* pair (gray filled circle). This result is well supported (bootstrap 99/100). Later, the inverted repeat of *OGI* was probably generated by local inversion of the proto *OGI* (blue outlined circle, bootstrap = 92/100). FR: forward repeat, IR: inverted repeat. Achn210611 from *Actinidia chinensis* was used as the outgroup. Divergence of Achn210611 from the *MeGI/SiMeGI* family predated the *Dd-* $\alpha$ (Figure 4b). This topology was constructed with MEGA v6 using the maximum likelihood method (GTR+I+G, gamma=3, complete deletion).

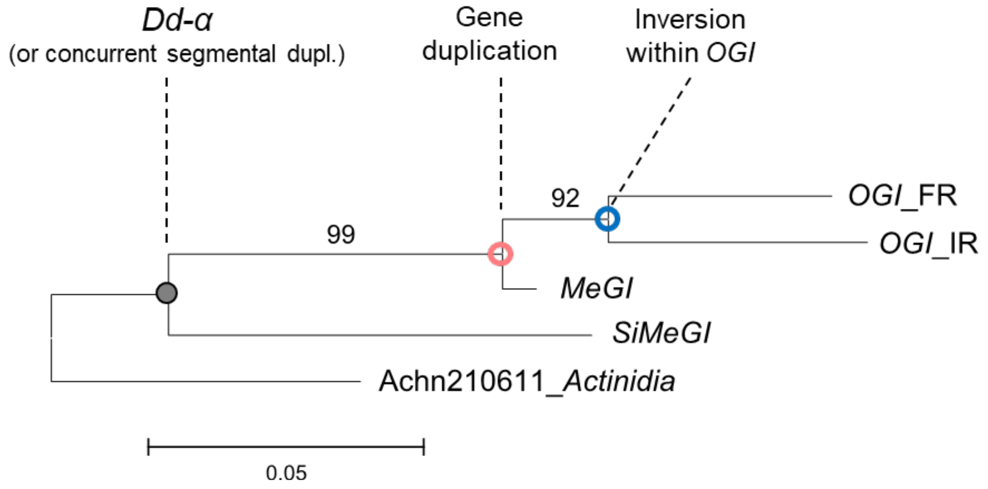

Supplement: S15 Fig — The nucleotide sequences of SiMeGI, MeGI, and the forward and inverted repeats of OGI were aligned to each other to estimate their relative timing of establishment. Our results indicated that the gene duplication event that generated MeGI and proto OGI (pink outlined circle) postdated the Dd-α whole-genome duplication event (or concurrent segmental duplication) which produced the MeGI and SiMeGI pair (gray filled circle). This result is well supported (bootstrap 99/100). Later, the inverted repeat of OGI was probably generated by local inversion of the proto OGI (blue outlined circle, bootstrap = 92/100). FR: forward repeat, IR: inverted repeat. Achn210611 from Actinidia chinensis was used as the outgroup. Divergence of Achn210611 from the MeGI/SiMeGI family predated the Dd-α (Fig 4B). This topology was constructed with MEGA v6 using the maximum likelihood method (GTR+I+G, gamma = 3, complete deletion). (PDF) [file pgen.1008566.s015.pdf]
