# Supplementary material for: The persimmon genome reveals clues to the evolution of a lineage-specific sex determination system in plants
Source: PLoS Genet. 2020 Feb 18;16(2):e1008566. doi: 10.1371/journal.pgen.1008566 (PMC7048303; doi:10.1371/journal.pgen.1008566)
Supplement: S1 Table — (PDF) [file pgen.1008566.s016.pdf]

S1 Table: Summary statistics for the initial genome assembly of *D. lotus* cv. Kunsenshi-male

		Diospyros lotus (DLO_r1.0)	*Diospyros lotus* (DLO_r1.0p)	*Diospyros lotus* (DLO_r1.0a)
Estimated genome size		-	877.7 Mb	-
Total	Number of sequences	8,974	3,073	5,901
	Total length (bp)	945,625,305	746,093,016	199,532,289
	Average length (bp)	105,374	242,790	33,813
	Max length (bp)	7,107,382	7,107,382	505,402
	Min length (bp)	2,483	2,483	2,685
	N50 length (bp)	653,828	1,060,344	43,117
	A	300,991,439	237,381,743	63,609,696
	T	300,899,593	237,282,679	63,616,914
	G	171,753,801	135,616,131	36,137,670
	C	171,980,472	135,812,463	36,168,009
	n	0	0	0
	N	0	0	0
	others	0	0	0
	Total(ACGT)	945,625,305	746,093,016	199,532,289
	GC%(ACGT)	36.3	36	36
	N%	0.0	0	0
	(Estimated size-Total(ATGC)/Estimated size	-	15.0	-
≥5 kbp	Number of sequences	8,805	3,021	5,784
	Total length (bp)	944,956,722	745,890,657	199,066,065
	Average length (bp)	107,320	246,902	34,417
Estimated genome size		-	877,700,000	-
