# Supplementary material for: The persimmon genome reveals clues to the evolution of a lineage-specific sex determination system in plants
Source: PLoS Genet. 2020 Feb 18;16(2):e1008566. doi: 10.1371/journal.pgen.1008566 (PMC7048303; doi:10.1371/journal.pgen.1008566)
Supplement: S2 Table — Genetic maps for the four parental lines of the two mapping populations (KK and VM), were built using the pseudo-test cross method. All linkage groups were anchored to the 15 chromosomes of the D. lotus draft genome assembly. The sex-determinant locus was mapped to linkage group 15, suggesting the Dlo15 is the sex chromosome. More detailed information about the maps and SNPs are available from the Persimmon Genome Database (http://persimmon.kazusa.or.jp) (PDF) [file pgen.1008566.s017.pdf]

S2 Table: Number of SNPs and length of genetic linkage maps in *D. lotus*

Genetic maps for the four parental lines of the two mapping populations (KK and VM), were built using the pseudo-test cross method. All linkage groups were anchored to the 15 chromosomes of the *D. lotus* draft genome assembly. The sex-determinant locus was mapped to linkage group 15, suggesting the Dlo15 is the sex chromosome. More detailed information about the maps and SNPs are available from the Persimmon Genome Database (http://persimmon.kazusa.or.jp)

Chromsome name	Consensus map		KK genetic map				VM genetic map			
			Kunseishi-female		Kunseishi-male		Kunseishi-female		Budogaki-male	
	No. of SNPs	Map length (cM)	No. of SNPs	Map length (cM)	No. of SNPs	Map length (cM)	No. of SNPs	Map length (cM)	No. of SNPs	Map length (cM)
Dlo01	435	223.8	140	218.4	204	208.5	154	176.0	144	110.6
Dlo02	723	132.4	307	157.9	175	162.8	148	183.1	316	132.1
Dlo03	675	63.9	236	134.4	222	219.4	238	177.4	311	166.8
Dlo04	385	159.2	116	145.1	176	172.5	174	138.6	168	139.4
Dlo05	384	136.4	106	103.7	180	159.5	191	191.5	133	136.4
Dlo06	384	139.7	75	82.6	160	159.0	159	136.0	216	129.7
Dlo07	262	139.6	97	122.3	124	170.7	81	97.7	94	114.2
Dlo08	248	127.3	62	125.5	108	160.4	108	195.0	114	135.7
Dlo09	279	99.6	58	105.9	103	158.5	115	110.1	155	115.2
Dlo10	287	105.5	103	104.4	96	89.4	74	85.7	155	124.4
Dlo11	395	147.1	150	147.1	96	163.1	35	85.6	213	160.7
Dlo12	549	126.4	286	105.9	149	156.5	168	194.8	164	79.9
Dlo13	187	49.5	36	139.2	84	133.4	53	77.2	109	99.4
Dlo14	310	116.4	67	116.4	58	98.1	57	181.0	208	169.1
Dlo15[a]	456	146.5	148	121.7	185	138.4	202	166.2	189	129.0
Total	5,959	1,913.2	1,987	1,930.6	2,120	2,350.2	1,957	2,195.7	2,689	1,942.5

[a]Putative *D. lotus* sex chromosome, including the sex-determinant locus.
