# Supplementary material for: The persimmon genome reveals clues to the evolution of a lineage-specific sex determination system in plants
Source: PLoS Genet. 2020 Feb 18;16(2):e1008566. doi: 10.1371/journal.pgen.1008566 (PMC7048303; doi:10.1371/journal.pgen.1008566)
Supplement: S3 Table — SNPs and indels were identified from whole-genome resequencing analysis of female and male lines of D. lotus, and functionally annotated and classified into four categories predefined by SnpEff (Cingolani et al., 2012): high (e.g. nonsense mutations and frameshift mutations)-, moderate (e.g. missense mutations)-, modifier (e.g. intron and intergenic mutations)- and low-impact (e.g. synonymous mutations) mutations (see http://snpeff.sourceforge.net for details). Further details about these SNPs and Indels are available from the Persimmon Genome Database (http://persimmon.kazusa.or.jp) (PDF) [file pgen.1008566.s018.pdf]

S3 Table: Number of annotated SNPs and indels between the female and male lines of *D. lotus* 'Kunsenshi'

SNPs and indels were identified from whole-genome resequencing analysis of female and male lines of *D. lotus*, and functionally annotated and classified into four categories predefined by SnpEff (Cingolani et al., 2012): high (e.g. nonsense mutations and frameshift mutations)-, moderate (e.g. missense mutations)-, modifier (e.g. intron and intergenic mutations)- and low-impact (e.g. synonymous mutations) mutations (see http://snpeff.sourceforge.net for details). Further details about these SNPs and Indels are available from the Persimmon Genome Database (http://persimmon.kazusa.or.jp).

Annotation	Impact	#Variants	%
Stop gained	HIGH	705	>0.0%
Frameshift variant	HIGH	641	>0.0%
Stop lost & splice region variant	HIGH	184	>0.0%
Splice donor variant & intron variant	HIGH	150	>0.0%
Splice acceptor variant & intron variant	HIGH	149	>0.0%
Start lost	HIGH	106	>0.0%
Frameshift variant & splice region variant	HIGH	33	>0.0%
Frameshift variant & start lost	HIGH	23	>0.0%
Splice donor variant & splice region variant & intron variant	HIGH	23	>0.0%
Stop gained & inframe insertion	HIGH	17	>0.0%
Frameshift variant & stop lost & splice region variant	HIGH	16	>0.0%
Stop gained & splice region variant	HIGH	16	>0.0%
Frameshift variant & stop gained	HIGH	11	>0.0%
Stop gained & disruptive inframe insertion	HIGH	8	>0.0%
Splice acceptor variant & splice region variant & intron variant	HIGH	3	>0.0%
Stop lost & inframe deletion & splice region variant	HIGH	2	>0.0%
Frameshift variant & splice acceptor variant & splice region variant & intron variant	HIGH	1	>0.0%
Frameshift variant & splice donor variant & splice region variant & intron variant	HIGH	1	>0.0%
Start lost & disruptive inframe insertion	HIGH	1	>0.0%
Stop gained & disruptive inframe deletion	HIGH	1	>0.0%
Subtotal of high impact variants		2,091	0.1%
Missense variant	MODERATE	27,843	1.6%
Inframe insertion	MODERATE	404	>0.0%
Missense variant & splice region variant	MODERATE	377	>0.0%
Inframe deletion	MODERATE	199	>0.0%
Disruptive inframe insertion	MODERATE	191	>0.0%
Disruptive inframe deletion	MODERATE	114	>0.0%
Inframe insertion & splice region variant	MODERATE	7	>0.0%
Disruptive inframe insertion & splice region variant	MODERATE	5	>0.0%
Disruptive inframe deletion & splice region variant	MODERATE	1	>0.0%
Subtotal of moderate impact variants		29,141	1.7%
Intergenic region	MODIFIER	1,424,741	82.1%
Intron variant	MODIFIER	223,388	12.9%
Intragenic variant	MODIFIER	22,774	1.3%
Subtotal of modifier impact variants		1,670,903	96.3%
Synonymous variant	LOW	24,127	1.4%
Splice region variant & intron variant	LOW	2,928	0.2%
Splice region variant & synonymous variant	LOW	363	>0.0%
Splice region variant & stop retained variant	LOW	64	>0.0%
Initiator codon variant	LOW	10	>0.0%
Subtotal of low impact variants		27,492	1.6%
Unassigned	-	5,656	0.3%
Total		1,735,283	100.0%
