# Supplementary material for: The persimmon genome reveals clues to the evolution of a lineage-specific sex determination system in plants
Source: PLoS Genet. 2020 Feb 18;16(2):e1008566. doi: 10.1371/journal.pgen.1008566 (PMC7048303; doi:10.1371/journal.pgen.1008566)
Supplement: S4 Table — Repetitive sequences amounted for 630.2 Mb (66.6%) of the total length of the final genome assembly. Unique repeats were abundant in the D. lotus genomes, constituting 49.8% of all repeats. Of the known types of repeats, Class I LTR elements were observed most frequently (11.2%). (PDF) [file pgen.1008566.s019.pdf]

S4 Table: Comparison of the repeat sequences in representative eudicot genomes

Repetitive sequences amounted for 630.2 Mb (66.6%) of the total length of the final genome assembly. Unique repeats were abundant in the *D. lotus* genomes, constituting 49.8% of all repeats. Of the known types of repeats, Class I LTR elements were observed most frequently (11.2%).

					Diospyros lotus (DLO_r1.0)		*Diospyros lotus* (DLO_r1.0p)		*Diospyros lotus* (DLO_r1.0a)		*Actinidia chinensis*		*Solanum lycopersicum* (SL3.0)	
					Length occupied (bp)	% of whole genome	Length occupied (bp)	% of whole genome	Length occupied (bp)	% of whole genome	Length occupied (bp)	% of whole genome	Length occupied (bp)	% of whole genome
Known repeats	Interspersed repeats	Class I	SINEs	Total	166,838	0.0	115,389.0	0.0	51,449.0	0.0	147,040	0.0	1,180,093	0.1
			LINEs	Total	7,806,418	0.8	6,073,244.0	0.8	1,733,174.0	0.9	1,968,880	0.3	10,156,031	1.2
			LTR elements	Total	106,069,966	11.2	83,669,799.0	11.2	22,400,167.0	11.2	37,945,569	6.0	268,611,657	32.4
				Copia	57,552,037	6.1	45,352,029.0	6.1	12,200,008.0	6.1	20,917,900	3.3	43,976,597	5.3
				Gypsy	46,703,277	4.9	36,947,330.0	5.0	9,755,947.0	4.9	15,343,487	2.4	219,399,447	26.5
		Class II	DNA elements		11,794,037	1.2	9,031,535.0	1.2	2,762,502.0	1.4	5,578,239	0.9	26,792,666	3.2
		Unclassified			614	0.0	614.0	0.0	0.0	0.0	119	0.0	2,123,585	0.3
	Helitrons				1,001,867	0.1	768,779.0	0.1	233,088.0	0.1	379,702	0.1	538,934	0.1
	Low complexity				7,064,999	0.7	5,553,583.0	0.7	1,511,416.0	0.8	4,158,182	0.7	2,158,535	0.3
	Simple repeat				22,555,879	2.4	17,650,019.0	2.4	4,905,860.0	2.5	12,809,197	2.0	9,387,824	1.1
	Unknown				40,997	0.0	28,463.0	0.0	12,534.0	0.0	33,884	0.0	18,192	0.0
	Subtotal				159,346,866	16.9	125,065,495.0	16.8	34,281,371.0	17.2	64,988,969	10.3	326,352,654	39.4
Unique repeats	Unknown				470,242,683	49.7	371,663,642.0	49.8	98,579,041.0	49.4	182,375,737	28.9	212,578,211	25.7
	Simple repeat				652,914	0.1	507,792.0	0.1	145,122.0	0.1	231,950	0.0	197,855	0.0
	Subtotal				470,895,597	49.8	372,171,434.0	49.9	98,724,163.0	49.5	182,607,687	28.9	212,776,066	25.7
							497,236,929.0	66.6	133,005,534.0	66.7	247,596,656	39.2	539,128,720	65.1

Lactuca sativa (V8)		*Vitis vinifera* (IGGP 12x.31)		*Prunus persica* (v2.0.a1)		*Carica papaya* (ASGPBv0.4)	
Length occupied (bp)	% of whole genome	Length occupied (bp)	% of whole genome	Length occupied (bp)	% of whole genome	Length occupied (bp)	% of whole genome
8,299	0.0	44,308	0.0	13,945	0.0	4,507	0.0
1,000,875	0.0	19,332,776	4.0	1,047,154	0.5	5,273,047	1.5
243,368,347	10.5	110,004,991	22.6	21,152,416	9.3	72,920,228	21.3
105,761,119	4.6	40,155,666	8.3	9,297,257	4.1	13,133,896	3.8
132,932,224	5.8	66,806,791	13.7	10,567,311	4.6	59,336,577	17.3
7,603,516	0.3	27,130,274	5.6	9,021,688	4.0	1,355,568	0.4
725	0.0	97	0.0	113	0.0	0	0.0
1,362,310	0.1	621,626	0.1	508,703	0.2	103,304	0.0
6,644,298	0.3	2,548,079	0.5	1,114,697	0.5	1,305,362	0.4
34,623,944	1.5	7,999,159	1.6	3,966,247	1.7	5,147,395	1.5
18,104	0.0	9,894	0.0	42,885	0.0	15,187	0.0
301,067,075	13.0	170,906,209	35.1	38,190,482	16.8	86,709,638	25.3
1,349,479,454	58.4	97,889,702	20.1	65,064,360	28.6	30,037,776	8.8
353,594	0.0	307,310	0.1	31,221	0.0	171,095	0.0
1,349,833,048	58.4	98,197,012	20.2	65,095,581	28.6	30,208,871	8.8

Arabidopsis thaliana (TAIR10)	
Length occupied (bp)	% of whole genome
123,259	0.1
1,336,827	1.1
8,154,990	6.8
1,818,720	1.5
6,224,830	5.2
5,585,341	4.7
0	0.0
2,173,124	1.8
406,058	0.3
1,314,108	1.1
6,400	0.0
19,220,552	16.1
4,935,265	4.1
10,315	0.0
4,945,580	4.1
24,166,132	20.2
