# Supplementary material for: The persimmon genome reveals clues to the evolution of a lineage-specific sex determination system in plants
Source: PLoS Genet. 2020 Feb 18;16(2):e1008566. doi: 10.1371/journal.pgen.1008566 (PMC7048303; doi:10.1371/journal.pgen.1008566)
Supplement: S5 Table — (PDF) [file pgen.1008566.s020.pdf]

## S5 Table: Phenotypic characterization of the p35S-*MeGI N. tabacum* transformed lines.

| T1 Line ID | introduced construct | feminization[a] | narrow leaves[b] | dwarfisms[c] | transgene expression in flowers |
|---|---|---|---|---|---|
| Nita-p35S-MeGI-1 | pGWB2-MeGI | no flowers | ++ | ++ | + |
| Nita-p35S-MeGI-2 | pGWB2-MeGI | – | – | – | + |
| Nita-p35S-MeGI-3 | pGWB2-MeGI | + | + | – | + |
| Nita-p35S-MeGI-4 | pGWB2-MeGI | + | + | + | + |
| Nita-p35S-MeGI-5 | pGWB2-MeGI | + | – | – | + |
| Nita-p35S-MeGI-6 | pGWB2-MeGI | + | + | – | + |
| Nita-p35S-MeGI-7 | pGWB2-MeGI | + | + | + | + |
| Nita-p35S-MeGI-8 | pGWB2-MeGI | – | – | – | + |
| Nita-p35S-MeGI-9 | pGWB2-MeGI | + | – | – | + |
| Nita-p35S-MeGI-10 | pGWB2-MeGI | – | – | – | – |
| Nita-p35S-MeGI-11 | pGWB2-MeGI | – | + | – | + |
| Nita-p35S-MeGI-12 | pGWB2-MeGI | no flowers | ++ | ++ | + |
| Nita-p35S-MeGI-13 | pGWB2-MeGI | – | + | – | + |
| Nita-p35S-MeGI-14 | pGWB2-MeGI | + | – | – | + |
| Nita-p35S-MeGI-15 | pGWB2-MeGI | + | – | – | + |
| Nita-p35S-MeGI-16 | pGWB2-MeGI | + | – | – | + |
| Nita-p35S-MeGI-17 | pGWB2-MeGI | + | + | + | + |
| Nita-p35S-MeGI-18 | pGWB2-MeGI | no flowers | ++ | ++ | + |
| Nita-p35S-MeGI-19 | pGWB2-MeGI | + | + | + | + |
| Nita-p35S-MeGI-20 | pGWB2-MeGI | – | + | + | + |
| Nita-p35S-MeGI-21 | pGWB2-MeGI | – | – | – | + |
| Nita-p35S-MeGI-22 | pGWB2-MeGI | + | ++ | + | + |
| Nita-p35S-MeGI-23 | pGWB2-MeGI | + | ++ | + | + |

[a] "+" indicates feminization.
[b] "+" indicates narrow leaves, as shown in Figure 5a and Figure S7.
[c] "+" and "++" indicate semi-dwarfing and dwarfing phenotypes, respectively, as shown in Figure 5a.
