# Supplementary material for: The persimmon genome reveals clues to the evolution of a lineage-specific sex determination system in plants
Source: PLoS Genet. 2020 Feb 18;16(2):e1008566. doi: 10.1371/journal.pgen.1008566 (PMC7048303; doi:10.1371/journal.pgen.1008566)
Supplement: S6 Table — (PDF) [file pgen.1008566.s021.pdf]

S6 Table: Phenotypic characterization of the p35S-*SiMeGI N. tabacum* transformed lines.

T1 Line ID	introduced construct	feminization[a]	narrow leaves[b]	dwarfisms[c]	transgene expression in flowers
Nita-p35S-SiMeGI-1	pGWB2-SiMeGI	–	–	+	+
Nita-p35S-SiMeGI-2	pGWB2-SiMeGI	–	–	+	+
Nita-p35S-SiMeGI-3	pGWB2-SiMeGI	–	–	–	–
Nita-p35S-SiMeGI-4	pGWB2-SiMeGI	–	–	–	+
Nita-p35S-SiMeGI-5	pGWB2-SiMeGI	–	–	+	+
Nita-p35S-SiMeGI-6	pGWB2-SiMeGI	–	–	+	+
Nita-p35S-SiMeGI-7	pGWB2-SiMeGI	–	–	–	+
Nita-p35S-SiMeGI-8	pGWB2-SiMeGI	–	–	–	+
Nita-p35S-SiMeGI-9	pGWB2-SiMeGI	–	–	–	+
Nita-p35S-SiMeGI-10	pGWB2-SiMeGI	–	–	–	+
Nita-p35S-SiMeGI-11	pGWB2-SiMeGI	–	–	–	+
Nita-p35S-SiMeGI-12	pGWB2-SiMeGI	–	–	+	+
Nita-p35S-SiMeGI-13	pGWB2-SiMeGI	–	–	–	+
Nita-p35S-SiMeGI-14	pGWB2-SiMeGI	–	–	+	+
Nita-p35S-SiMeGI-15	pGWB2-SiMeGI	–	–	–	+
Nita-p35S-SiMeGI-16	pGWB2-SiMeGI	–	–	–	+
Nita-p35S-SiMeGI-17	pGWB2-SiMeGI	–	–	–	–
Nita-p35S-SiMeGI-18	pGWB2-SiMeGI	–	–	+	+
Nita-p35S-SiMeGI-19	pGWB2-SiMeGI	–	–	+	+
Nita-p35S-SiMeGI-20	pGWB2-SiMeGI	–	–	+	+

[a] "+" indicates feminization.
[b] "+" indicates narrow leaves, as shown in Figure 5.
[c] "+" indicates semi-dwarf phenotype, as shown in Figure 5.
