# Supplementary material for: The persimmon genome reveals clues to the evolution of a lineage-specific sex determination system in plants
Source: PLoS Genet. 2020 Feb 18;16(2):e1008566. doi: 10.1371/journal.pgen.1008566 (PMC7048303; doi:10.1371/journal.pgen.1008566)
Supplement: S7 Table — (PDF) [file pgen.1008566.s022.pdf]

S7 Table: Phenotypic characterization of the p35S-*MeGI A. thaliana* transformed lines.

T1 Line ID	introduced construct	feminization[a]	dwarfism[b]	transgene expression in whole plant[c]
Arth-p35S-MeGI-1	pGWB2-MeGI	+	++	++
Arth-p35S-MeGI-2	pGWB2-MeGI	+	+	++
Arth-p35S-MeGI-3	pGWB2-MeGI	–	+	+
Arth-p35S-MeGI-4	pGWB2-MeGI	–	+	+
Arth-p35S-MeGI-5	pGWB2-MeGI	–	–	+
Arth-p35S-MeGI-6	pGWB2-MeGI	–	–	–
Arth-p35S-MeGI-7	pGWB2-MeGI	–	–	+
Arth-p35S-MeGI-8	pGWB2-MeGI	–	–	+
Arth-p35S-MeGI-9	pGWB2-MeGI	–	–	+
Arth-p35S-MeGI-10	pGWB2-MeGI	–	–	+
Arth-p35S-MeGI-11	pGWB2-MeGI	+	+	++
Arth-p35S-MeGI-12	pGWB2-MeGI	–	+	+
Arth-p35S-MeGI-13	pGWB2-MeGI	–	+	+
Arth-p35S-MeGI-14	pGWB2-MeGI	–	–	+
Arth-p35S-MeGI-15	pGWB2-MeGI	–	–	+
Arth-p35S-MeGI-16	pGWB2-MeGI	–	–	–
Arth-p35S-MeGI-17	pGWB2-MeGI	–	+	NA
Arth-p35S-MeGI-18	pGWB2-MeGI	+	++	NA
Arth-p35S-MeGI-19	pGWB2-MeGI	+	+	NA
Arth-p35S-MeGI-20	pGWB2-MeGI	+	+	NA
Arth-p35S-MeGI-21	pGWB2-MeGI	–	–	NA
Arth-p35S-MeGI-22	pGWB2-MeGI	+	++	NA
Arth-p35S-MeGI-23	pGWB2-MeGI	+	++	NA
Arth-p35S-MeGI-24	pGWB2-MeGI	–	–	NA
Arth-p35S-MeGI-25	pGWB2-MeGI	–	–	NA
Arth-p35S-MeGI-26	pGWB2-MeGI	–	–	NA
Arth-p35S-MeGI-27	pGWB2-MeGI	–	+	NA
Arth-p35S-MeGI-28	pGWB2-MeGI	–	+	NA
Arth-p35S-MeGI-29	pGWB2-MeGI	–	+	NA
Arth-p35S-MeGI-30	pGWB2-MeGI	–	–	NA
Arth-p35S-MeGI-31	pGWB2-MeGI	–	+	NA
Arth-p35S-MeGI-32	pGWB2-MeGI	–	+	NA
Arth-p35S-MeGI-33	pGWB2-MeGI	–	–	NA
Arth-p35S-MeGI-34	pGWB2-MeGI	–	–	NA
Arth-p35S-MeGI-35	pGWB2-MeGI	–	+	NA
Arth-p35S-MeGI-36	pGWB2-MeGI	–	–	NA
Arth-p35S-MeGI-37	pGWB2-MeGI	–	–	NA
Arth-p35S-MeGI-38	pGWB2-MeGI	–	–	NA

Arth-p35S-MeGI-39	pGWB2-MeGI	+	+	NA
Arth-p35S-MeGI-40	pGWB2-MeGI	+	+	NA
Arth-p35S-MeGI-41	pGWB2-MeGI	+	+	NA
Arth-p35S-MeGI-42	pGWB2-MeGI	–	–	NA
Arth-p35S-MeGI-43	pGWB2-MeGI	–	–	NA
Arth-p35S-MeGI-44	pGWB2-MeGI	–	–	NA
Arth-p35S-MeGI-45	pGWB2-MeGI	–	+	NA
Arth-p35S-MeGI-46	pGWB2-MeGI	–	–	NA
Arth-p35S-MeGI-47	pGWB2-MeGI	–	+	NA
Arth-p35S-MeGI-48	pGWB2-MeGI	+	–	NA
Arth-p35S-MeGI-49	pGWB2-MeGI	–	–	NA
Arth-p35S-MeGI-50	pGWB2-MeGI	–	–	NA
Arth-p35S-MeGI-51	pGWB2-MeGI	–	+	NA
Arth-p35S-MeGI-52	pGWB2-MeGI	–	–	NA
Arth-p35S-MeGI-53	pGWB2-MeGI	–	–	NA
Arth-p35S-MeGI-54	pGWB2-MeGI	–	–	NA
Arth-p35S-MeGI-55	pGWB2-MeGI	–	–	NA
Arth-p35S-MeGI-56	pGWB2-MeGI	+	+	NA
Arth-p35S-MeGI-57	pGWB2-MeGI	–	–	NA
Arth-p35S-MeGI-58	pGWB2-MeGI	–	–	NA
Arth-p35S-MeGI-59	pGWB2-MeGI	–	–	NA
Arth-p35S-MeGI-60	pGWB2-MeGI	–	–	NA
Arth-p35S-MeGI-61	pGWB2-MeGI	–	–	NA
Arth-p35S-MeGI-62	pGWB2-MeGI	–	+	NA
Arth-p35S-MeGI-63	pGWB2-MeGI	+	++	NA
Arth-p35S-MeGI-64	pGWB2-MeGI	–	–	NA
Arth-p35S-MeGI-65	pGWB2-MeGI	–	–	NA
Arth-p35S-MeGI-66	pGWB2-MeGI	–	–	NA
Arth-p35S-MeGI-67	pGWB2-MeGI	–	+	NA
Arth-p35S-MeGI-68	pGWB2-MeGI	–	+	NA
Arth-p35S-MeGI-69	pGWB2-MeGI	–	+	NA

[a] "+" indicates feminization.
[b] "++" and "+" indicate dwarfing and semi-dwarfing, respectively (see Figure S8).
[c] Expression levels were assessed in transgenic 16 individuals by RT-PCR analysis.
