# Supplementary material for: The persimmon genome reveals clues to the evolution of a lineage-specific sex determination system in plants
Source: PLoS Genet. 2020 Feb 18;16(2):e1008566. doi: 10.1371/journal.pgen.1008566 (PMC7048303; doi:10.1371/journal.pgen.1008566)
Supplement: S8 Table — (PDF) [file pgen.1008566.s023.pdf]

S8 Table: Phenotypic characterization of the p35S-*SiMeGI A. thaliana* transformed lines.

T1 Line ID	introduced construct	feminization[a]	dwarfism[b]	transgene expression in whole plant[c]
Arth-35S-SiMeGI-1	pGWB2-MeGI	–	–	+
Arth-35S-SiMeGI-2	pGWB2-MeGI	–	–	++
Arth-35S-SiMeGI-3	pGWB2-MeGI	–	+	++
Arth-35S-SiMeGI-4	pGWB2-MeGI	–	–	++
Arth-35S-SiMeGI-5	pGWB2-MeGI	–	+	+
Arth-35S-SiMeGI-6	pGWB2-MeGI	–	–	+
Arth-35S-SiMeGI-7	pGWB2-MeGI	–	+	++
Arth-35S-SiMeGI-8	pGWB2-MeGI	–	–	++
Arth-35S-SiMeGI-9	pGWB2-MeGI	–	–	+
Arth-35S-SiMeGI-10	pGWB2-MeGI	–	–	+
Arth-35S-SiMeGI-11	pGWB2-MeGI	–	–	++
Arth-35S-SiMeGI-12	pGWB2-MeGI	+	+	++
Arth-35S-SiMeGI-13	pGWB2-MeGI	–	–	+
Arth-35S-SiMeGI-14	pGWB2-MeGI	–	–	+
Arth-35S-SiMeGI-15	pGWB2-MeGI	–	–	+
Arth-35S-SiMeGI-16	pGWB2-MeGI	–	–	+
Arth-35S-SiMeGI-17	pGWB2-MeGI	–	–	+
Arth-35S-SiMeGI-18	pGWB2-MeGI	+	+	+
Arth-35S-SiMeGI-19	pGWB2-MeGI	–	+	+
Arth-35S-SiMeGI-20	pGWB2-MeGI	–	–	+
Arth-35S-SiMeGI-21	pGWB2-MeGI	–	–	+
Arth-35S-SiMeGI-22	pGWB2-MeGI	–	+	+
Arth-35S-SiMeGI-23	pGWB2-MeGI	–	–	+
Arth-35S-SiMeGI-24	pGWB2-MeGI	–	–	+
Arth-35S-SiMeGI-25	pGWB2-MeGI	–	+	NA
Arth-35S-SiMeGI-26	pGWB2-MeGI	–	+	NA
Arth-35S-SiMeGI-27	pGWB2-MeGI	–	–	NA
Arth-35S-SiMeGI-28	pGWB2-MeGI	–	–	NA
Arth-35S-SiMeGI-29	pGWB2-MeGI	–	–	NA
Arth-35S-SiMeGI-30	pGWB2-MeGI	–	–	NA
Arth-35S-SiMeGI-31	pGWB2-MeGI	–	–	NA
Arth-35S-SiMeGI-32	pGWB2-MeGI	–	–	NA
Arth-35S-SiMeGI-33	pGWB2-MeGI	–	–	NA
Arth-35S-SiMeGI-34	pGWB2-MeGI	–	–	NA
Arth-35S-SiMeGI-35	pGWB2-MeGI	–	–	NA
Arth-35S-SiMeGI-36	pGWB2-MeGI	–	–	NA
Arth-35S-SiMeGI-37	pGWB2-MeGI	–	+	NA
Arth-35S-SiMeGI-38	pGWB2-MeGI	–	–	NA
Arth-35S-SiMeGI-39	pGWB2-MeGI	–	–	NA

Arth-35S-SiMeGI-40	pGWB2-MeGI	–	–	NA
Arth-35S-SiMeGI-41	pGWB2-MeGI	–	–	NA
Arth-35S-SiMeGI-42	pGWB2-MeGI	–	–	NA
Arth-35S-SiMeGI-43	pGWB2-MeGI	–	+	NA
Arth-35S-SiMeGI-44	pGWB2-MeGI	–	+	NA
Arth-35S-SiMeGI-45	pGWB2-MeGI	–	–	NA
Arth-35S-SiMeGI-46	pGWB2-MeGI	–	+	NA
Arth-35S-SiMeGI-47	pGWB2-MeGI	–	+	NA
Arth-35S-SiMeGI-48	pGWB2-MeGI	–	–	NA
Arth-35S-SiMeGI-49	pGWB2-MeGI	–	–	NA
Arth-35S-SiMeGI-50	pGWB2-MeGI	–	–	NA
Arth-35S-SiMeGI-51	pGWB2-MeGI	–	–	NA
Arth-35S-SiMeGI-52	pGWB2-MeGI	–	+	NA
Arth-35S-SiMeGI-53	pGWB2-MeGI	–	–	NA

[a] "+" indicates feminization.

[b] "++" and "+" indicate dwarfing and semi-dwarfing, respectively (see Figure S8).

[c] Expression levels were assessed in 16 transgenic individuals by RT-PCR analysis.
