# Supplementary material for: The persimmon genome reveals clues to the evolution of a lineage-specific sex determination system in plants
Source: PLoS Genet. 2020 Feb 18;16(2):e1008566. doi: 10.1371/journal.pgen.1008566 (PMC7048303; doi:10.1371/journal.pgen.1008566)
Supplement: S9 Table — (PDF) [file pgen.1008566.s024.pdf]

S9 Table. Anchoring of chromosome 15 using sex-linked (Y-allelic) SNPs markers

Population-parent for genotyping	Chromosome	Map position (cM)	Locus ID
KK-KM (male)	15	66.496	Dlo_pri0160F.1_727544
KK-KM (male)	15	67.276	Dlo_pri0160F.1_1074837
KK-KM (male)	15	68.378	Dlo_pri0203F.1_905497
KK-KM (male)	15	68.716	Dlo_pri0203F.1_896639
KK-KM (male)	15	71.38	Dlo_pri0203F.1_490726
KK-KM (male)	15	73.084	Dlo_pri0188F.1_603265
KK-KM (male)	15	73.667	Dlo_pri0188F.1_998814
KK-KM (male)	15	74.663	Dlo_pri0188F.1_236736
KK-KM (male)	15	76.082	Dlo_pri0114F.1_1338478
KK-KM (male)	15	78.253	Dlo_pri0114F.1_592741
KK-KM (male)	15	78.253	Dlo_pri0114F.1_592830
KK-KM (male)	15	78.491	Dlo_pri0114F.1_629206
KK-KM (male)	15	79.405	Dlo_pri0114F.1_173263
KK-KM (male)	15	80.063	Dlo_pri0114F.1_239874
male-specific region of the Y-chromosome			
KK-KM (male)	15	82.851	Dlo_pri0086F.1_1819467
KK-KM (male)	15	83.102	Dlo_pri0086F.1_1809656
KK-KM (male)	15	83.102	Dlo_pri0086F.1_1809659
KK-KM (male)	15	87.33	Dlo_pri0017F.1_2455031
KK-KM (male)	15	88.137	Dlo_pri0017F.1_2484613
KK-KM (male)	15	88.137	Dlo_pri0017F.1_2484662
KK-KM (male)	15	89.498	Dlo_pri0017F.1_2593614
KK-KM (male)	15	92.698	Dlo_pri0017F.1_3147652
KK-KM (male)	15	93.131	Dlo_pri0017F.1_3023457
KK-KM (male)	15	97.007	Dlo_pri0240F-1.1_227083
KK-KM (male)	15	100.202	Dlo_pri0137F.1_1186429
KK-KM (male)	15	101.561	Dlo_pri0137F.1_1006104
KK-KM (male)	15	101.561	Dlo_pri0137F.1_1006110
KK-KM (male)	15	101.844	Dlo_pri0137F.1_1006146
