# Supplementary material for: The persimmon genome reveals clues to the evolution of a lineage-specific sex determination system in plants
Source: PLoS Genet. 2020 Feb 18;16(2):e1008566. doi: 10.1371/journal.pgen.1008566 (PMC7048303; doi:10.1371/journal.pgen.1008566)
Supplement: S10 Table — (PDF) [file pgen.1008566.s025.pdf]

S10 Table: Plant materials

name	Gender[a]	Generation/population	Assessment[b]	Cross (year)
Kunsenshi-male	Male	Male parent of the KK amd VM populations	RAD/GBS, draft genome sequencing, transcriptome	
Kunsenshi-female	Female	Female parent of the KK population	RAD/GBS, transcriptome	
Budogaki	Female	Female parent of the VM population	RAD/na	
KK1_001	Male	F1, KK population	RAD/GBS	2004
KK1_002	Male	F1, KK population	RAD/GBS	2004
KK1_003	Male	F1, KK population	RAD/GBS	2004
KK1_004	Male	F1, KK population	RAD/GBS	2004
KK1_005	Male	F1, KK population	RAD/na	2004
KK1_006	Male	F1, KK population	RAD/GBS	2004
KK1_007	Female	F1, KK population	na/GBS	2004
KK1_009	Male	F1, KK population	RAD/GBS	2004
KK1_010	Female	F1, KK population	RAD/GBS	2004
KK1_011	Female	F1, KK population	RAD/GBS	2004
KK1_012	Male	F1, KK population	RAD/GBS	2004
KK1_013	Female	F1, KK population	RAD/na	2004
KK1_014	Female	F1, KK population	RAD/GBS	2004
KK1_016	Female	F1, KK population	RAD/GBS	2004
KK1_017	Female	F1, KK population	RAD/GBS	2004
KK1_018	Female	F1, KK population	RAD/na	2004
KK1_020	Female	F1, KK population	RAD/GBS	2004
KK1_021	Male	F1, KK population	RAD/na	2004
KK1_022	Male	F1, KK population	RAD/GBS	2004
KK1_023	Female	F1, KK population	RAD/GBS	2004
KK1_024	Female	F1, KK population	RAD/GBS	2004
KK1_025	Male	F1, KK population	RAD/GBS	2004
KK1_027	Female	F1, KK population	RAD/GBS	2004
KK1_028	Male	F1, KK population	RAD/GBS	2004
KK1_029	Female	F1, KK population	RAD/GBS	2004
KK1_030	Female	F1, KK population	na/GBS	2004
KK1_031	Male	F1, KK population	RAD/GBS	2004
KK1_032	Male	F1, KK population	RAD/GBS	2004
KK1_033	Female	F1, KK population	RAD/GBS	2004
KK1_034	Male	F1, KK population	RAD/GBS	2004
KK1_035	Female	F1, KK population	RAD/GBS	2004
KK1_036	Male	F1, KK population	RAD/GBS	2004
KK1_037	Female	F1, KK population	na/GBS	2004
KK1_038	Female	F1, KK population	RAD/GBS	2004
KK1_039	Female	F1, KK population	RAD/GBS	2004
KK1_040	Male	F1, KK population	RAD/GBS	2004
KK1_041	Female	F1, KK population	RAD/GBS	2004
KK1_042	Female	F1, KK population	RAD/na	2004
KK1_043	Male	F1, KK population	RAD/na	2004
KK1_044	Female	F1, KK population	RAD/na	2004
KK1_045	Female	F1, KK population	RAD/na	2004
KK1_046	Female	F1, KK population	RAD/GBS	2004
KK1_047	Female	F1, KK population	RAD/na	2004
KK1_048	Male	F1, KK population	RAD/GBS	2004
KK1_049	Female	F1, KK population	RAD/GBS	2004
KK1_051	Female	F1, KK population	RAD/GBS	2004
KK1_052	Male	F1, KK population	RAD/GBS	2004
KK1_054	Female	F1, KK population	RAD/GBS	2004
KK1_055	Female	F1, KK population	RAD/na	2004
KK1_056	Female	F1, KK population	RAD/GBS	2004

KK1_057	Male	F1, KK population	RAD/na	2004
KK1_058	Female	F1, KK population	RAD/na	2004
KK1_059	Female	F1, KK population	RAD/GBS	2004
KK1_060	Female	F1, KK population	RAD/GBS	2004
KK1_061	Male	F1, KK population	RAD/GBS	2004
KK1_062	Female	F1, KK population	RAD/GBS	2004
KK1_063	Male	F1, KK population	RAD/GBS	2004
KK1_064	Female	F1, KK population	RAD/GBS	2004
KK2_001	Female	F1, KK population	RAD/na	2011
KK2_002	Female	F1, KK population	RAD/GBS	2011
KK2_003	Male	F1, KK population	RAD/na	2011
KK2_004	NF	F1, KK population	RAD/GBS	2011
KK2_005	Female	F1, KK population	RAD/GBS	2011
KK2_006	NF	F1, KK population	RAD/GBS	2011
KK2_007	Male	F1, KK population	RAD/na	2011
KK2_008	Male	F1, KK population	RAD/GBS	2011
KK2_009	Male	F1, KK population	RAD/GBS	2011
KK2_010	Female	F1, KK population	RAD/GBS	2011
KK2_011	Female	F1, KK population	RAD/GBS	2011
KK2_012	NF	F1, KK population	RAD/GBS	2011
KK2_013	Male	F1, KK population	RAD/GBS	2011
KK2_014	NF	F1, KK population	RAD/GBS	2011
KK2_015	Male	F1, KK population	RAD/GBS	2011
KK2_016	Male	F1, KK population	RAD/GBS	2011
KK2_017	NF	F1, KK population	RAD/GBS	2011
KK2_018	Female	F1, KK population	RAD/GBS	2011
KK2_019	Female	F1, KK population	RAD/GBS	2011
KK2_020	Male	F1, KK population	RAD/GBS	2011
KK2_021	NF	F1, KK population	RAD/GBS	2011
KK2_022	NF	F1, KK population	RAD/GBS	2011
KK2_023	Female	F1, KK population	RAD/na	2011
KK2_024	Female	F1, KK population	RAD/GBS	2011
KK2_025	Male	F1, KK population	RAD/GBS	2011
KK2_026	Male	F1, KK population	RAD/GBS	2011
KK2_027	Female	F1, KK population	RAD/GBS	2011
KK2_028	Female	F1, KK population	RAD/GBS	2011
KK2_029	Female	F1, KK population	RAD/GBS	2011
KK2_030	Female	F1, KK population	RAD/GBS	2011
KK2_031	Female	F1, KK population	RAD/GBS	2011
KK2_032	Female	F1, KK population	RAD/GBS	2011
KK2_033	Female	F1, KK population	RAD/GBS	2011
KK2_034	Female	F1, KK population	RAD/GBS	2011
KK2_035	Male	F1, KK population	RAD/GBS	2011
KK2_036	Female	F1, KK population	RAD/na	2011
KK2_037	Male	F1, KK population	RAD/GBS	2011
KK2_038	Female	F1, KK population	RAD/GBS	2011
KK2_039	Male	F1, KK population	RAD/GBS	2011
KK2_040	Female	F1, KK population	RAD/GBS	2011
KK2_041	Male	F1, KK population	RAD/GBS	2011
KK2_042	NF	F1, KK population	RAD/GBS	2011
KK2_043	Male	F1, KK population	RAD/GBS	2011
KK2_044	Female	F1, KK population	RAD/GBS	2011
KK2_045	NF	F1, KK population	RAD/GBS	2011
KK2_046	Female	F1, KK population	RAD/GBS	2011
KK2_047	Male	F1, KK population	RAD/GBS	2011
KK2_048	Female	F1, KK population	RAD/GBS	2011
KK2_049	Female	F1, KK population	RAD/GBS	2011
KK2_050	Female	F1, KK population	RAD/GBS	2011
KK2_051	Male	F1, KK population	RAD/GBS	2011
KK2_052	Female	F1, KK population	RAD/GBS	2011
KK2_053	Male	F1, KK population	RAD/GBS	2011
KK2_054	Female	F1, KK population	RAD/GBS	2011
KK2_055	Female	F1, KK population	RAD/GBS	2011
KK2_056	Female	F1, KK population	RAD/GBS	2011
KK2_057	Male	F1, KK population	RAD/GBS	2011

KK2_058	Male	F1, KK population	RAD/GBS	2011
KK2_059	NF	F1, KK population	RAD/GBS	2011
KK2_060	Male	F1, KK population	RAD/GBS	2011
KK2_061	Female	F1, KK population	RAD/GBS	2011
KK2_062	NF	F1, KK population	RAD/GBS	2011
KK2_063	Female	F1, KK population	RAD/GBS	2011
KK2_064	NF	F1, KK population	RAD/GBS	2011
KK2_065	NF	F1, KK population	RAD/GBS	2011
KK2_066	NF	F1, KK population	RAD/na	2011
KK2_067	NF	F1, KK population	RAD/GBS	2011
KK2_068	NF	F1, KK population	RAD/na	2011
KK2_069	NF	F1, KK population	RAD/na	2011
KK2_070	NF	F1, KK population	RAD/na	2011
KK2_071	Male	F1, KK population	RAD/GBS	2011
KK2_072	Male	F1, KK population	RAD/na	2011
KK2_073	Male	F1, KK population	RAD/na	2011
KK2_074	Male	F1, KK population	RAD/GBS	2011
KK2_075	Female	F1, KK population	RAD/na	2011
KK2_076	Male	F1, KK population	RAD/GBS	2011
KK2_077	NF	F1, KK population	RAD/GBS	2011
KK2_078	Female	F1, KK population	RAD/GBS	2011
KK2_079	Female	F1, KK population	RAD/GBS	2011
KK2_080	NF	F1, KK population	RAD/GBS	2011
KK2_081	NF	F1, KK population	RAD/GBS	2011
KK2_085	Male	F1, KK population	na/GBS	2011
KK2_086	Male	F1, KK population	na/GBS	2011
KK2_088	Male	F1, KK population	na/GBS	2011
KK2_089	Female	F1, KK population	na/GBS	2011
KK2_090	Male	F1, KK population	na/GBS	2011
KK2_091	NF	F1, KK population	na/GBS	2011
KK2_101	Male	F1, KK population	RAD/na	2011
KK2_104	Male	F1, KK population	RAD/GBS	2011
KK2_105	Male	F1, KK population	RAD/GBS	2011
KK2_106	NF	F1, KK population	RAD/GBS	2011
KK2_107	Female	F1, KK population	RAD/na	2011
KK2_108	NF	F1, KK population	RAD/GBS	2011
KK2_109	NF	F1, KK population	RAD/na	2011
KK2_111	NF	F1, KK population	na/GBS	2011
KK2_112	NF	F1, KK population	RAD/na	2011
KK2_113	Male	F1, KK population	RAD/GBS	2011
KK2_114	NF	F1, KK population	RAD/na	2011
KK2_117	NF	F1, KK population	RAD/GBS	2011
KK2_118	NF	F1, KK population	RAD/GBS	2011
KK2_119	NF	F1, KK population	RAD/GBS	2011
KK2_120	Female	F1, KK population	RAD/GBS	2011
KK2_121	Male	F1, KK population	RAD/na	2011
KK2_122	NF	F1, KK population	RAD/GBS	2011
KK2_123	NF	F1, KK population	na/GBS	2011
KK2_124	NF	F1, KK population	RAD/GBS	2011
KK2_125	NF	F1, KK population	RAD/GBS	2011
KK2_126	NF	F1, KK population	RAD/GBS	2011
KK2_128	NF	F1, KK population	RAD/GBS	2011
KK2_129	NF	F1, KK population	RAD/na	2011
KK2_130	NF	F1, KK population	RAD/GBS	2011
KK2_131	Male	F1, KK population	RAD/GBS	2011
KK2_132	NF	F1, KK population	RAD/na	2011
KK2_133	NF	F1, KK population	RAD/GBS	2011
KK2_134	NF	F1, KK population	RAD/GBS	2011
KK2_135	NF	F1, KK population	RAD/GBS	2011
KK2_136	NF	F1, KK population	RAD/GBS	2011
KK2_137	NF	F1, KK population	RAD/GBS	2011
KK2_138	NF	F1, KK population	na/GBS	2011
KK2_139	NF	F1, KK population	na/GBS	2011
KK2_140	NF	F1, KK population	RAD/GBS	2011
KK2_141	NF	F1, KK population	RAD/GBS	2011

KK2_142	NF	F1, KK population	RAD/GBS	2011
KK2_143	NF	F1, KK population	RAD/GBS	2011
KK2_144	Female	F1, KK population	RAD/GBS	2011
KK2_145	NF	F1, KK population	RAD/GBS	2011
KK2_146	NF	F1, KK population	RAD/GBS	2011
KK2_147	NF	F1, KK population	RAD/GBS	2011
KK2_148	NF	F1, KK population	RAD/GBS	2011
KK2_149	Male	F1, KK population	RAD/GBS	2011
KK2_150	NF	F1, KK population	RAD/GBS	2011
KK2_151	NF	F1, KK population	RAD/GBS	2011
KK2_152	Male	F1, KK population	RAD/GBS	2011
KK2_154	NF	F1, KK population	RAD/GBS	2011
KK2_155	NF	F1, KK population	RAD/GBS	2011
KK2_156	NF	F1, KK population	RAD/GBS	2011
KK2_157	Female	F1, KK population	RAD/GBS	2011
KK2_158	Male	F1, KK population	RAD/GBS	2011
KK2_159	Male	F1, KK population	RAD/GBS	2011
KK2_160	Male	F1, KK population	RAD/GBS	2011
KK2_161	NF	F1, KK population	RAD/GBS	2011
KK2_162	Male	F1, KK population	RAD/GBS	2011
KK2_163	NF	F1, KK population	RAD/GBS	2011
KK2_164	NF	F1, KK population	RAD/GBS	2011
KK2_165	Female	F1, KK population	RAD/GBS	2011
KK2_166	Female	F1, KK population	RAD/na	2011
KK2_167	NF	F1, KK population	RAD/GBS	2011
KK2_168	NF	F1, KK population	RAD/GBS	2011
KK2_169	Female	F1, KK population	RAD/GBS	2011
KK2_170	NF	F1, KK population	RAD/GBS	2011
KK2_171	Male	F1, KK population	RAD/GBS	2011
KK2_172	NF	F1, KK population	RAD/GBS	2011
KK2_173	Female	F1, KK population	RAD/GBS	2011
KK2_174	NF	F1, KK population	RAD/GBS	2011
KK2_175	Male	F1, KK population	RAD/GBS	2011
KK2_176	Male	F1, KK population	RAD/GBS	2011
KK2_177	NF	F1, KK population	RAD/na	2011
KK2_178	NF	F1, KK population	RAD/GBS	2011
KK2_179	Male	F1, KK population	RAD/na	2011
KK2_180	NF	F1, KK population	RAD/na	2011
KK2_181	Male	F1, KK population	RAD/GBS	2011
KK2_182	NF	F1, KK population	RAD/GBS	2011
KK2_183	NF	F1, KK population	RAD/GBS	2011
KK2_184	NF	F1, KK population	RAD/GBS	2011
KK2_185	Female	F1, KK population	RAD/GBS	2011
KK2_186	Male	F1, KK population	RAD/GBS	2011
KK2_187	NF	F1, KK population	RAD/GBS	2011
KK2_188	NF	F1, KK population	RAD/GBS	2011
KK2_189	NF	F1, KK population	RAD/GBS	2011
KK2_190	Male	F1, KK population	RAD/GBS	2011
KK2_191	NF	F1, KK population	RAD/GBS	2011
KK2_192	NF	F1, KK population	RAD/GBS	2011
KK2_193	NF	F1, KK population	RAD/GBS	2011
KK2_194	NF	F1, KK population	RAD/GBS	2011
KK2_195	Female	F1, KK population	RAD/GBS	2011
KK2_196	NF	F1, KK population	RAD/GBS	2011
KK2_197	Female	F1, KK population	RAD/GBS	2011
KK2_198	NF	F1, KK population	RAD/GBS	2011
KK2_199	Female	F1, KK population	RAD/GBS	2011
KK2_200	Male	F1, KK population	RAD/GBS	2011
KK2_201	Female	F1, KK population	RAD/GBS	2011
KK2_202	NF	F1, KK population	RAD/GBS	2011
KK2_203	NF	F1, KK population	RAD/GBS	2011
KK2_204	Female	F1, KK population	RAD/GBS	2011
KK2_205	Male	F1, KK population	RAD/GBS	2011
KK2_206	Male	F1, KK population	RAD/GBS	2011
KK2_207	Male	F1, KK population	RAD/GBS	2011

KK2_208	NF	F1, KK population	RAD/GBS	2011
KK2_209	NF	F1, KK population	RAD/GBS	2011
KK2_210	Female	F1, KK population	RAD/GBS	2011
KK2_211	NF	F1, KK population	RAD/GBS	2011
KK2_212	NF	F1, KK population	RAD/GBS	2011
KK2_213	Female	F1, KK population	RAD/GBS	2011
KK2_214	Female	F1, KK population	RAD/GBS	2011
KK2_215	NF	F1, KK population	RAD/GBS	2011
KK2_216	Male	F1, KK population	RAD/GBS	2011
KK2_217	Male	F1, KK population	RAD/GBS	2011
KK2_218	Male	F1, KK population	RAD/GBS	2011
KK2_219	Male	F1, KK population	RAD/GBS	2011
KK2_220	Male	F1, KK population	RAD/GBS	2011
KK2_221	Male	F1, KK population	RAD/GBS	2011
KK2_222	Female	F1, KK population	RAD/na	2011
KK2_223	NF	F1, KK population	RAD/GBS	2011
KK2_224	Female	F1, KK population	RAD/GBS	2011
KK2_225	Male	F1, KK population	RAD/na	2011
KK2_226	Male	F1, KK population	RAD/GBS	2011
KK2_227	NF	F1, KK population	RAD/GBS	2011
KK2_228	NF	F1, KK population	RAD/GBS	2011
KK2_229	Female	F1, KK population	RAD/GBS	2011
KK2_230	NF	F1, KK population	RAD/GBS	2011
KK2_231	NF	F1, KK population	RAD/na	2011
KK2_232	NF	F1, KK population	RAD/GBS	2011
KK2_233	Female	F1, KK population	RAD/GBS	2011
KK2_234	Female	F1, KK population	RAD/GBS	2011
KK2_235	NF	F1, KK population	RAD/GBS	2011
KK2_236	NF	F1, KK population	RAD/GBS	2011
KK2_237	NF	F1, KK population	RAD/GBS	2011
KK2_238	NF	F1, KK population	RAD/GBS	2011
KK2_239	NF	F1, KK population	RAD/GBS	2011
KK2_240	Female	F1, KK population	RAD/GBS	2011
KK2_241	Male	F1, KK population	RAD/GBS	2011
KK2_242	Male	F1, KK population	RAD/na	2011
KK2_243	Male	F1, KK population	RAD/na	2011
KK2_244	NF	F1, KK population	RAD/GBS	2011
KK2_245	NF	F1, KK population	RAD/na	2011
KK2_246	NF	F1, KK population	RAD/GBS	2011
KK2_247	Male	F1, KK population	RAD/GBS	2011
KK2_248	NF	F1, KK population	RAD/GBS	2011
KK2_249	Male	F1, KK population	RAD/GBS	2011
KK2_250	NF	F1, KK population	RAD/GBS	2011
KK2_251	Male	F1, KK population	RAD/na	2011
KK2_252	Female	F1, KK population	RAD/GBS	2011
KK2_253	Female	F1, KK population	RAD/GBS	2011
KK2_254	Female	F1, KK population	RAD/GBS	2011
KK2_255	NF	F1, KK population	RAD/na	2011
KK2_256	NF	F1, KK population	RAD/GBS	2011
KK2_257	NF	F1, KK population	RAD/GBS	2011
KK2_258	NF	F1, KK population	RAD/GBS	2011
KK2_259	NF	F1, KK population	RAD/GBS	2011
KK2_260	Male	F1, KK population	RAD/GBS	2011
KK2_261	Female	F1, KK population	RAD/GBS	2011
KK2_262	NF	F1, KK population	na/GBS	2011
KK2_264	NF	F1, KK population	na/GBS	2011
KK2_265	NF	F1, KK population	na/GBS	2011
KK2_266	Male	F1, KK population	na/GBS	2011
KK2_267	NF	F1, KK population	na/GBS	2011
KK2_268	NF	F1, KK population	na/GBS	2011
KK2_269	Male	F1, KK population	na/GBS	2011
KK2_270	Female	F1, KK population	na/GBS	2011
KK2_271	NF	F1, KK population	na/GBS	2011
KK2_272	NF	F1, KK population	na/GBS	2011
KK2_273	NF	F1, KK population	na/GBS	2011

KK2_274	NF	F1, KK population	na/GBS	2011
KK2_275	NF	F1, KK population	na/GBS	2011
KK2_276	NF	F1, KK population	na/GBS	2011
KK2_277	NF	F1, KK population	na/GBS	2011
VM_1	NA	F1, VM population	RAD/na	2009
VM_2	NA	F1, VM population	RAD/na	2009
VM_3	NA	F1, VM population	RAD/na	2009
VM_4	NA	F1, VM population	RAD/na	2009
VM_5	NA	F1, VM population	RAD/na	2009
VM_6	NA	F1, VM population	RAD/na	2009
VM_7	NA	F1, VM population	RAD/na	2009
VM_8	NA	F1, VM population	RAD/na	2009
VM_9	NA	F1, VM population	RAD/na	2009
VM_10	NA	F1, VM population	RAD/na	2009
VM_11	NA	F1, VM population	RAD/na	2009
VM_12	NA	F1, VM population	RAD/na	2009
VM_13	NA	F1, VM population	RAD/na	2009
VM_14	NA	F1, VM population	RAD/na	2009
VM_15	NA	F1, VM population	RAD/na	2009
VM_16	NA	F1, VM population	RAD/na	2009
VM_17	NA	F1, VM population	RAD/na	2009
VM_18	NA	F1, VM population	RAD/na	2009
VM_19	NA	F1, VM population	RAD/na	2009
VM_20	NA	F1, VM population	RAD/na	2009
VM_21	NA	F1, VM population	RAD/na	2009
VM_22	NA	F1, VM population	RAD/na	2009
VM_23	NA	F1, VM population	RAD/na	2009
VM_24	NA	F1, VM population	RAD/na	2009
VM_25	NA	F1, VM population	RAD/na	2009
VM_26	NA	F1, VM population	RAD/na	2009
VM_27	NA	F1, VM population	RAD/na	2009
VM_28	NA	F1, VM population	RAD/na	2009
VM_29	NA	F1, VM population	RAD/na	2009
VM_30	NA	F1, VM population	RAD/na	2009
VM_31	NA	F1, VM population	RAD/na	2009
VM_32	NA	F1, VM population	RAD/na	2009
VM_33	NA	F1, VM population	RAD/na	2009
VM_34	NA	F1, VM population	RAD/na	2009
VM_35	NA	F1, VM population	RAD/na	2009
VM_36	NA	F1, VM population	RAD/na	2009
VM_37	NA	F1, VM population	RAD/na	2009
VM_38	NA	F1, VM population	RAD/na	2009
VM_39	NA	F1, VM population	RAD/na	2009
VM_40	NA	F1, VM population	RAD/na	2009
VM_41	NA	F1, VM population	RAD/na	2009
VM_42	NA	F1, VM population	RAD/na	2009
VM_43	NA	F1, VM population	RAD/na	2009
VM_44	NA	F1, VM population	RAD/na	2009
VM_45	NA	F1, VM population	RAD/na	2009
VM_46	NA	F1, VM population	RAD/na	2009
VM_47	NA	F1, VM population	RAD/na	2009
VM_48	NA	F1, VM population	RAD/na	2009
VM_49	NA	F1, VM population	RAD/na	2009
VM_50	NA	F1, VM population	RAD/na	2009
VM_51	NA	F1, VM population	RAD/na	2009
VM_52	NA	F1, VM population	RAD/na	2009
VM_53	NA	F1, VM population	RAD/na	2009
VM_54	NA	F1, VM population	RAD/na	2009
VM_55	NA	F1, VM population	RAD/na	2009
VM_56	NA	F1, VM population	RAD/na	2009
VM_57	NA	F1, VM population	RAD/na	2009
VM_58	NA	F1, VM population	RAD/na	2009
VM_59	NA	F1, VM population	RAD/na	2009
VM_60	NA	F1, VM population	RAD/na	2009
VM_61	NA	F1, VM population	RAD/na	2009

VM_62	NA	F1, VM population	RAD/na	2009
VM_63	NA	F1, VM population	RAD/na	2009
VM_64	NA	F1, VM population	RAD/na	2009
VM_65	NA	F1, VM population	RAD/na	2009
VM_66	NA	F1, VM population	RAD/na	2009
VM_67	NA	F1, VM population	RAD/na	2009
VM_68	NA	F1, VM population	RAD/na	2009
VM_69	NA	F1, VM population	RAD/na	2009
VM_70	NA	F1, VM population	RAD/na	2009
VM_71	NA	F1, VM population	RAD/na	2009
VM_72	NA	F1, VM population	RAD/na	2009
VM_73	NA	F1, VM population	RAD/na	2009
VM_74	NA	F1, VM population	RAD/na	2009
VM_75	NA	F1, VM population	RAD/na	2009
VM_76	NA	F1, VM population	RAD/na	2009
VM_77	NA	F1, VM population	RAD/na	2009
VM_78	NA	F1, VM population	RAD/na	2009
VM_79	NA	F1, VM population	RAD/na	2009
VM_80	NA	F1, VM population	RAD/na	2009
VM_81	NA	F1, VM population	RAD/na	2009
VM_82	NA	F1, VM population	RAD/na	2009
VM_83	NA	F1, VM population	RAD/na	2009
VM_84	NA	F1, VM population	RAD/na	2009
VM_85	NA	F1, VM population	RAD/na	2009
VM_86	NA	F1, VM population	RAD/na	2009
VM_87	NA	F1, VM population	RAD/na	2009
VM_88	NA	F1, VM population	RAD/na	2009
VM_89	NA	F1, VM population	RAD/na	2009
VM_90	NA	F1, VM population	RAD/na	2009
VM_91	NA	F1, VM population	RAD/na	2009
VM_92	NA	F1, VM population	RAD/na	2009
VM_93	NA	F1, VM population	RAD/na	2009
VM_94	NA	F1, VM population	RAD/na	2009
VM_95	NA	F1, VM population	RAD/na	2009
VM_96	NA	F1, VM population	RAD/na	2009
VM_97	NA	F1, VM population	RAD/na	2009
VM_98	NA	F1, VM population	RAD/na	2009
VM_99	NA	F1, VM population	RAD/na	2009
VM_100	NA	F1, VM population	RAD/na	2009
VM_101	NA	F1, VM population	RAD/na	2009
VM_102	NA	F1, VM population	RAD/na	2009
VM_103	NA	F1, VM population	RAD/na	2009
VM_104	NA	F1, VM population	RAD/na	2009
VM_105	NA	F1, VM population	RAD/na	2009
VM_106	NA	F1, VM population	RAD/na	2009
VM_107	NA	F1, VM population	RAD/na	2009
VM_108	NA	F1, VM population	RAD/na	2009
VM_109	NA	F1, VM population	RAD/na	2009
VM_110	NA	F1, VM population	RAD/na	2009
VM_111	NA	F1, VM population	RAD/na	2009
VM_112	NA	F1, VM population	RAD/na	2009
VM_113	NA	F1, VM population	RAD/na	2009
VM_114	NA	F1, VM population	RAD/na	2009
VM_115	NA	F1, VM population	RAD/na	2009
VM_116	NA	F1, VM population	RAD/na	2009
VM_117	NA	F1, VM population	RAD/na	2009
VM_118	NA	F1, VM population	RAD/na	2009
VM_119	NA	F1, VM population	RAD/na	2009

[a] NF: not yet flowered in 2017, NA: not analyzed.

[b] Information of both RAD (ddRAD-Seq analysis) and GBS were given. na: not analyzed.
